# Mobile education builds resilience during shocks in five countries

**DOI:** 10.1038/s41586-026-10990-x

**Published:** 2026-09-09

**Authors:** Noam Angrist, Micheal Ainomugisha, Sai Pramod Bathena, Peter Bergman, Colin Crossley, Claire Cullen, Thato Letsomo, Moitshepi Matsheng, Rene Marlon Panti, Shwetlena Sabarwal, Tim Sullivan

**Affiliations:** 1 Blavatnik School of Government, University of Oxford, Oxford, UK; 2 What Works Hub for Global Education, Oxford, UK; 3 Youth Impact, Gaborone, Botswana; 4 Building Tomorrow, Kampala, Uganda; 5 Alokit, Hyderabad, India; 6 Department of Economics, University of Texas at Austin, Austin, TX USA; 7 Innovations for Poverty Action (IPA), Manila, Philippines; 8 World Bank, Washington, DC USA; 9 NewGlobe, Nairobi, Kenya

**Keywords:** Economics, Education, Society

## Abstract

Education systems need to withstand frequent shocks, including disease and climate events, which close schools for more than 222 million children^1^. During these emergencies, alternative models are needed to deliver education. However, rigorous evaluation of effective educational approaches in these settings is challenging and rare, especially across multiple countries. Here we present results from five randomized trials in India, Kenya, Nepal, the Philippines and Uganda to evaluate the provision of education in emergency settings. We test multiple scalable models of remote targeted tutoring instruction, comparing delivery by governments and non-governmental organizations (NGOs). Whereas text messages have mixed results, tutorials conducted via phone call show effectiveness across diverse settings. We find large and robust effect sizes on learning. These effects are highly cost-effective, and deliver approximately 4 years of high-quality instruction per US$100 spent. Results show large effects when delivered by government teachers or NGO instructors. Together, our results reveal that it is possible to strengthen the resilience of education systems, enabling education provision amid disruptions, and to deliver cost-effective learning gains across contexts and with governments.

## Abstract (web summary)

Remote phone-based targeted tutoring in five countries produced large, cost-effective learning gains during school closures, outperforming text messaging and working effectively when delivered by governments or non-governmental organizations to strengthen education resilience.

## Main

**Fig. 1:**
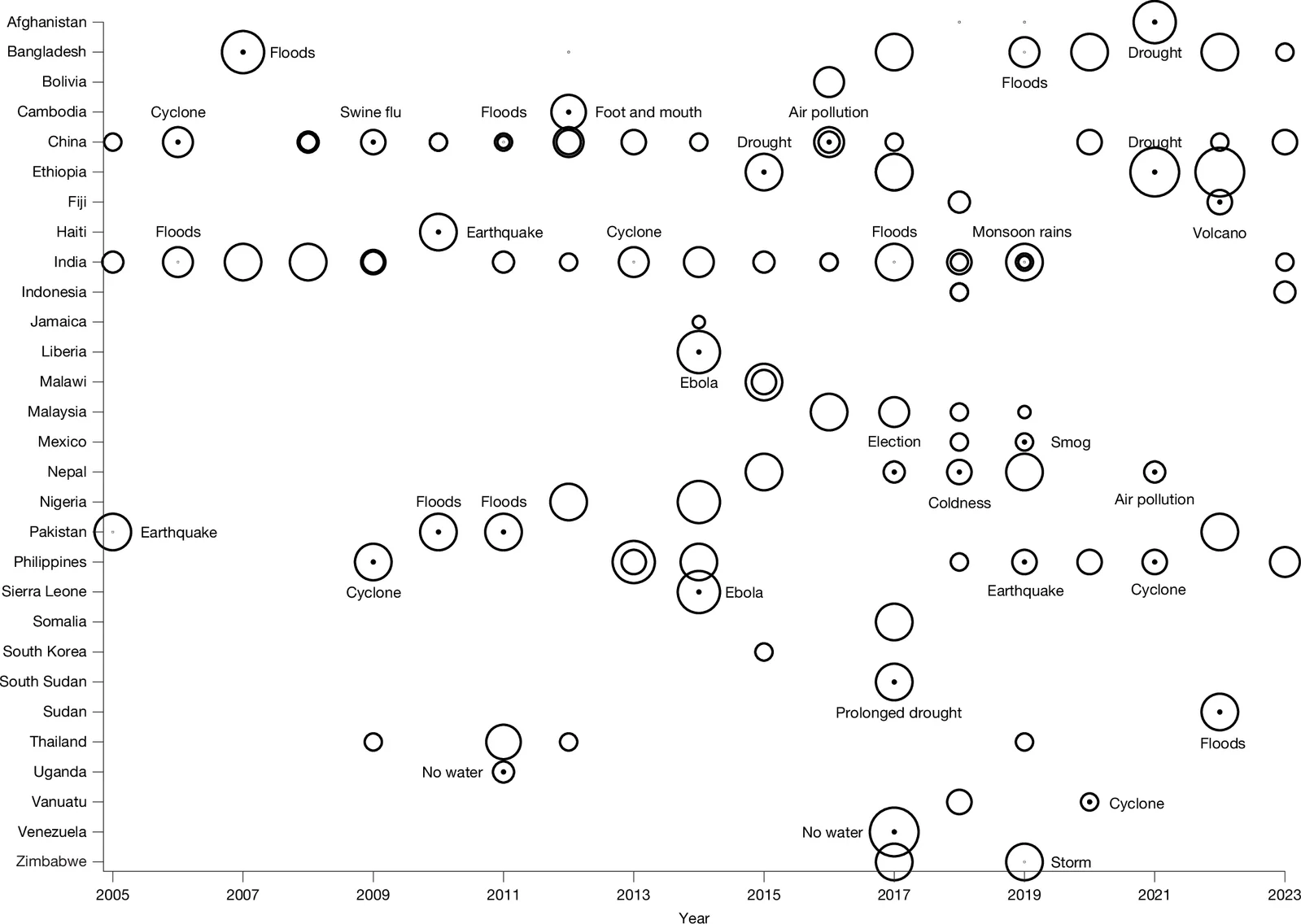
Documenting a set of education in emergencies between 2005 and 2023. The graph plots an index of the length of school closures and number of people affected by shocks that have disrupted schooling by country and year. The size of each bubble increases with the duration of school closure, the number of people affected, or both. Thicker or multiple circles represent multiple events in the same year and country. More information on the data compilation is available in the Supplementary Information. The data comprise compiled school closure information based on press releases of the United Nations Office for the Coordination of Humanitarian Affairs (OCHA) ReliefWeb, World Vision, United Nations Children’s Fund (UNICEF), the British Broadcasting Corporation (BBC) and other local outlets. Major disruptions were also identified through The International Disaster Database (EM-DAT).

More than 2 billion people live in countries that are affected by emergencies that frequently disrupt education. Although they are frequent and disruptive, education emergencies have historically been understudied. Here we present a new database that documents just how frequent and disruptive such shocks can be. Figure 1 documents the extent of school disruption for a selected set of emergencies over the past two decades.

Schools close for long periods during these emergencies, and learning loss can be substantial^2–5^. Education Cannot Wait, the United Nations’ Global Fund for education in emergencies, estimates that 222 million children experience regular schooling disruptions and are in active need of education in emergencies programmes^1^. In low-income countries, students lose on average 10% of the academic year owing to climate shocks alone^6^. Moreover, school breaks, although they are not emergencies, are another regular school disruption, and often results in substantial learning loss among low-resource households^7,8^. These shocks exacerbate a pre-existing learning crisis, leaving many students well behind grade-level expectations^9^. Fewer than half of primary-age students in low- and middle-income countries are able to read a story or perform two-digit mathematical operations^10^.

Resilient education systems need to promote learning across settings and to withstand frequent disruptions. Ideally, interventions that promote learning in emergencies should be low-cost, simple to implement and quick to deploy. They should also yield high take-up across different geographies and implementation models and should be targeted to children with diverse educational and cultural backgrounds. Understanding which approaches can be effective across these circumstances requires multi-context, multi-model studies. However, rigorous evaluation of approaches to deliver education in emergencies remains challenging and rare, especially across contexts and with governments.

Here we evaluate a set of education in emergencies programmes to promote learning during large-scale school disruptions, which affected more than 1 billion children worldwide. We conducted five randomized controlled trials, including multiple delivery models, such as NGO teacher aides and government teachers, to test scalability by government systems. Our trials were conducted across five countries: India, Kenya, Nepal, Uganda and the Philippines. In all settings, schooling was disrupted owing to the COVID-19 pandemic, and several of the countries in our study experienced some of the longest school closures in the world.

We use mobile phones to provide various educational interventions to primary school children. Mobile phones provide a platform that can reach households cheaply at scale in low-resource contexts^11^. Whereas less than 15% of households have access to the internet in low-income countries, more than 70% have access to mobile phones^12^. Moreover, mobile phones enable teachers to reach students at home even when school is disrupted, providing a resilient and flexible modality to provide education during emergencies. Mobile phones are increasingly relied on to provide aid via mobile money during humanitarian crises^13^, yet remain underutilized to provide mobile education. One treatment included a set of SMS messages, such as numeracy content provided weekly, as well as nudges to engage in educational activities. A second treatment provided additional weekly 20 min phone call tutorials for 8 weeks. The educational pedagogy was as essential as the mobile phone platform. Phone calls covered foundational numeracy and aimed to target instruction to student learning levels via regular low-cost, high-frequency assessments. This builds on both in-person and technology-based targeted instruction approaches^14–19^.

A proof of concept in Botswana showed that phone call tutorials were effective in promoting learning during initial pandemic school disruptions^20^. However, questions remain on whether this approach can be scaled across contexts and when delivered by governments—a pervasive challenge for social programmes^21,22^. To ensure scientifically robust findings, we need evidence across diverse settings and delivery models^23^. By conducting large randomized trials across five contexts as well as comparing models, such as NGO and government delivery, we contribute new evidence on effectiveness across diverse contexts and delivery models, key elements of scaling science^24^.

This study contributes to the literature in several areas. First, we contribute to the nascent experimental literature on education in emergencies. Substantial research has been carried out in this area, but much of it has been qualitative or with small samples. One exception is a randomized trial in which an NGO provided schooling in rural areas of conflict-affected regions in Afghanistan and found large effects on learning and closing of gender gaps^25^. We expand this literature by evaluating alternative, scalable forms of education beyond traditional in-person schools, such as remote learning, which is the only option during many emergencies when schools close. There is a robust literature documenting the cost of school disruptions due to teacher strikes^26^, earthquakes^2^, school holidays^7^ and COVID-19 (refs. ^4,27–31^). However, there is less evidence on effective approaches to stem these learning losses. We contribute evidence on scalable solutions to stem learning losses. A growing literature explores the effectiveness of remote tutoring, yet the results remain mixed^32–35^. Without a coordinated cross-country trial it is impossible to know whether differences in effects are due to the intervention or to the setting, since both vary simultaneously. For example, a recent study^33^ tested a related but different intervention in Sierra Leone—using a similar mobile phone platform but deploying a different pedagogy—and found no effect of mobile phone tutoring. In their study, the tutoring call was linked to a uniform national radio programme rather than targeting instruction based on student learning week-on-week—a key difference. To assess whether the approach works across contexts we cannot simply aggregate prior studies in which both intervention and context vary—we need to hold the intervention constant, while varying sites in a coordinated multi-site trial. We conduct such a coordinated five-country trial in this study.

We also contribute to a growing literature on external validity and scale. Recent examples show the extent of the scaling challenge, with many social programmes that initially worked in proof-of-concept studies no longer delivering impacts when scaled across settings or when delivered by governments^22,24,36,37^. In contrast to some existing studies, our results identify an education approach that appears to work well across contexts and when delivered by governments. One reason might be that we leverage a particularly scalable technology: mobile phones^11,38^. A second reason may be the generalizability of the underlying mechanisms tested in our study, which relate to other best practices in education. For example, tutoring has been shown to be one of the most effective, although expensive, approaches to improving learning in high-income settings^39,40^. The phone call tutorials provide a cheaper version of tutoring that is applicable in low- and middle-income contexts. In addition, since the phone calls are one-on-one and have frequent learning assessments, they enable targeted instruction to every child’s level, another approach shown to consistently improve learning^15–17,19^. Our study further advances the external validity and scale literature by conducting randomized trials across five countries in a literature where very few randomized controlled trials are multi-country studies. We also advance the scale literature by introducing experimental evaluation both of government relative to NGO delivery as well as randomizing those teachers who implement to evaluate effects on ongoing teacher practices. These experimental variations enable us to assess questions of effectiveness within government systems.

Finally, we contribute to the global education literature, with a focus on improving learning outcomes. Over the past several decades, education enrolments have improved worldwide, yet learning has barely budged^10,41,42^. Growing evidence reveals that popular input-only reforms, such as general teacher training, provision of computers, or school grants, are not enough to improve learning. By contrast, approaches that improve the quality of teaching, such as teaching at the right level and structured pedagogy, can generate large improvements in learning^43–46^. The learning gains from the phone-based tutorials tested in this study can deliver up to 4 years of high-quality schooling per US$100 (ref. ^47^). These types of highly cost-effective approaches can more easily scale and help address a persistent global learning crisis in low- and middle-income contexts.

## Main results

We report results on a set of rich, harmonized data across five countries using both baseline and endline surveys. Questions included a learning assessment with the child and several additional child indicators, such as well-being, self-perceptions and non-cognitive skills. We also collected parental beliefs and behaviours. A portion of the survey was conducted with the parent, and learning outcomes were collected by directly assessing the child over the phone. A set of common questions were included in the baseline and endline surveys and across countries for comparability.

We report results from large-scale randomized controlled trials across five countries: India, Kenya, Nepal, the Philippines and Uganda. In Nepal and the Philippines, we include government and NGO delivery treatment arms. All countries except India also included a comparison SMS-only treatment relative to a phone call plus SMS treatment. Randomization enables identification of causal effects of the education in emergency interventions studied. Further details on the data and experimental methods are provided in the Methods section. Further details on the contexts and interventions are provided in the Supplementary Information.

**Fig. 2:**
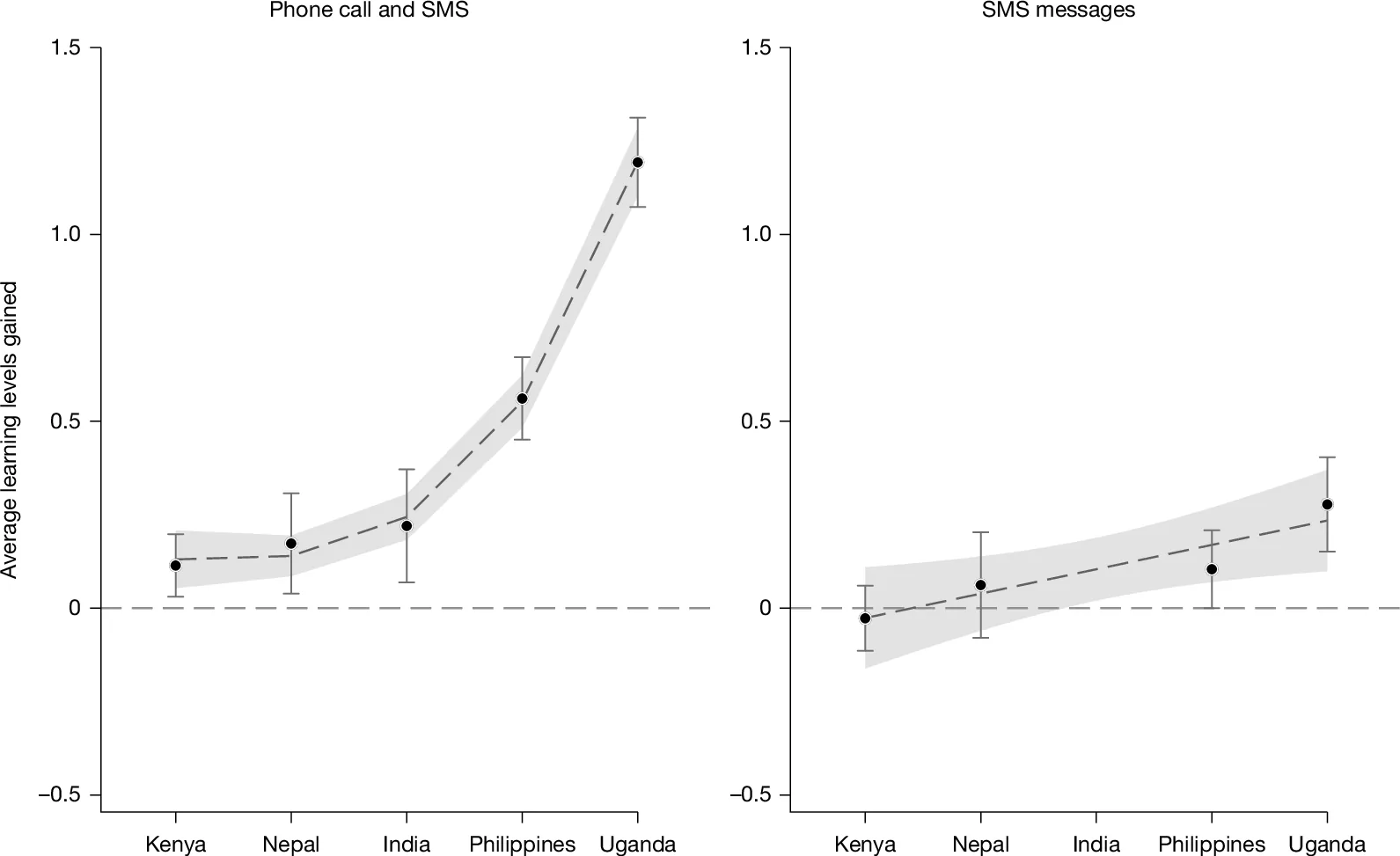
Main learning results across countries and treatments. Average raw unit operational learning levels gained in treatment groups relative to control groups. Treatment effects on learning outcomes are reported by country and treatment group with 90% confidence intervals within each country. Countries are ordered by the timing of the study, with Kenya first and Uganda last. The curve represents a fractional polynomial fit (phone arm) and a linear fit (SMS arm) of average learning levels gained against the order of the country in the study, each with a 90% confidence band. ‘Learning levels gained’ refers to the number of operations gained, with each operation scored as an additional level gain (beginner, 0; addition, 1; subtraction, 2; multiplication, 3; division, 4). Data are presented as treatment effects relative to the control group with ±90% confidence intervals. Sample sizes: India (*n* = 708), Kenya (*n* = 1,985), Nepal (*n* = 2,678), the Philippines (*n* = 2,469) and Uganda (*n* = 1,308). Further details on related results are presented in Extended Data Table 1.

Our main results are shown in Fig. 2 and show positive effects for the combined phone and SMS treatment in all countries, with increasing effectiveness over time as the trials progressed; for the SMS-only treatment, effects are more mixed across countries. Effects are further elaborated on in Extended Data Table 1, where we report effects across contexts on the number of operations gained and the highest proficiency attained. We also show pooled average effects across all five countries. Column 1 in Extended Data Table 2 shows that for the combined phone and SMS group, there was a 0.321 s.d. (*P* < 0.001) increase in the average numerical operation across all 5 countries. SMS effects are smaller on average, with a 0.078 s.d. effect (*P* = 0.006). As shown in Fig. 2 and Extended Data Table 1, SMS messages only have a detectable statistically significant effect in a subset of contexts (Uganda and the Philippines). These results suggest that SMS messages can work in contexts with the largest need. However, live phone calls may be necessary to strike the balance of being intensive enough to deliver impact that can be sustained and scaled across diverse contexts while remaining cheap and scalable.

In addition, although effects of phone call tutorials are consistently positive, they vary in magnitude. Results are largest in later trials and in countries that experienced the longest school closures: Uganda and the Philippines. As shown in Extended Data Table 1, students in Uganda gain more than a full operation level (1.193 levels, *P* < 0.001). This translates to substantial proficiency gains, with 34.4 percentage point gains (*P* < 0.001) on the highest proficiency level. In the Philippines phone tutorials yield 0.56 level gains (*P* < 0.001) and a 13 percentage point increase (*P* < 0.001) in students reaching the highest operation level. In both countries, students were out of school for almost 2 years and the counterfactual for the control group was very limited schooling. These countries were also the later trials, with implementation fidelity improving over time. We discuss both of these mechanisms in more depth later on. Results are robust to the inclusion of a suite of control variables.

**Extended Data Fig. 1:**
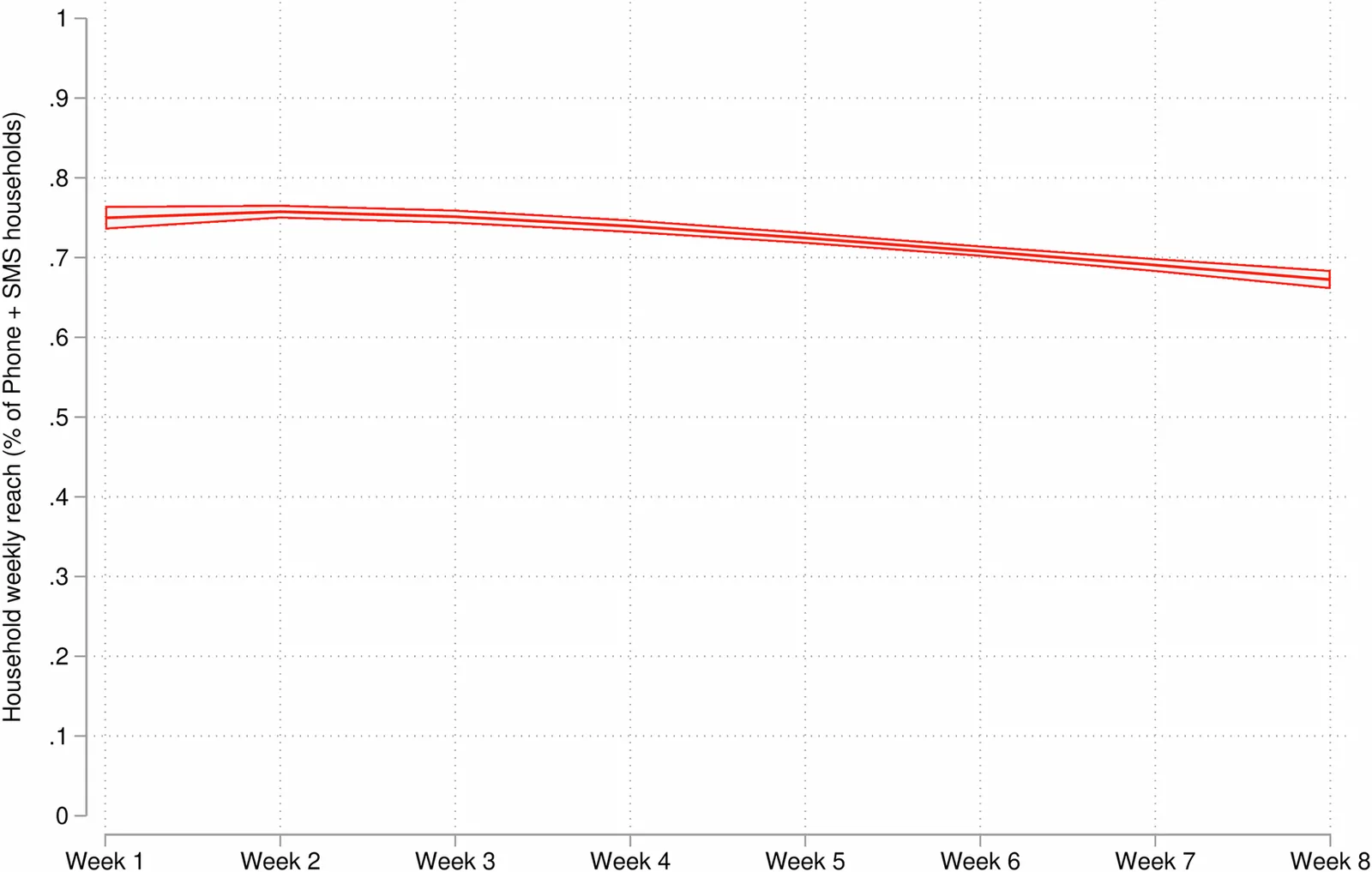
Weekly Household Engagement in Phone Calls. **Notes:** This figure shows average weekly engagement of households in the phone call and SMS arm. Each week, engagement is coded as 1 if the household answered the call and engaged. This includes data from Nepal, Uganda and the Philippines where we have detailed and easily comparable weekly monitoring data. The shaded band represents ± 95 percent confidence intervals.

Mobile phone calls saw high take-up and high engagement throughout the programme, consistent with high impact. Detailed monitoring data shows high week-on-week engagement across sites ranging from 70% to 80%, as shown in Extended Data Fig. 1, even amid disruptions and across diverse low-resource settings. This highlights that the programme reached households using a platform that they found easy and convenient to access on a regular basis. This contrasts with the low rates of take-up of other platforms, with few households typically taking up alternative delivery modes such as online, television or radio educational resources.

For both the SMS group and the combined phone and SMS group, the magnitude of the impact and statistical significance are robust to different estimation approaches. In Extended Data Table 2, we include baseline controls and country and grade fixed effects (column 2), and weight results by country arm (columns 3 and 4). We also adjust for multiple hypothesis testing in Supplementary Table A5, including sharpened *q* values to address the false discovery rate^48^. Since we have one outcome and two treatment arms, we consider two joint null hypotheses simultaneously. Estimates remain consistent.

Extended Data Table 3 and Supplementary Table A7 show learning outcomes across several proficiencies. Results show that the combined phone and SMS arm increases the share of students who get division problems correct by 13.5 percentage points (*P* < 0.001)—a 92% increase in division (from a control mean of 14.6%). These results by proficiency demonstrate that learning improved substantially in absolute terms, in addition to s.d. gains, and across a range of proficiencies. We also find increases in the share of students who are able to perform higher-order competencies, such as fractions (6.4 percentage point gains, *P* < 0.001). Since these competencies were not directly taught during the intervention, this reveals learning extended beyond familiarity with the content taught. These results further reveal potential dynamic complementarities in skill formation, showing that benefits of learning basic numeracy accrue to learning additional higher-order skills^49^.

These results are large relative to the effectiveness of a typical education intervention. A recent review found that the median effective intervention yielded 0.10 s.d. gains in learning^50^. Moreover, a review of 150 interventions found that more than half of education interventions do not work at all^47^. These reviews put our results in context, with phone call tutorials being three times as effective as the median education intervention. Moreover, effects are even larger in these multi-country studies than in the Botswana proof-of-concept study, where learning improved by 0.12 s.d. (ref. ^20^). This result contrasts with prior literature showing that proof-of-concept studies rarely scale successfully to new contexts^21^ or experience diminishing returns^37^. Rather than finding diminishing returns, we find that results improve as the approach is adapted, scaled and tested across contexts. We explore potential explanations later in the paper, including learning from experience as well as higher need for the intervention over time.

In Table 1, we show results for the subset of countries (Nepal and the Philippines) where we randomized delivery models to test scalability within government systems. Column (1) shows the pooled results: both government teachers and NGO instructors are effective at improving student learning, with government teachers improving learning by 0.40 operation levels across both contexts, and NGO teacher aides improving learning by 0.30 operation levels (*P* < 0.001 for both). These results demonstrate that education in emergencies programmes can be effectively implemented by government teachers, and not only by NGO implementers.

**Table 1:** Learning outcomes for government delivery

	All government arms	Nepal	Philippines
	(1) Operations gained	(2) Operations gained	(3) Operations gained
SMS messages	0.048	0.072	0.060
	(0.049)	(0.080)	(0.061)
	[0.326]	[0.367]	[0.324]
Phone call and SMS: NGO	0.296***	0.132	0.510***
	(0.057)	(0.088)	(0.073)
	[0.000]	[0.134]	[0.000]
Phone call and SMS: government	0.399***	0.213**	0.629***
	(0.061)	(0.087)	(0.086)
	[0.000]	[0.015]	[0.000]
Observations	5,147	2,678	2,469
Control mean	1.527	1.689	1.388
*P* value: NGO versus government	0.110	0.356	0.209
Country fixed effects	Yes	No	No
Countries included	Nepal, Philippines	Nepal	Philippines

Learning outcomes comparing implementation delivery models (NGO and government). Learning refers to how a child scores on four basic numeracy options: no operations correct, addition, subtraction, multiplication and division (measured on a scale of 0–4). This variable takes the value of 0 for students who got zero operations correct, 1 if students got addition correct, 2 if subtraction was correct, 3 if multiplication was correct, and 4 if students got division correct. Column (1) pools both countries in which we tested implementation scale-up in government systems. Column (2) shows the results for Nepal alone, and column (3) shows results for the Philippines alone. Standard errors are in parentheses and * P* values are in square brackets. *P < 0.10; **P < 0.05; ***P < 0.01.

These results show substantial effectiveness when delivered by governments. Prior literature has found that programmes often work when delivered by NGOs but are not replicated when scaled by governments. For example, in Kenya, contract teachers improved learning, but when delivered by the government, the programme effectiveness waned^36^. A large-scale government teacher training programme in Nepal similarly found no effect, largely owing to poor implementation^51^. Our results build on this literature, providing an alternative view: government delivery can achieve large learning gains. This is consistent with other studies that have refined interventions over time to facilitate government adoption^15,17^.

Heterogeneous treatment effects, shown in Table 2, reveal that the programme is broadly effective for students across the distribution of baseline learning levels. This relatively even impact across students’ starting conditions, or characteristics such as baseline learning levels or gender, is consistent with the mechanism of teaching at the right level. Targeted teaching is designed so that instruction meets children where they are regardless of their grade or age or other characteristics. Thus, broad-based learning gains are likely to be due to the fact that the programme was highly and effectively targeted, benefiting children across the distribution. We also find slight heterogeneity by parental education. Column (1) in Table 2 suggests that the programme worked particularly well for students where the caregiver had lower levels of formal education (primary education or less). This suggests that results are strongest when there are fewer alternative education support systems at home. This also reveals that even in low-literacy contexts, parents can be effective conduits for quality instruction.

**Table 2:** Heterogeneity

	Caregiver education	Gender	Baseline level
	(1) Operations gained	(2) Operations gained	(3) Operations gained
SMS messages	−0.013	0.136***	0.277***
	(0.148)	(0.052)	(0.077)
	[0.933]	[0.008]	[0.000]
Phone call and SMS	0.288**	0.427***	0.219**
	(0.139)	(0.047)	(0.095)
	[0.038]	[0.000]	[0.021]
Primary	0.026		
	(0.110)		
	[0.812]		
Secondary plus	0.446***		
	(0.103)		
	[0.000]		
SMS messages × primary	0.079		
	(0.169)		
	[0.643]		
SMS messages × secondary plus	0.114		
	(0.154)		
	[0.462]		
Phone call and SMS × primary	0.275*		
	(0.160)		
	[0.085]		
Phone call and SMS × secondary plus	0.112		
	(0.145)		
	[0.439]		
Is female		0.093*	
		(0.048)	
		[0.052]	
SMS messages × is female		−0.070	
		(0.070)	
		[0.321]	
Phone call and SMS × is female		−0.024	
		(0.064)	
		[0.703]	
Baseline level			0.360***
			(0.050)
			[0.000]
SMS messages × baseline level			0.007
			(0.038)
			[0.848]
Phone call and SMS × baseline level			0.006
			(0.035)
			[0.857]
Observations	8,028	9,148	9,148
Control mean	1.726	1.726	1.726
*P* value: SMS versus phone call plus SMS	0.048	0.000	0.627
Countries included	All 5	All 5	All 5
Country fixed effects	Yes	Yes	Yes

Heterogeneous treatment effects on learning. Column (1) shows the results between having caregivers who reached primary education or less and caregivers who reached secondary education or more (secondary plus). Column (2) shows the results between boys and girls. Column (3) shows results across baseline learning levels including country-specific baseline level interactions. To enable estimation for the full sample of endline learning results, baseline learning is imputed for the random sample, and not assessed based on the average. Standard errors are in parentheses and *P* values are in square brackets. *P < 0.10; **P < 0.05; ***P < 0.01.

We embedded an additional randomized study allocating a random subset of teachers to deliver the phone call tutorials in Nepal out of a list of hundreds of eligible government teachers. This additional experimental variation enables detection of the impact of delivering the programme on teachers’ beliefs and practices—a measure of potential spillover effects into the education system that can persist through teachers beyond the intervention. In addition, teacher randomization enables generalization to the broader set of eligible government teachers. Thus, results capture government delivery impacts which do not hinge on implementers being highly selected, and effects are likely to translate to typical government teachers in the education system.

Table 3 shows that teacher practices shift; teachers are 9.3 percentage points more likely to target their feedback to students’ learning level (*P* < 0.05). Teachers are also more likely to get parents involved in education (*P* < 0.1). We further find large effects on teacher perceptions that they were able to help students learn, as well as their desire to teach, with a 15.8 percentage point gain in wanting to be a teacher if they could make the choice again (*P* < 0.1 and *P* < 0.01, respectively). Although these results are reported by teachers and should be interpreted with caution, they suggest that delivering effective programmes could unlock a virtuous cycle within government education systems, in turn motivating teachers to want to teach and to improve their teaching practice further.

**Table 3:** Changing teacher beliefs and practices

	Teacher practices		Teacher beliefs	
	(1)	(2)	(3)	(4)
	Get parents involved	Targets to student level	Would be teacher again	Helped students’ learning
Teacher implemented call	0.088*	0.093**	0.158***	0.106*
	(0.050)	(0.046)	(0.045)	(0.058)
	[0.076]	[0.043]	[0.000]	[0.067]
Observations	290	290	290	290
Control mean	0.721	0.769	0.735	0.408

Treatment effects on teacher practices and beliefs. This includes government teachers in Nepal who were randomly assigned to implement the phone call programme. We successfully spoke to 83% of the government-employed mathematics teachers who were in the schools identified by local governments. Of the eligible grade 3–5 mathematics teachers that we spoke to, 81% expressed interest in participating in the study. Of this eligible pool of 301 government teachers, 50% were randomly selected to implement the programme. The endline response rate was 96% and was balanced across the treatment and control groups. Column (1) shows effects on teachers’ beliefs that they can get parents involved in their child’s education. Column (2) shows effects on teachers saying that they tailor student feedback based on students’ individual understanding and skill level. Column (3) shows effects on teachers saying if they had to choose their profession again, they would still be a teacher. Column (4) shows effects on teachers saying they believe they helped their students with their mathematical skills. These are all dummy variables that are coded as 1 if respondents strongly agreed on a Likert scale. Standard errors are in parentheses and *P* values are in square brackets. *P < 0.10; ** *P* < 0.05; ***P < 0.01.

**Extended Data Fig. 2:**
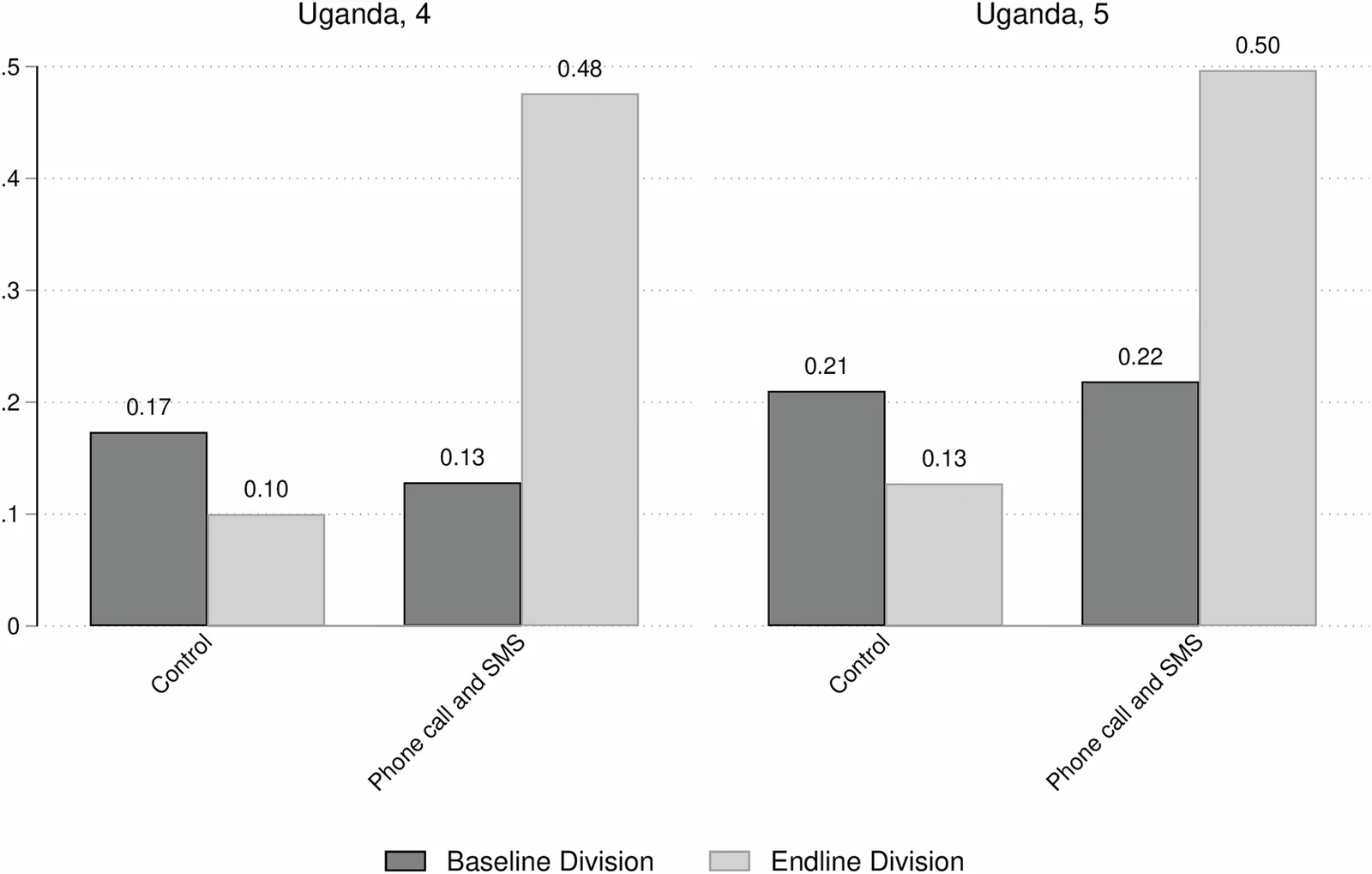
Learning Losses & Gains - Can Divide, Uganda, by Grade. **Notes:** This figure shows the proportion of Grade 4 and Grade 5 students in Uganda that know how to correctly perform division. The shades distinguish the baseline and endline proportion. In each panel, the two bars on the left show the outcomes for students who did not receive the intervention; the two on the right show the outcomes for those who received the SMS exercises and phone call tutoring. The figure shows that there is significant learning loss for control students between baseline and endline. It also shows that there is only a modest increase in the year-on-year share of students at division-level proficiency between grades 4 and 5 in the status quo. Finally, it shows the share of students able to perform division in the treatment group both recovers learning loss and far exceeds the year-on-year progress in division proficiency, with treated students overtaking the subsequent grade’s proficiency level at baseline.

**Extended Data Fig. 3:**
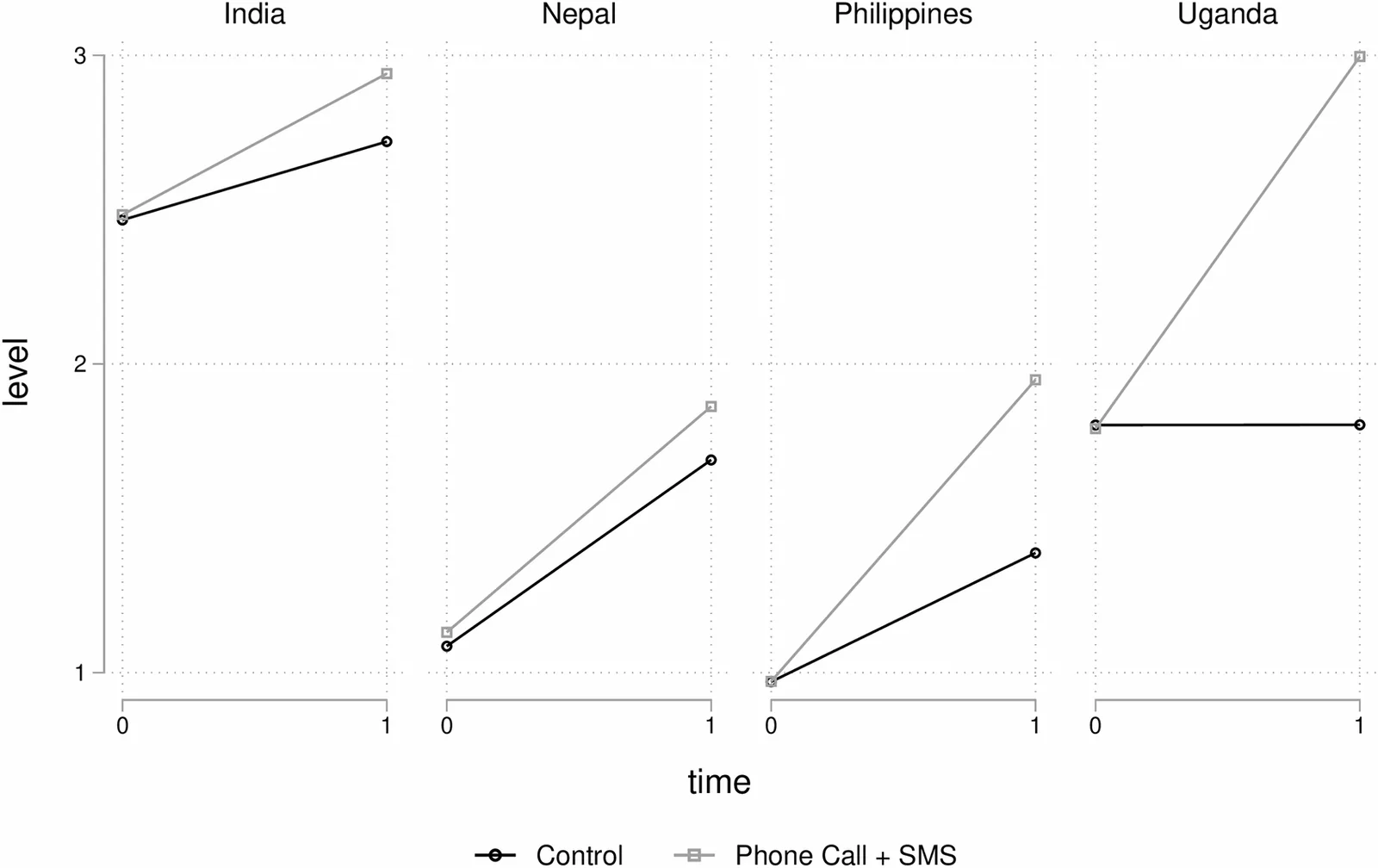
Learning Trajectories. **Notes:** This figure shows baseline and endline operation levels for the phone call tutorial treatment arm by country to showcase learning gains relative to status quo learning trajectories in the control group. We include all countries except Kenya since Kenya did not use an identical learning test at baseline as at endline. All other countries – India, Nepal, Philippines and Uganda – used the same learning test at baseline and endline. Endline sample sizes are as follows: India (n = 708), Nepal (n = 2,678), Philippines (n = 2,469), and Uganda (n = 1,308). More detail on related results can be found in Extended Data Table 1.

We examine counterfactual learning trajectories in Extended Data Figs. 2 and  3 to contextualize gains in the treatment groups. In Uganda, where effects are largest, about 17% of grade 4 students can divide at baseline in the control group. At endline, only 10% can divide, showing substantial learning loss. In the treatment group, 48% of grade 4 students can divide at endline, fully recovering learning loss due to school closures. Moreover, only 21% of grade 5 students in the control group at baseline can divide, revealing that grade 4 students in the treatment group surpass grade 5 students in the status quo. Thus, not only does the intervention facilitate full recovery of learning loss, it exceeds typical learning trajectories by nearly an order of magnitude. In other countries, learning does not deteriorate, but simply increases slowly. The intervention accelerates learning in these contexts relative to the counterfactual learning trajectory.

In the Supplementary Information, we include several additional results. We analyse effectiveness during another education emergency—a typhoon that struck the Philippines during the study. We explore additional margins of impact, including parent beliefs, student beliefs and non-cognitive skills. Finally, we explore the potential for crowd-out, to test whether tutoring time displaces alternative productive activities. Overall, we find continued effectiveness during an additional education emergency, several margins of impact beyond learning outcomes, and limited crowd-out.

## Mechanisms across contexts

We explore variation in effects of phone call tutorials, which worked consistently but heterogeneously, across diverse contexts. Analysing mechanisms to explain effectiveness across contexts is challenging, and should be done with caution, since multiple factors vary simultaneously. Table 4 includes a summary of key facts per country trial. We conduct exploratory analysis using suggestive descriptive data and observable systematic patterns. We focus on two mechanisms with particular variation across contexts: rates of targeted instruction and counterfactual conditions. Various additional margins vary across contexts, but do not vary systematically or consistently enough to facilitate credible comparisons. For example, in Kenya, younger students were taught but this varies only once, rather than systematically across settings.

**Table 4:** Trial description

	India	Kenya	Nepal	Philippines	Uganda
Sample size	850	6,724	3,732	3,492	2,138
Randomly selected endline sample size	765	3,556	3,351	3,164	1,495
Student grades	3–5	1–2	3–5	3–4	3–5
NGO delivery	✓	✓	✓	✓	✓
Government delivery	—	—	✓	✓	—
Unit of randomization	Household	School-grade cluster	Household	NGO: household; government: school-grade cluster	Household
Stratification variables used in block randomization	Student baseline level, school, female	School	Local government, parent perception of student baseline level	NGO: region, student baseline level; government: school, grade, student baseline level	Student baseline level, attends government school, previous education programme participation
Included combined phone call and SMS treatment	✓	✓	✓	✓	✓
Included SMS-only treatment	✗	✓	✓	✓	✓
Implementer type	NGO (Alokit)	NGO public–private partnership (NewGlobe)	Government (Ministry of Education), World Bank, NGOs (Teach for Nepal, Street Child)	Government (Department of Education), research NGO (Innovations for Poverty)	NGO (Building Tomorrow)
Same teachers as school instruction	—	✓	—	✓ – government delivery	—
Number of weeks of implementation	8	8–12	8	8	8
Dates	Apr 2021–Jul 2021	Dec 2020–Apr 2021	Jan 2021–Jul 2021	Aug 2021–Jul 2022	Oct 2021–Jan 2022
Administrative units in study	1 state	30 counties	All 7 provinces	3 regions	9 districts

Summary of key features of the intervention across countries. Implementers of the interventions included government education ministries and NGOs.

### Platform and pedagogy

**Extended Data Fig. 4:**
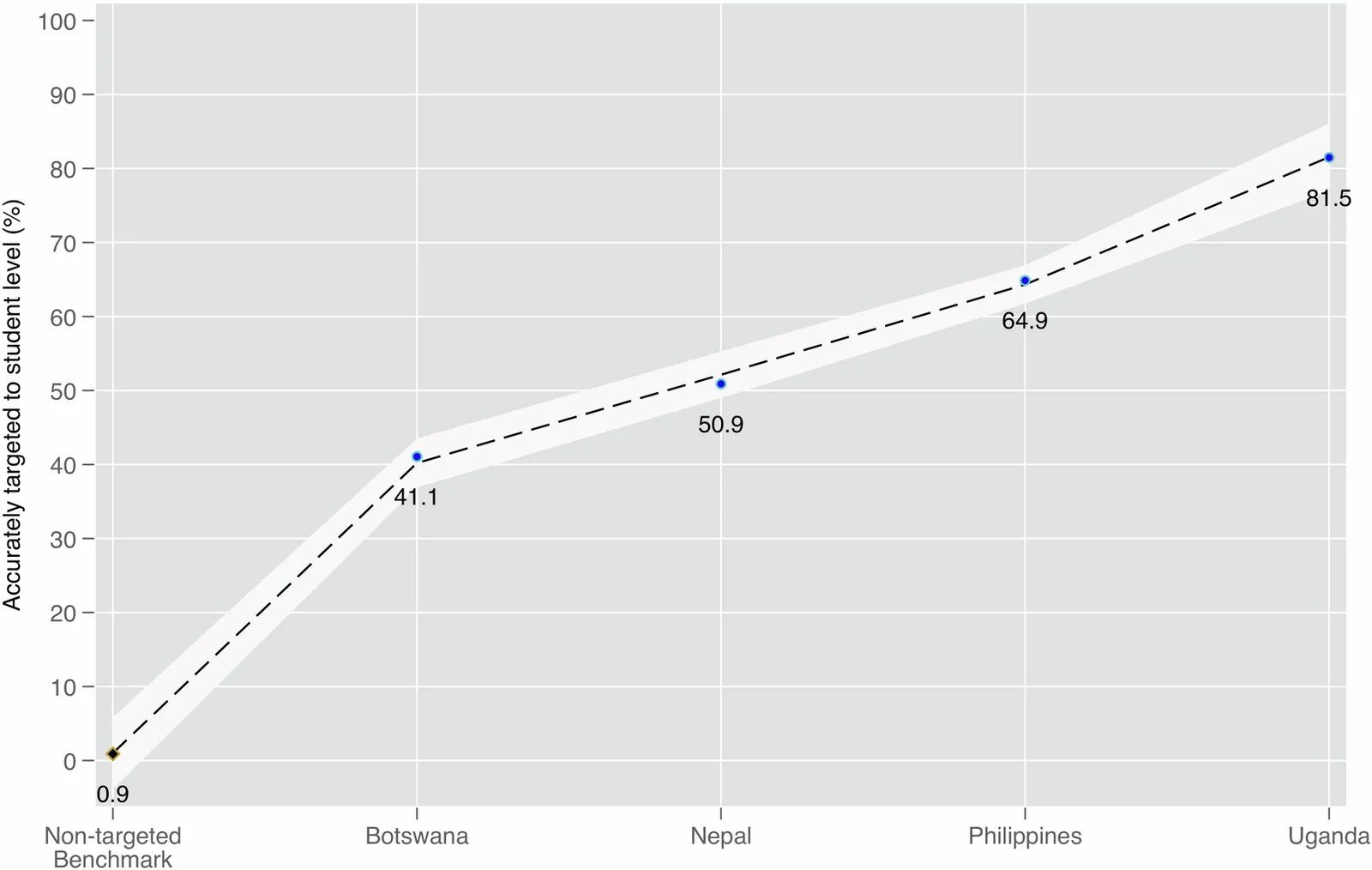
Learning Curve – Improved Implementation and Targeted Instruction Across Trials. **Notes:** This figure uses monitoring data to estimate how targeted instruction was across studies where enough monitoring data existed. Targeted instruction is defined as whether instructors taught students at their level each week, and is then averaged across all eight weeks. For example, if a child did not know addition and they were taught addition, the instruction was well targeted; if a child did know addition but was still taught it rather than moving on to subtraction, instruction was not well targeted. Estimates are also included from the Botswana proof-of-concept study. Benchmark estimates from control groups of teaching at the right level studies in India are also included to show how often instruction is targeted in the status quo. These estimates track learning outcome progress, with learning improving from Botswana (where instruction was targeted 41.1 percent of the time) to Nepal (where instruction was targeted 50.9 percent of the time) to the Philippines (where instruction was targeted 64.9 percent of the time) to Uganda (where instruction was targeted 81.5 percent of the time). The shaded band represents ± 90 percent confidence intervals. Reproduced from ref. ^52^; copyright 2026 American Economic Association.

In addition to leveraging a scalable platform—mobile phones—the programme pedagogy of targeted educational instruction, often referred to as ‘teaching at the right level’, was a core dimension of implementation fidelity. Phone call tutorials were designed to target instruction to children’s learning levels. We find that variation in effect across trials is consistent with variation in targeted instruction. Although results show that phone calls were consistently effective across countries, the magnitude of effects increases in tandem with the order of the trial: Kenya, followed by Nepal, India, the Philippines and finally Uganda. This order of effectiveness tracks the degree of targeted instruction. Detailed monitoring data provides evidence of increasing implementation fidelity as the trials progress. For example, the accuracy of targeted educational instruction increases from a starting point of 50.9% of students in Nepal to 81.5% on average in Uganda^52^ (Extended Data Fig. 4). These data reinforce the importance of targeted instruction. As the targeting mechanism improved study after study, this coincided with the programme becoming more effective. These results suggests the ‘voltage drop’ in effectiveness over time^21^—all too common when scaling—is not inevitable. Equipped with monitoring data and coordinating mechanisms to learn across trials, these results show that programmes have the potential to experience a ‘learning curve’ with improved fidelity (such as having ever more targeted instruction) and thus higher impact over time as they are scaled and implemented in new contexts. In addition to the trial-by-trial improvement in targeting accuracy, we find high rates of targeting accuracy through both NGO and government delivery. This consistent targeting accuracy across implementer types may explain the high effectiveness of both NGO and government teachers. It also adds evidence regarding the importance of targeting accuracy as a mechanism for impact^52^.

These data suggest that effective targeting pedagogy has an important role in programme effectiveness. This mechanism may explain why the approach tested worked across diverse contexts. Consistent with the importance of these mechanisms, a phone education programme that did not rely on these principles had less impact: phone calls in Sierra Leone that delivered non-targeted lessons based on a mass radio programme to students did not improve learning^33^. In the Sierra Leone study, the approach used an accessible platform without targeted pedagogy. To successfully scale across different contexts, ensuring that targeted instruction is in place is likely to be key to improving learning.

### Counterfactual conditions

Extended Data Table 4 shows several statistics characterizing counterfactual conditions. We observe clear patterns that show more severe counterfactual conditions coinciding with larger impacts. In Uganda and the Philippines, the settings with the highest impacts, we also see the most severe conditions, with more disruption and fewer alternative educational instruction options. Uganda and the Philippines have the highest degree of disruption in general and during the study, experiencing up to 2 years of disruption, with disruption occurring during the entire study period. They also have the largest share of lagging students, with 9% or less of students reaching the top operation level in the status quo. Similarly, we see that Uganda and the Philippines have low rates of outside options, with less than 22% of households saying they received any calls from educators in the past few weeks.

Although these counterfactual conditions do not follow an exact rank order with effect size, the broad patterns are consistent—more severe conditions map broadly to larger effect sizes in Uganda and the Philippines. This suggests the interventions studied are likely to add value particularly in the most disrupted education in emergency settings.

## Cost-effectiveness

An important feature of the education in emergencies approaches tested in this study is that they have low cost. The primary tool required to implement the programme is a mobile phone, a device that most households have access to, even in low-resource settings^53^. Since the approach builds on existing household infrastructure, the main costs are related to content delivery and connecting with families, which are often marginal, such as airtime for phone calls. In addition to being low cost, the approach has low procurement needs, an attractive feature for governments.

We carefully collected cost data in each trial. Our estimates suggest an average cost per child of the phone call and SMS tutorials of about US$11 per child. This estimate is even cheaper than estimates in the proof-of-concept study in Botswana owing to economies of scale as the approach has scaled up, including cost savings such as use of existing pedagogical material, shorter and more efficient training, and streamlined data monitoring systems. We benchmark the impacts of the programme against other education programmes using a variety of approaches. First, we compare raw estimates of effectiveness and cost with similar programmes such as tutoring and targeted instruction. Phone call tutorials simulate the benefits of one-on-one tutoring, which has been shown to be one of the most effective educational approaches^39^. However, many tutoring programmes have high cost. For example, a prominent tutoring programme yielded 0.19 to 0.31 s.d. learning gains at a cost of US$2,500 per child. In low- and middle-income settings, phone calls could provide similar or larger impacts two orders of magnitude more cheaply, enabling scale-up across diverse settings. The SMS-only treatment, when effective, can be extremely cost-effective, at 41.1 learning-adjusted years of schooling (LAYS) per US$100. However, whereas phone and SMS effects were consistent across all countries, the effects in the SMS-only arm varied across contexts. Given this, it seems plausible that the SMS-only intervention represents a cost-effective option in extreme education emergencies where, for example, calls are not an option or schooling is disrupted for such an extended period of time such that any provision of content is substantially better than the status quo.

Second, we use LAYS, a cost-effectiveness measure in education that has been estimated for over 150 impact evaluations in low- and middle-income countries; each LAYS can be interpreted as a high-quality year of schooling gained. We find the programme yields 3.6 LAYS per US$100, ranking in the top 10 out of 150 education interventions reviewed^47^. This result highlights the potential for phone call tutorials to deliver value to education systems and students in a broad array of contexts, both during and potentially even outside education emergencies. Even before the COVID-19 pandemic, there was high demand for cost-effective education programmes to help address the global learning crisis. Our results suggest phone-based targeted tutoring programmes, such as the one tested in this study, have the potential to deliver cost-effective learning gains across contexts and with governments.

We also contribute new evidence on the robustness of a cost-effective approach to measure learning—high-frequency phone call assessments. We leverage data across five countries and a series of randomized validity tests. Extended Data Table 5 shows the results of five checks we conducted, described in more depth in the Methods section. Results suggest that measurement of learning by phone can be highly robust, in addition to being cost-effective.

## Conclusion

Here we present results from large-scale randomized trials evaluating the provision of education in emergency programmes across five countries: India, Kenya, Nepal, the Philippines and Uganda. We test multiple scalable models, including government delivery, of remote instruction for primary school children during COVID-19, which disrupted education for more than 1 billion schoolchildren worldwide, as well as during an additional emergency—a typhoon—which further disrupted schooling.

Despite heterogeneous contexts, the results show that the effectiveness of phone call tutorials can scale across contexts; we find consistently large and robust effect sizes on learning, with average effects of 0.30–0.35 s.d. In the subset of trials in which we randomized whether the intervention was provided by NGO instructors or government teachers, we find large effects for both delivery models, indicating effectiveness when delivered within government systems (Supplementary Fig. A1).

These results have relevance to global efforts to support education in emergencies including conflict, disease, natural disasters and climate shocks, which routinely shut schools down. During these shocks, alternative models are needed to deliver education. The results presented here identify approaches that can scale effectively during disruptions across contexts and cost-effectively improve learning for students. This study further demonstrates that rigorous testing of programmes in humanitarian settings is possible.

Even outside education emergencies, millions of children worldwide learn very little in school, either because they are taught curricula beyond their learning level, or because they are unable to access quality instruction since they live in remote areas. Given widespread mobile phone ownership rates globally, phone-based tutoring programmes such as the one studied here have the potential to maintain schooling continuity and accelerate learning even outside emergency settings or after the initial emergency has eased. The low cost, high access and ease of implementation of mobile phone tutoring could build more resilience into education systems, enabling systems to better withstand frequent shocks, and to more generally utilize cost-effective approaches to address a persistent global learning crisis. One high profile example includes Karnataka, India, which is scaling this approach—now called ConnectEd globally and referred to in Karnataka as Ganitha Ganaka—during school and to prepare for school disruptions due to monsoon seasons. Another example includes follow-on trials that built on this study in Latin America^54^.

**Extended Data Fig. 5:**
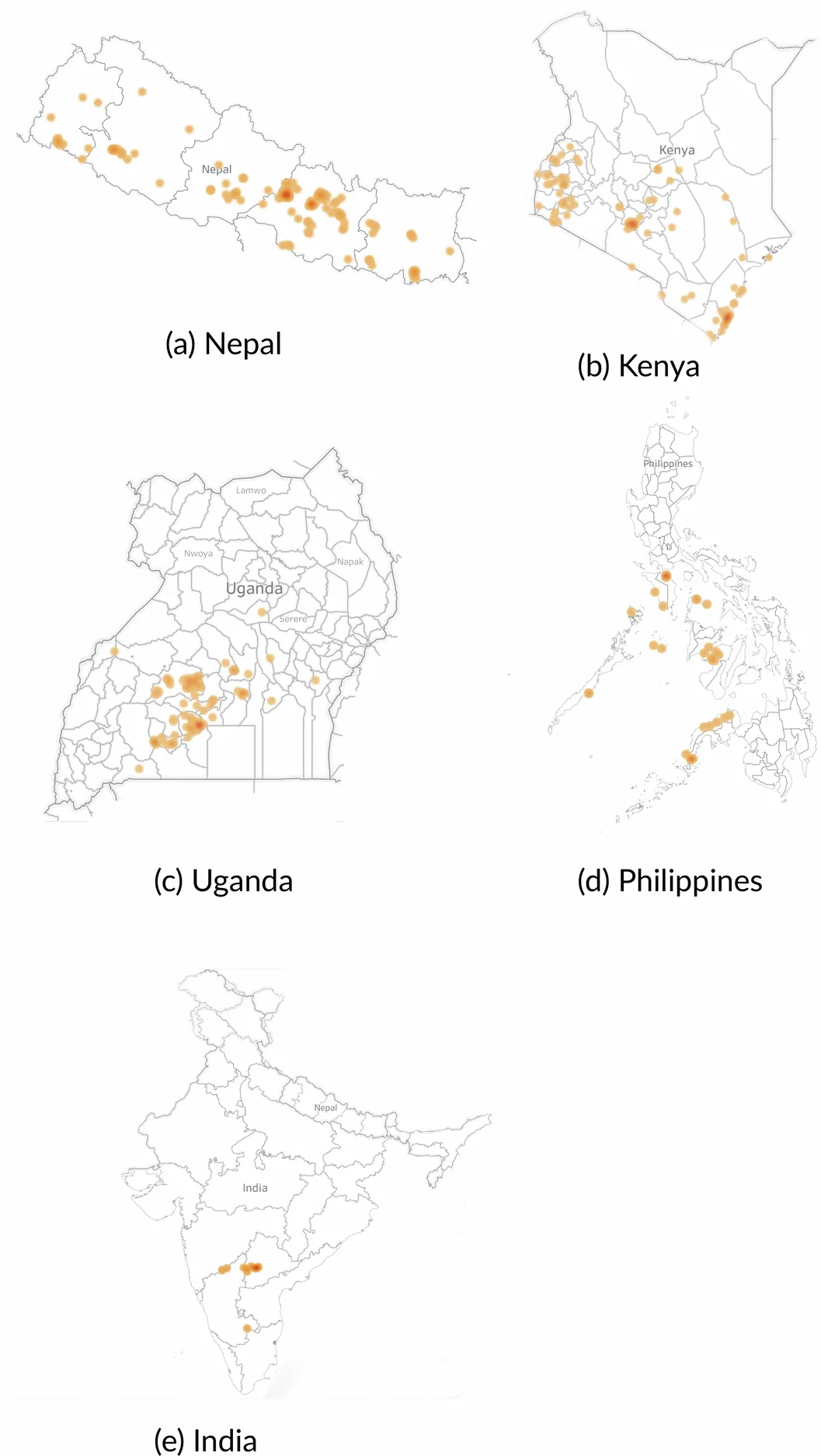
Maps of the Study Sample by Country.

This study has substantial policy implications. Multiple efforts to scale the intervention studied in this paper are underway worldwide. This paper provides critical evidence to inform effectiveness across settings and delivery models. Future research could examine effects at even larger scale. As Extended Data Fig. 5 shows, the trials covered substantial portions of the geographies studied. However, there is still value in exploring effects at national scale in future work. Future research could examine refinements to the delivery model to engage parents further^55^ and could evaluate potential spillovers on teacher instruction and student learning in the classroom. Moreover, additional work could explore long-term benefits following observed gains during the study period, with learning potentially compounding over time as skills beget more skills.

## Methods

### Data

In each country, we have two waves of data: baseline and endline. In Nepal, we conducted a baseline survey with a random 50% of the sample, and in Kenya, we relied on administrative data instead of baseline survey data. The endline surveys took approximately 30 min to administer and included approximately 20 questions. These questions included a learning assessment, child wellbeing, parental engagement in educational activities, and parental perceptions of their child’s learning. A portion of the survey was conducted with the parent, and learning outcomes were collected by directly assessing the child over the phone. Endline surveys were conducted a few months after the programme ended. A set of common core questions in the baseline and endline surveys are included in the Supplementary Information.

The learning assessment was adapted from the ASER (Annual Status of Education Report) test, which has been used frequently in the literature to measure learning outcomes^14,15^ and is used routinely across 14 countries. The test consists of multiple numeracy items, including two-digit addition, subtraction, multiplication and division problems. In addition, we asked students to solve a word problem and a fraction problem to capture learning outcomes beyond a core set of mathematical operations. Results are presented in several main results figures and tables. Distributions of raw baseline and endline learning levels are shown in Supplementary Fig. A3.

To maximize the reliability of the phone-based assessment, we introduced a series of quality-assurance measures. To minimize family members in the household assisting the child, students had a time cap of 2 minutes per question and we asked each child to explain their work. We only marked a problem correct if the child correctly explained how they solved the problem and enumerators were confident parents were not assisting their child. We also conducted a battery of validity checks to ensure the reliability of the learning outcomes. These results contribute new evidence on the robustness of remote learning assessment data across five countries. Phone assessment has emerged as a common strategy for large-scale household surveys such as the World Bank Living Standards Measurement Survey (LSMS). A growing literature has started to explore the validity of phone-based assessments to measure learning outcomes^20,56–58^, with emerging evidence suggesting phone assessment can capture meaningful information at high frequency and low cost.

Extended Data Table 5 shows the results of five checks we conducted on the validity of our main learning outcomes of learning assessments via phone. Column 1 shows the first robustness check where we compare in-person to phone-based assessment for a representative sample of the same students in Kenya. An additional test included back-checks, with a random subset of students tested twice on the same competencies. We find a strong relationship as expected, with large positive coefficients and *t*-statistics ranging from 5 to more than 20. We further randomize students to receive different problems of the same proficiency (for example, four different questions to measure two-digit addition with carryover). Finally, column 5 shows results from a real-effort question to disentangle effects of the intervention on effort on the test versus cognitive skills. Students were asked to answer several effort tasks, for example figuring out the day of the week or counting zeros and ones. Altogether, results suggest measuring learning by phone can be robust, similar to findings from the World Bank LSMS for household measures of consumption.

The survey also included questions on caregiver engagement in their child’s education and beliefs. We measured engagement by asking caregivers how often they spent helping their child with their schoolwork over the previous weeks. We also included a measure of a caregiver’s confidence in their child having made progress in learning over the previous months, and their perception of their child’s numeracy level. Additional questions included information on whether the caregiver has returned to work. We also asked about parents’ demand for remote learning services in the future, and whether they would be willing to pay for such a programme. For students, we asked about child’s mental wellbeing, how much they enjoy school, and the child’s own belief about what mathematics problems they will be able to answer. We also measured non-cognitive skills, such as perseverance and ambition. Results are shown in Supplementary Tables A2 and A3. Finally, we included demographic questions, recording the child’s age, grade and gender.

The overall sample size, pooling all sites, is 16,936 households. The flow of participants from enrolment through randomization to endline follow-up is summarized in a CONSORT diagram (Supplementary Fig. A5). For endline surveys, we randomly sampled households from the full sample to interview for a total sample of 12,331. This was due to time and cost constraints. In Supplementary Table A8, we show that those randomly selected for endline interview are statistically equivalent to the full sample at baseline along a series of indicators such as gender, student grade and baseline learning level.

Supplementary Table A9 presents the response rate to the endline surveys and an analysis of survey attrition for those randomly selected to be part of the endline sample. The follow-up rate was around 76% of respondents at endline. Supplementary Table A9 also presents a test of whether response rates differed by treatment assignment. This provides evidence that our sample has a high and unbiased response rate.

Finally, we also include a survey for teachers to assess their beliefs and instructional practices. These questions include their desire to be a teacher, and teachers’ view that the phone call tutorial programme was helpful for student learning. Questions also include instructional practices, such as involving parents in education further and better targeting feedback to students’ actual learning level. These questions can capture potentially persistent effects on educational systems through teachers changing their beliefs and behaviours beyond the lifecycle of the programme.

### Experimental design

We ran randomized controlled trials across five countries. Every trial had a control group and a combined phone call and SMS treatment arm. All studies except the trial in India also included an SMS-only treatment arm. In two countries, Nepal and the Philippines, we further randomized delivery of the phone call by NGO instructors or government teachers to assess scalability within government systems. We exploit random assignment to identify causal effects and estimate the impact of these education in emergencies interventions and various scalable delivery models.

We first estimate intent-to-treat effects per country *c* as follows:

1 $${Y}_{i,j,c}=\alpha +{\beta }_{1}{\mathrm{PhoneCalls}}_{j,c}+{\beta }_{2}{\mathrm{SMS}}_{j,c}+\gamma {X}_{j,c}+{\delta }_{s}+{\varepsilon }_{i,j,c}$$

where *Y*_*i*__,__*j*__,__*c*_ is a learning outcome for individual *i* in household *j* in country *c*. PhoneCalls_*j*,*c*_ and SMS_*j*,*c*_ take on the value 1 in their respective treatment arms and 0 otherwise. *X*_*j*__,__*c*_ denotes a vector of baseline control variables, applied consistently across trials to enhance statistical power and precision: student grade, student gender and baseline learning level. For baseline learning, we have the same assessment as the endline in all countries except Kenya, where we use administrative exam scores, and in Nepal, where we use parent perceptions of baseline learning. Since random subsamples are drawn at baseline in a few cases, we complete the sample with average estimates to enable analysis to consider the full sample at endline when including controls. *δ*_*s*_ denotes the geographic strata fixed effects—the randomization stratum relevant to each country (for example, region, district or constituency). We assess impact on operations gained as well as the highest proficiency achieved in a given country. We estimate the regression using ordinary least squares. Our main specifications are conducted per country. Given the speed and timing of the studies conducted during a historic crisis, rather than register new analysis plans, we rely on specifications published in prior related studies to guide our analysis^20^. Our specifications are straightforward, with one main learning outcome measure. In the extended data, we conduct corollary analysis where we pool across studies and we include country fixed effects *η*_*s*_. We run additional regressions using alternative weights by country as well as country-by-country estimations. Our primary analysis focuses on learning outcomes expressed as operation levels gained in raw units as well as the proportion of students who make it to the top proficiency level. We analyse standardized learning outcomes only as a corollary analysis (standardized relative to control group standard deviations at endline and centred at mean zero). All tests are two-sided statistical tests. All analysis was conducted in Stata/MP 18 software.

We also ran a subset of trials in which phone calls were delivered by NGOs or government teachers. We estimate government (Gov) versus NGO delivery effects per country using the following specification:

2 $${Y}_{i,j,c}=\alpha +{\beta }_{1}{{\rm{P}}{\rm{h}}{\rm{o}}{\rm{n}}{\rm{e}}{\rm{C}}{\rm{a}}{\rm{l}}{\rm{l}}{\rm{s}}}_{j,c,{\rm{N}}{\rm{G}}{\rm{O}}}+{\beta }_{2}{{\rm{P}}{\rm{h}}{\rm{o}}{\rm{n}}{\rm{e}}{\rm{C}}{\rm{a}}{\rm{l}}{\rm{l}}{\rm{s}}}_{j,c,{\rm{G}}{\rm{o}}{\rm{v}}}+\gamma {X}_{j,c}+{\delta }_{s}+{\varepsilon }_{i,j,c}$$

All standard errors are clustered at the level of the unit of randomization. In most countries this was the household level, which was de facto the same as the student level since one student participated per household; in a subset of treatment comparisons it was the school grade level. In India, Nepal and Uganda all randomization was at the household level. In the Philippines, the NGO arm was randomized at the household level; the government arm was randomized at the school grade level, with a corresponding control group randomized at the same level for each arm. In Kenya, the phone call arm was randomized at the school grade level whereas the SMS arms were cross randomized at the household level. Of note, robustness checks find little to no difference if standard errors are clustered or not across all specifications. This is largely due to the fact that there is substantial variation in learning outcomes within a cluster, such that the intracluster correlation is very low at about 0.03 in the Philippines and Kenya. The above specifications are the core estimation strategies. Additional specifications include other outcomes of interest beyond learning, such as engagement, as well as heterogeneity analysis, and various types of pooling across countries.

Due to randomization, we expect treatment and control groups to be balanced at baseline in expectation. Supplementary Table A10 presents balance tests using baseline data for key demographics and the same learning level variable. We find no statistically significant differences across multiple dimensions, including gender, grade, caregiver type, caregiver education levels and baseline learning. This is robust to multiple specifications, including with and without country fixed effects. Similar results are found for each country, with balanced samples in each individual country. This reveals that each randomization yielded balanced arms in line with expectations.

In all sites, the experimental design had high compliance rates; nearly all randomized treatment households participated in the expected treatment group. Detailed weekly monitoring data shows high take-up and fidelity, as shown in Extended Data Fig. 1.

In a secondary analysis, we embed another randomized trial which randomly allocated a subset of government teachers to deliver the phone call tutorials out of a pool of hundreds of eligible teachers in Nepal. Supplementary Table A6 shows balance tests with no statistically significant differences among treatment groups. We assess whether participating in phone call tutorials changed teachers’ subsequent beliefs and behaviours, which we estimate for teachers *i* at the school level *j* in each country *c* as follows:

3 $${S}_{i,j,c}=\alpha +{\beta }_{1}{\mathrm{TeacherCalls}}_{i,c}+\gamma {X}_{i,c}+{\delta }_{s}+{\varepsilon }_{i,j,c}$$

where S_*i*,*j*,*c*_ captures teacher beliefs or instructional practices. These outcomes measure potential spillovers into the education system through teacher beliefs and behaviours which could persist beyond the programme.

### Ethics statement

Insitutional review board (IRB) approval was granted by the Health Media Lab IRB (889OXFD21) and Columbia University IRB (21-384). Informed consent was performed in accordance with all relevant guidelines and regulations and obtained from caregivers, child assent and government approvals in each country. An additional country-specific IRB approval was granted by Nepal’s Health Research Council (776/2020) due to local regulations. The trials were registered in the AEA registry: AEARCTR-0009779. After the study period, control group students were later able to be enroled in the intervention to benefit from the programme.

### Reporting summary

Further information on research design is available in the Nature Portfolio Reporting Summary linked to this article.

## Online content

Any methods, additional references, Nature Portfolio reporting summaries, source data, extended data, supplementary information, acknowledgements, peer review information; details of author contributions and competing interests; and statements of data and code availability are available at https://doi.org/10.1038/s41586-026-10990-x.

## Supplementary information

Supplementary Information This file contains Supplementary Methods (trial design, country contexts, intervention components and survey instruments), Supplementary Discussion (typhoon results, parent and child beliefs, non-cognitive skills, potential crowd-out on employment, and robustness), 10 Supplementary Tables, 5 Supplementary Figs., and the education-in-emergencies school-closure source list.

Reporting Summary

Peer Review File

## Figures and Tables

**Extended Data Table 1:**
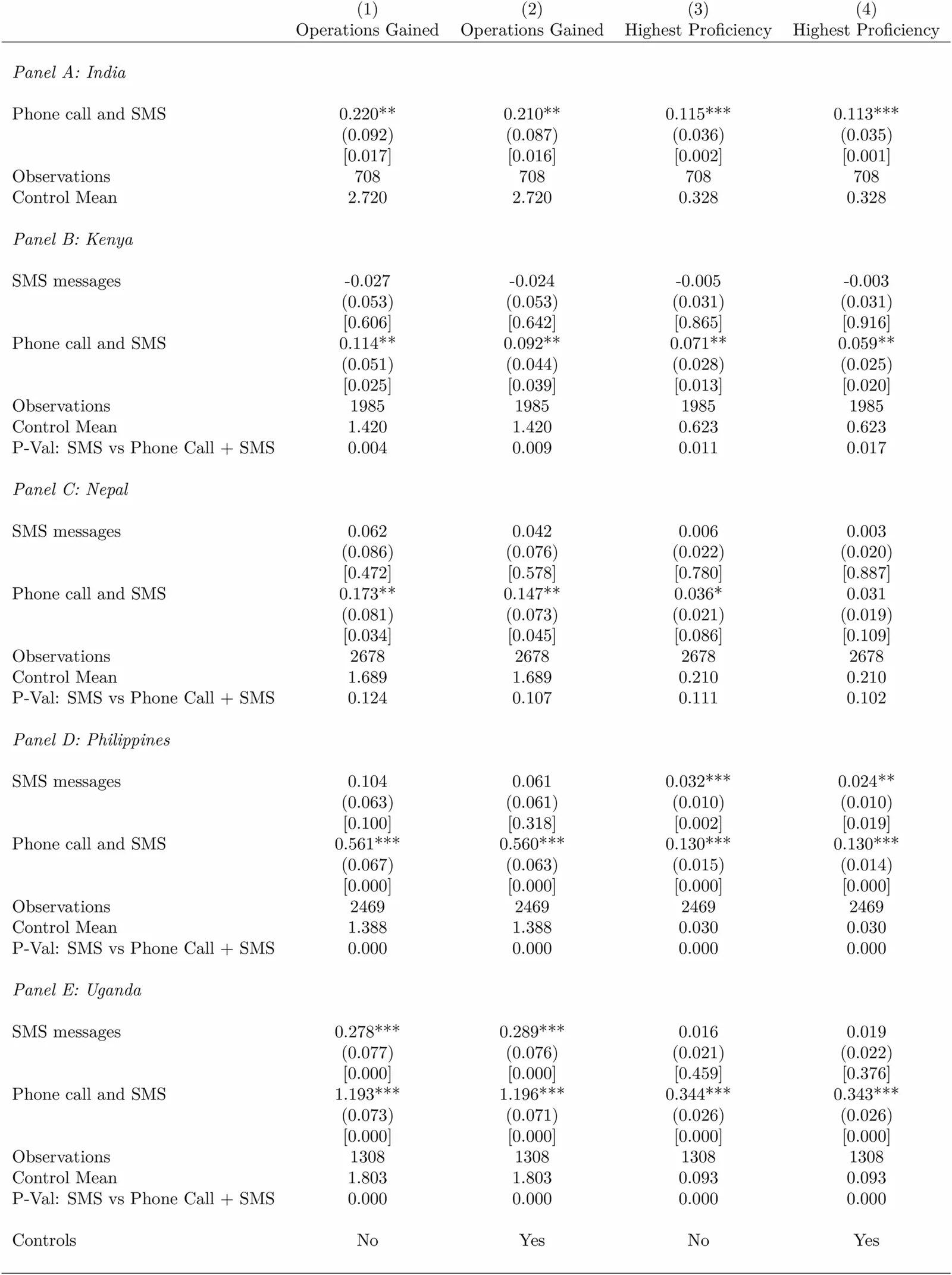
Learning Outcomes Gained, By Country

**Notes:** This table reports treatment effects on learning outcomes by country. Operations gained refers to the number of operations gained, with each operation scored as an additional level gain (e.g. beginner = 0, addition = 1, subtraction = 2, multiplication = 3, and division = 4). Highest proficiency refers to the highest level taught and tested in a given country, which is 2-digit division in all countries but Kenya where students were younger and taught up to 2-digit subtraction. Controls include student grade, gender, geographical strata, and baseline learning level. Standard errors are in parentheses and p-values are in square brackets.

**Extended Data Table 2:**
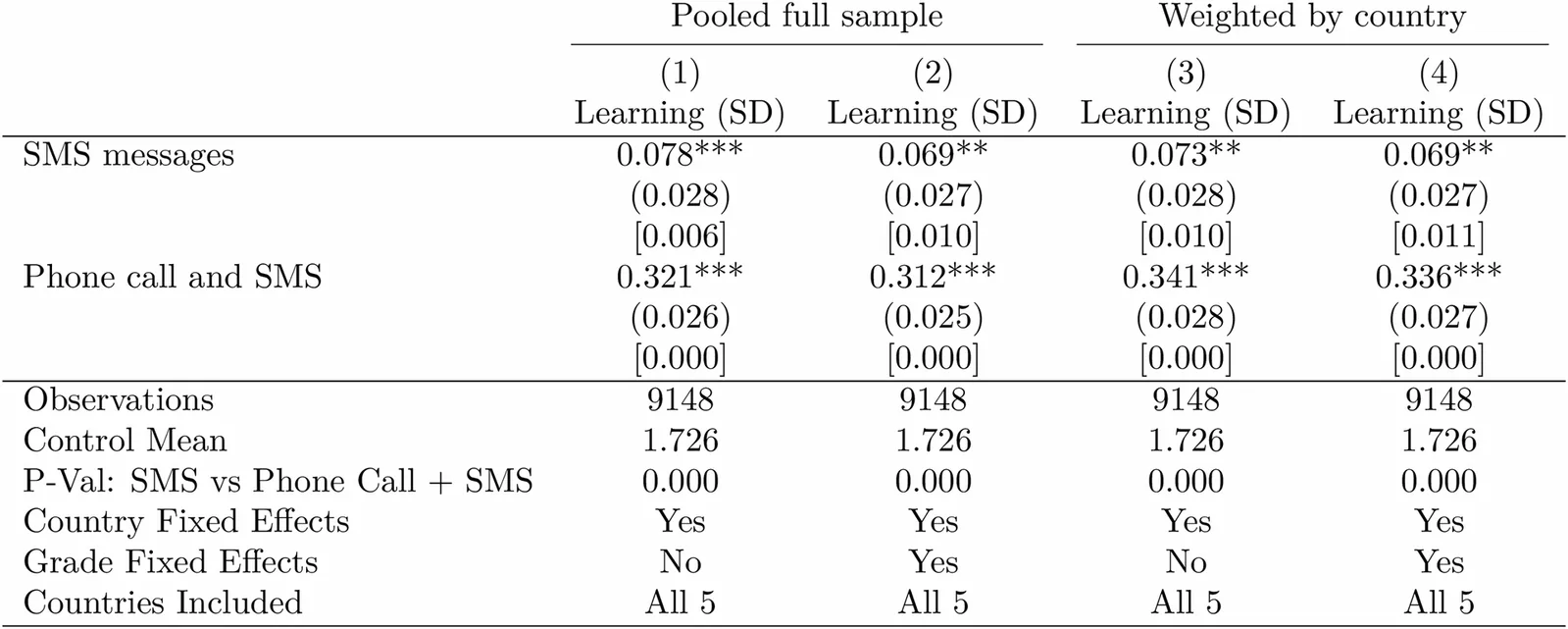
Learning Outcomes at Scale Across 5 Countries

**Notes:** This table reports treatment effects on learning outcomes for a pooled sample as well as samples weighted by country-arm. Learning refers to how a child scores on four basic numeracy options: no operations correct, addition, subtraction, multiplication, and division (measured on a scale of 0–4 and expressed in terms of standard deviations, standardized using the control group mean and standard deviation at endline). “Pooled full sample” gives equal weight to each observation; consequently, country-treatment arms with larger samples contribute more to the pooled estimate. “Weighted by country-arm” reweights observations by country so that each observed country arm contributes equal total weight. Column (1) measures the learning differences between the treatment and control group, measured in standard deviations, with country fixed effects only. Column (2) includes baseline controls and country and grade fixed effects. Column (3) and column (4) weigh the results by country-arm. Standard errors are in parentheses and p-values are in square brackets. Given learning outcomes are standardized to have a control mean of zero, we include in the control mean row a more intuitive comparison. We include unstandardized average student learning level in the control group at endline. This variable takes the value of 0 for students who got zero operations correct, 1 if students got addition correct, 2 if subtraction was correct, 3 if multiplication was correct, and 4 if students got division correct. For example, in this table, average student levels in the control group at endline were 1.726, which is the equivalent of a student knowing between addition and subtraction level.

**Extended Data Table 3:**
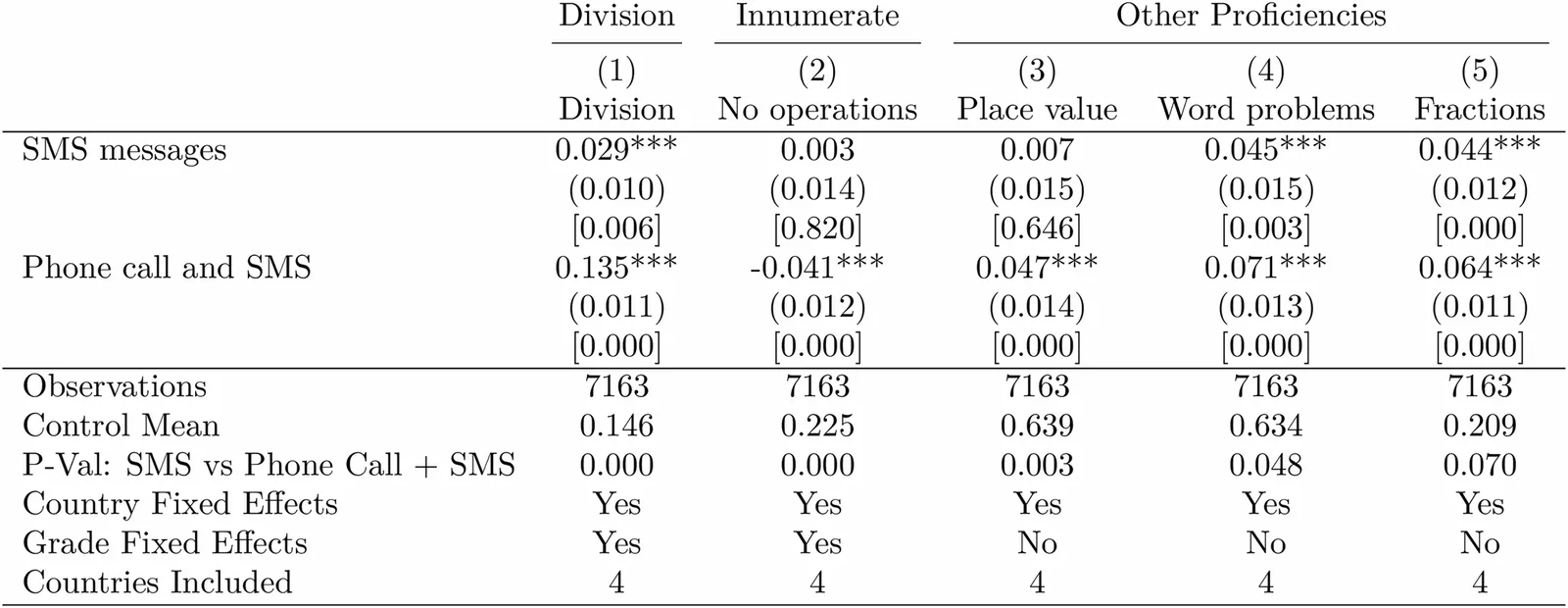
Learning Outcomes by Multiple Proficiencies

**Notes:** This table reports learning outcomes across different proficiencies. Column (1) highlights the learning gains in terms of share of students who learned division. Column (2) shows the share of students who could not correctly answer any of the basic numeracy operations (referred to as innumerate). Columns (3), (4), and (5) show the share of students who could correctly answer place-value problems, word problems, and fractions, respectively. The place-value and higher-order questions were not asked of the Grade 1 and 2 students in Kenya as these were not covered in their standard school curriculum at these ages. Standard errors are in parentheses and p-values are in square brackets.

**Extended Data Table 4:**
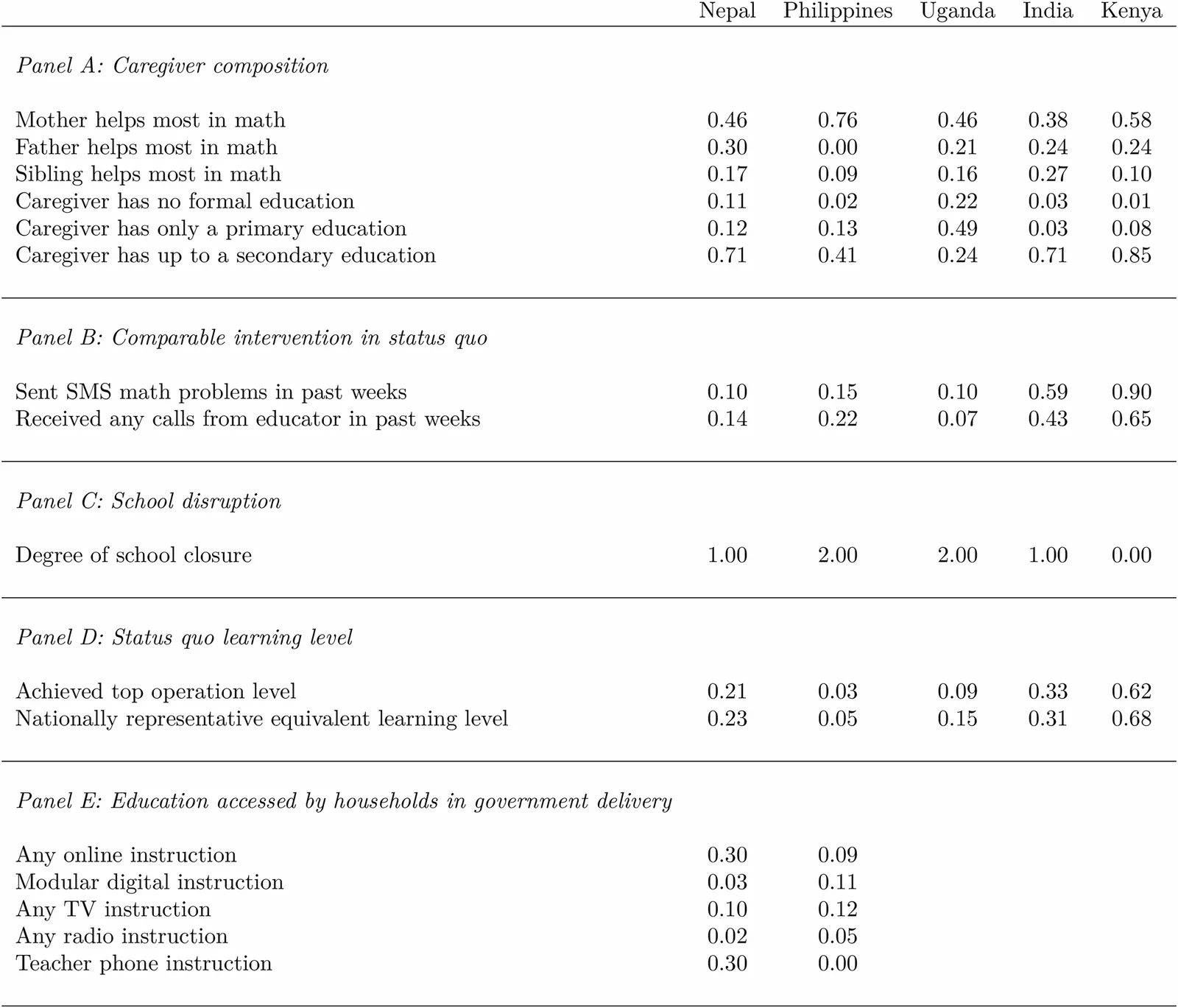
Counterfactual condition

**Notes:** This table describes status quo conditions in controls groups. Means are reported on binary variables for nearly all variables, interpreted as percents; the only exception is the degree of school closure which is coded as 0 for mostly open schools during the study, 1 for partially closed school during the study; and 2 for fully closed schools during the study period. The top learning level is simple division in most countries; Kenya is subtraction since enrolled students were younger. The nationally representative equivalent is drawn from national assessments conducted in Nepal, Uganda, and Kenya using the ICAN comparable assessment which the ASER tool and our assessment directly maps onto, and from ASER in India. The Philippines data is drawn from the RMA data for the top level of proficiency.

**Extended Data Table 5:**
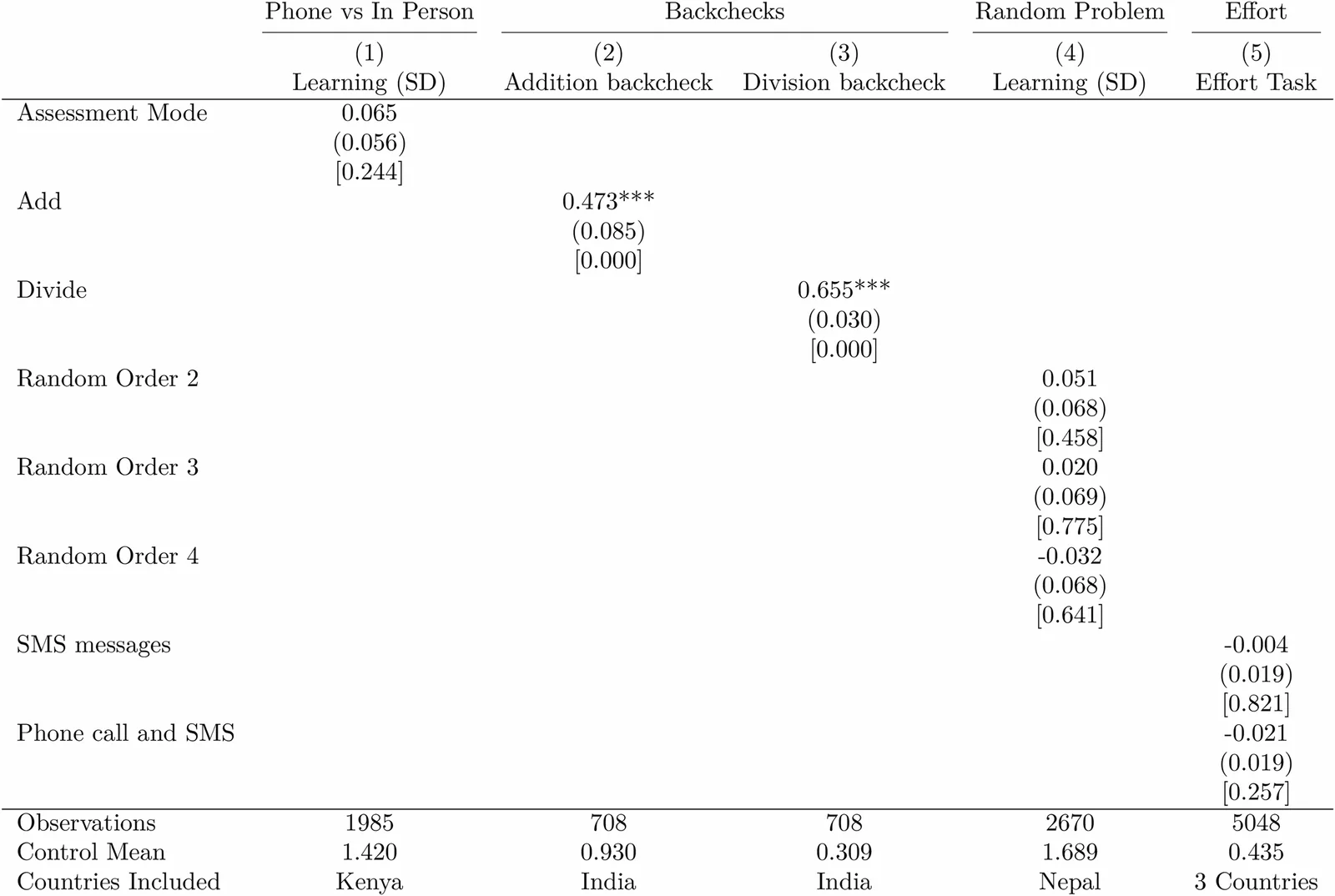
Robustness Checks on Learning Outcomes

**Notes:** This table reports the robustness checks on learning outcome measurement. Learning refers to how a child scores on four basic numeracy options: no operations correct, addition, subtraction, multiplication, and division (measured on a scale of 0–4 and expressed in terms of standard deviations, standardized using the control group mean and standard deviation at endline). Column (1) shows the results on average student level for assessments via phone relative to the same assessment in-person. Column (2) and (3) show backcheck results on addition and division. Column (4) shows results of randomizing students to receive different problems of the same proficiency. Column (5) shows results from effort questions. Standard errors are in parentheses and p-values are in square brackets. In column 1, given learning outcomes are standardized to have a control mean of zero, we include in the control mean row a more intuitive comparison. We include unstandardized average student learning level in the control group at endline. This variable takes the value of 0 for students who got zero operations correct, 1 if students got addition correct, 2 if subtraction was correct, 3 if multiplication was correct and 4 if students got division correct.

## Data Availability

Anonymized replication data are available at https://github.com/nangrist/mobile-education.

## Code availability

All replication code is available from https://github.com/nangrist/mobile-education.
