# Supplementary material for: Mobile education builds resilience during shocks in five countries
Source: Nature. 2026 Sep 9;657(8132):689–96. doi: 10.1038/s41586-026-10990-x (PMC13581608; doi:10.1038/s41586-026-10990-x)
Supplement: Supplementary file 1 [file 41586_2026_10990_MOESM1_ESM.pdf]

nature portfolio

https://doi.org/10.1038/s41586-026-10990-x

Supplementary information

Mobile education builds resilience during shocks in five countries

In the format provided by the authors and unedited

Supplementary Information

Mobile education builds resilience during shocks in five countries

Supplementary Discussion

A. Results from Another Education Emergency

COVID-19 disrupted education for over a billion children, causing one of the world's largest scale education emergencies. However, as Figure 1 shows, many other education emergencies occur beyond COVID-19. Yet little experimental evidence exists in these settings on how to most effectively promote learning.

In addition to the core set of randomized trials assessing impact of phone-based tutorials during COVID-19 school disruptions, an additional education emergency took place during the course of the study – a devastating typhoon in the Philippines which destroyed 4,000 classrooms and disrupted learning for 2 million children. We collected detailed student-level data on who was affected by the typhoon (called "Typhoon Rai"). The typhoon resulted in a shock which further disrupted schooling and learning. Our initial randomization remained unbiased among groups affected by the typhoon, enabling us to assess effectiveness of phone call tutorials in this additional education emergency context.[1] The results provide evidence on the disruptive effects of the typhoon on learning and approaches to ensure learning continuity during disruptions.

We start by estimating learning losses due to Typhoon Rai. Results in Table A1 show that the typhoon was associated with reductions in learning by approximately 0.13–0.21 standard deviations (Column 1 and 2). In addition, since randomization of treatments is orthogonal to which students were affected by the typhoon, we can estimate the impact of the phone-based tutorials during this additional emergency. Results in column (4) which includes controls show that despite the detrimental effects of the typhoon, phone call tutorials continued to be effective, with 0.22 standard deviation learning gains relative to the control group ($P < 0.05$). SMS messages alone did not generate detectable gains in learning. These results reveal that the results of effective education in emergencies programs, such as phone call tutorials, can persist across multiple emergency settings.[2]

B. Additional Impacts

We examine impacts on beliefs about learning. We provide new evidence on children's perceptions of their own learning in Table A2 Column 1, an outcome rarely explored to date. We find children update their beliefs substantially, by 0.21 standard deviations in the phone call and SMS treatment and by 0.04 in the SMS-only treatment – corresponding to patterns in learning gains observed in Table E2. Column 2 examines caregivers' beliefs and shows that they observed their child's

[1] Randomization to treatment groups and being affected by the typhoon has p-values of 0.65 and 0.62 for the Phone Call as well as the SMS only treatment, respectively.

[2] Implementation data suggests that households that were exposed to the typhoon had similar levels of program engagement throughout, and also had high endline survey response rates similar to other studies. For example, typhoon-affected students attended only slightly fewer sessions than non-typhoon affected households (7.39 vs 7.45), and only marginally less time spent per session (20.4 minutes vs. 21 minutes in the final week). This highlights the resilience of the mobile phone program platform to different disaster types, such that even in the midst of extreme weather disruption, households were responding to tutoring and endline calls at high rates, similar to those in other studies and in non-disaster-affected areas.

learning, updating their beliefs about their child's level of learning by 0.114 standard deviations in the phone call and SMS treatment and by 0.075 in the SMS only treatment – corresponding broadly to gains observed in Table E2. This reveals parents can learn through noticing [1] although imperfectly and less accurately than their children. Moreover, caregivers explicitly state that they think their child's learning has progressed (column 3), with large effects for phone call tutorials, in line with where treatment effects are largest. These results build on a literature exploring parents' knowledge of their child's learning level, enabling them to better support their education [2].

Finally, Table A3 examines impacts on non-cognitive skills. A growing literature highlights the importance of both cognitive as well as non-cognitive skills for future life outcomes, such as graduation from college and labor market outcomes [3]. We assess impacts on a series of non-cognitive skills, such as perseverance and ambition [4]. We asked students who had just completed a riddle if they wanted to complete another difficult riddle question (perseverance), and if so, whether they wanted an easier or harder one (ambition). We find sizable effects on these outcomes, with 6.1 percentage point gains in ambition, a 27 percent increase relative to the control mean. We further find positive effects on measures of child well-being, such as enjoying school and worrying less, statistically significant at the 90 percent level and above. There were limited effects on these non-cognitive skill outcomes for the SMS-only treatment.

These results reveal that while there is often a debate between whether education should focus on cognitive or non-cognitive skill acquisition, they are not mutually exclusive. Indeed, some educational interventions, such as the phone call tutorials tested in this study, can promote both. It also shows that phone-based tutoring models can impart the types of non-cognitive skills that are often viewed as a benefit of brick-and-mortar schools. This suggests that when emergencies do disrupt education, children can still make progress on both cognitive and non-cognitive skills from targeted phone tutoring programs.

C. Potential for Crowd-out

We explore the potential for crowdout. In Table A4, we show results on caregivers' time usage, including on overall educational investments and time spent at work. It is possible that by inducing caregivers to support one child's education, they invest less in other children in the household's education. Yet we find evidence of the program crowding in, rather than crowding out, overall time spent on education. The program caused a net increase in the share and frequency of caregivers undertaking educational activities with their children. Moreover, some studies suggest that mothers' engagement in their child's education could crowd out their labor market participation [5]. However, we find limited evidence of such a trade-off, potentially since the program is highly efficient, requiring only a short amount of time to produce large learning gains. These results suggest that highly efficient education programs can deliver large learning gains, with minimal risk of crowd-out of other productive educational and work activities.

D. Additional Robustness Checks

We include an additional robustness check correcting for multiple hypothesis testing in Table A5. We include sharpened q-values to adjust for multiple hypothesis testing [6] to address the False Discovery Rate (FDR). Estimates remain highly statistically significant.

E. Context

A Global Learning Crisis, Exacerbated by COVID-19

While schooling rates have increased worldwide, students face a global learning crisis, with many students in school but learning very little [7]. In our baseline survey, for example, 36 percent of students in grades 3 to 5 could not do any basic operations, falling well behind curriculum expectations. COVID-19 school closures exacerbated this global learning crisis, with school closures forcing over 1.6 billion learners out of classrooms. These types of school closures result in large learning losses which have been documented in North America, Western Europe, Asia, and Sub-Saharan Africa. The lasting economic toll of learning loss is enormous, estimated at over 20 trillion dollars [8]. This pre-existing and exacerbated learning crisis necessitates effective educational approaches that can rapidly improve learning outcomes during and beyond emergencies.

Education in Emergencies

COVID-19 school closures are a recent large-scale example of disruptions to schooling. However, emergencies that affect education are all too common, and affect many people every year. We present novel data documenting the extent of school closures over the past two decades for a subset of countries. We codify information from various sources, including reports from international aid organizations such as Save the Children and UNICEF, the United Nations Office for the Coordination of Humanitarian Affairs (OCHA), as well as national and international news reports.

Figure 1 plots an index of the length of school closures and number of people affected by shocks that have disrupted schooling by country and year. We observe that large-scale disasters affect many people, occur in many countries, and happen at high frequency. The spread of multiple diseases has shuttered schools, including Swine Flu in China in 2009, Foot and Mouth in Cambodia in 2012, and Ebola in Sierra Leone in 2014. In 2019, smog and air pollution from wildfires closed schools around Mexico City. In 2020, a cyclone in Vanuatu destroyed and damaged 885 schools. Extreme climate events often disrupt schooling, such as water shortages in Venezuela in 2017. Floods and rainy seasons perhaps most routinely cause widespread school disruption. For example, in 2017, monsoons and flooding closed and damaged over 10,000 schools across Malaysia, India, Bangladesh and Nepal. In Pakistan, nearly 8,000 schools were damaged in 2010. A more recent crisis in Pakistan in 2023, which will be included in an extension of this dataset, disrupted 34,000 schools. As climate change-related shocks increase, school disruptions are likely to occur with ever greater frequency and consequence, stunting students' education.

In recognition of the importance of addressing shocks to education, the United Nations established a billion-dollar Global Fund called "Education Cannot Wait" in 2016 at the World Humanitarian Summit to support learning in emergencies and protracted crises. Multiple additional international aid structures also exist to support education in emergencies. For example, the Inter-agency Network for Education in Emergencies (INEE) was established to connect and share best practices amongst more than 18,000 members from 4,000 institutions globally working in education in emergencies, including the United Nations, governments, NGOs, teachers, and students.

A prominent review on education in emergencies notes that most evaluations in the sector have been qualitative [9]. The review highlights a major gap in experimental studies and the need for interventions which focus on improving education quality, in addition to education access, in emergency settings. They identify the use of mobile phone technology to deliver educational instruction as a particularly promising yet underutilized approach, and recommend future experimental research in this area. This study aims to address these critical research gaps, contributing experimental evidence on education in emergency programs which can promote learning cost-effectively,

across settings, and with governments.

F. Study Overview of Randomized Trials Across Five Countries

Our evaluation of education in emergencies interventions took place in the context of COVID-19 school closures across five countries: India, Kenya, Nepal, the Philippines, and Uganda. In total, over 16,000 households were enrolled in this multi-country evaluation. In each household, a primary school student and a caregiver were identified to receive the intervention. We asked households to nominate the main caregiver who provides educational support to their child: 53.3 percent of nominated caregivers were mothers, 17.4 percent were fathers, 17.3 percent were siblings, and 3.2 percent were grandparents. 24 percent of nominated caregivers had completed primary education or less. Students were enrolled in grades 3 to 5, with one exception in Kenya where students were in grades 1 and 2. The trials took place between December 2020 and July 2022. Table 4 includes a summary of key facts per country trial, and Figure A2 shows a detailed timeline. Figure E5 maps the location of the children's schools within study countries; it shows study participants are distributed across wide areas in most countries, and in some cases, are relatively nationally representative. Students provided phone numbers where they could be contacted, which was most often their parent's phone number, and in some rare cases, the phone of a neighbor or other community member.

Our evaluation includes multiple education in emergencies programs and delivery models. We leverage mobile phones – a high-access, low cost and scalable way to reach students and their caregivers when school is out of session – to provide various educational interventions over the course of eight weeks.[3] One treatment includes a set of SMS messages, such as numeracy content provided weekly, as well as nudges to engage in educational activity. A second treatment added weekly one-on-one 20-minute phone call tutorials. Phone calls covered foundational numeracy and aimed to target instruction to student learning levels via light-touch, high-frequency assessments conducted on a weekly basis. Using the assessment, instructors led students through foundational numeracy practice problems to help them master addition, subtraction, multiplication, and division. For example, students who did not know addition would be taught addition, while students who knew addition but not subtraction would be taught subtraction. Calls were made to caregivers who invited the child in their household to join the program, and would then hand them the phone or use speakerphone for the tutorial session. The implementation ratio of students to teachers was approximately 20:1 for teachers implementing the program full-time, and 5:1 for teachers implementing the program part-time. The phone call program is called "ConnectEd", highlighting phone calls' ability to connect students to quality education even during school disruption.

In addition, we tested several scalable delivery models, including NGO and government teacher delivery, which were randomized within a country. The government delivery arms were conducted with government teachers. They included headquarter ministry sign off, as well as regional government engagement, including joining training, engagement with routine monitoring data, and periodic mid and high-level steering committee meetings. Some delivery models hired teacher aides to deliver instruction, while others used teachers.[4]

We include a few descriptive statistics to describe our samples. In our five-country sample, the

[3] These types of mobile phone-based interventions have shown to be able to close parent-child information frictions and improve learning in high-income settings [2] as well as low and middle-income settings [10].

[4] Note: we use the terms 'teacher' and 'teacher aide' to reflect several types of trained teaching instructors. Those referred to as 'teachers' are currently employed primary school teachers. 'Teacher aides' include community education volunteers as well as instructors trained to be teachers but currently employed by NGOs. Details of implementer types are discussed in each country section below.

median student is in grade 3 and 51 percent of students are female. On baseline student learning proficiency, 36 percent of students did not know any basic operations, 27 percent were at addition level (meaning they had 'mastered' addition and were being taught the next level), 12 percent were at subtraction level, 17 percent at multiplication level, and 7 percent at division.[5] These low learning levels are well below grade-level curriculum expectations. Moreover, detailed monitoring data shows high week-on-week engagement across sites ranging from 70 to 80 percent, revealing the scalability and robustness of the approach even in disrupted and low-resource environments, as shown in Figure E1. We also find sustained rates of engagement across all eight weeks of phone call tutorials staying within a narrow range of high engagement.

We describe the counterfactual conditions in each context, as shown in Table E4. We find that in most settings, few educational options were accessed by households. For example, less than two thirds of students receive calls from educators in all countries in the past few weeks; in some cases, such as Uganda and the Philippines, less than 22 percent of households say they received any calls from educators in the past few weeks in the status quo. Learning levels were also lowest in the status quo in the Philippines and Uganda, with just 3 and 9 percent of students able to do simple 2-digit division in the counterfactual. The degree of school disruption was also highest in Philippines and Uganda, with full school closures during the whole study period. These learning levels were largely representative of national patterns. For example, in Uganda, 9 percent could reach the top division operation level relative to 15 percent in a national survey; in Nepal 21 percent of our sample could reach the top level relative to 23 percent in a national survey. Across all countries where we have data, our sample was within 1–6 percentage points of the national average.

This set of five trials sought to scale an approach first tested in Botswana in 2020 [10]. The original Botswana trial tested the impact of a phone based tutoring program that had been developed during the first few months of COVID-19. The NGO Youth Impact, one of the largest in the country, developed a phone call that targeted tutoring for remote settings when schools were disrupted. The Botswana study – a tightly controlled initial proof-of-concept – found the program to be cost-effective, resulting in a 0.12 standard deviation improvement in learning outcomes. The present study sought to test the scalability of the approach across five country contexts and a variety of implementer types, including scalability through government delivery models. A rich array of data was collected with response rates around 76 percent, enabling both high-quality evaluation and detailed exploration of outcomes and mechanisms.

In all contexts, the NGO that first developed the program, Youth Impact, provided support to each of the implementing partners in new countries to help train their staff in the program, as well as provide monitoring tools, training, and technical assistance. Support typically involved providing the curriculum and advice if any adaptations were needed for the local context (for example, these ranged from careful language translation to adapting material if place value was taught differently in new contexts); a several-hour training-of-trainers session; monitoring surveys and advice on how to use the monitoring data tools; and occasional meetings during implementation to give advice and troubleshoot. All interactions between the research team and implementing partners took place remotely over video or voice calls. Training of trainers was led by a Youth Impact master trainer for each local implementing partner, who in turn directly trained teachers and tutors in their context and language. Training was practical and interactive, with breakout groups to practice tutoring delivery, followed by live phone calls to practice with real households.

Once implementation began, each week, program managers in each implementing organisation collected and analysed weekly monitoring data submitted by all tutors. This allowed the program

[5] This excludes Kenya, which is the only country where we do not have baseline student level data identical to the other countries broken down by specific proficiency, though we have baseline test score data on a 100-point scale.

coordinators in each implementing organisation to monitor and follow up on the weekly phone call success rate and program delivery. Implementing organisations used this data to provide support to help teachers and tutors troubleshoot, reach more students, improve targeting accuracy, and share lessons and tips across tutors. Youth Impact supported partners across countries, providing a coordinating mechanism to share lessons across trials. However, Youth Impact had no prior presence in these countries, showcasing a replicable model for scale-up in new settings.

A typical phone call involved the following format. The tutor would call the household after arranging a mutually agreeable time. Phone calls were directed toward the student, although caregivers were encouraged to place the phone on speaker and be available to support. Working at the student's identified learning level from their previous week's performance, tutors guided students through a simple set of 3–4 steps on how they should solve the operation being taught that week (addition, subtraction, multiplication, or division). Once the student had walked through a series of problems with the tutor and they had solved a few problems together, then the call concluded with a 'checkpoint question'. This consisted of one math question at the level taught that week. This question enabled tutors to evaluate and update the child's learning level to ensure they could target instruction to the child's correct level the following week. For example, a student who could not do the week's addition checkpoint question would receive instruction in addition in the following week's call; if they got addition correct, the following week they would be taught subtraction. The content of the calls and SMSs was focused on teaching the four foundational numeracy skills of addition, subtraction, multiplication and division. This is broadly aligned to a core set of competencies expected to be taught in school in the study countries for students in grades 3 to 5. I.e. by grade 4, most curricula expect students to know two-digit division.

The weekly SMS messages included a set of math problems that students were encouraged to solve between calls. Each week, there was a problem provided for each level: i.e. an addition problem, a subtraction problem, a multiplication problem, and a division problem. A typical SMS message was very simple and looked as follows for an addition level student: "Welcome to Week 2! ADDITION: 14+46=?; 18+33=?"

Trainings were primarily conducted virtually. First, a training of trainers was conducted. Program coordinators and senior project staff from partner organisations in each country (government, NGOs, and the World Bank) took part in a country-specific practical, interactive Training of Trainers workshop. This was delivered online by staff from Youth Impact. This was followed by a multi-day training of teachers and tutors led by each country's respective implementing organisations. These training sessions were contextualized based on country context, with training delivered in the local language and with contextually relevant insights and best practices. Implementer training was delivered online in all countries except in Kenya and Uganda, where training was in-person.

During implementation, Youth Impact's master trainer and program coordinator had periodic calls with each implementing organization to help troubleshoot and share best practices learned from implementation in other countries. For example, Youth Impact reviewed data with implementing partners, shared tips about the best times to schedule sessions with households throughout the week, and shared suggestions from how other countries had paced their lessons and targeted instruction successfully.

The government-led interventions were led largely by the government. The government assigned a point person within the ministry to lead the intervention and coordinate within relevant government structures and to support teachers. Government leads had regular phone and online communication with their teachers. To the extent that there was NGO involvement, it was in conducting the training of trainers and sharing of draft tools and phone call guidelines to ensure common content and approaches were delivered across treatments. In the government implementation trial arm in Nepal, Youth Impact trained Government teachers with the support of World

Bank staff, who then supervised implementation. In the Philippines government arm, Youth Impact had trained Innovations for Poverty Action who then trained and supervised the government teachers.

Specific Country Context for Each Trial

For each country context, we describe the status quo learning levels and COVID-19 education context, the implementing partner and sample description, and the instructor type. Figure A4 characterizes school disruptions in all five countries. In each setting, learning gaps are large both before and during COVID-19, showing a need for more high-quality and scalable educational instruction. In addition, the typical government responses to school disruption, such as radio and TV, have engagement rates below 30 percent.[6] This highlights the need for remote approaches which can yield higher engagement, such as phone call tutorials. Moreover, this puts in context the significance of the high engagement we find of the phone call tutorials in our study, consistently reaching over 70 to 80 percent. Finally, the diverse settings and implementation models in the study, spanning five countries as well as NGO and government delivery, enables us to assess the scalability of targeted phone tutoring across a wide array of contexts and delivery models.

India Prior to COVID-19, India placed near the bottom of all countries taking the Program for International Student Assessment (PISA), ranking 72nd out of 73 countries. In our baseline survey, only 24 percent of the Grade 3–5 students in the sample could do division, showing a substantial need for additional foundational numeracy instruction.

During COVID-19, schooling was significantly disrupted in India. In our sample in Telangana state, primary schools were partially closed for the duration of the program [11]. In response to the crisis, the government encouraged households to access educational materials shared on TV, online, and radio.

The implementing NGO in India was Alokit, a non-governmental organisation based in Hyderabad, which is part of an international network of organizations called Global School Leaders. Alokit provides education and school leadership-related programming in schools. The program supported government efforts to ensure all primary school students achieve foundational numeracy skills.

Alokit had pre-existing relationships with government officials and study schools, nearly all of which were government residential schools. The majority of the students were from rural areas and belong to India's most marginalised caste communities. Between two to four teachers were selected from each school to tutor the students. The phone calls were delivered by school teachers based in the same schools as the students in the study. Calls were conducted in either of two languages, English or Telugu, the most common language in the state.

Kenya Although Kenyan students have higher foundational learning levels than most other countries in Sub-Saharan Africa, learning outcomes still lag far behind those in higher-income countries. In third grade, only 47 percent of Kenyan students could solve a second grade mathematics problem [12], and 36 percent of grade 2 and 3 students achieved a minimum proficiency level in mathematics [13].

[6] In all contexts, support by governments and school districts during disruption was mixed, with provision of some public education support such as television and radio programming, and encouragement of teachers to support their students remotely, though evidence suggested limited uptake of these services. In this section we outline what services were available and taken up in the countries where we have data.

By December 2020, when the study launched, schools had been fully closed for 9 months. Schools re-opened from early 2021 onwards, partway through the intervention. The main distance learning programs available to students spanned TV, radio, and online and phone-based educational offerings.

The study took place in 30 of Kenya's 47 counties, in the universe of 112 schools the partner operated. NewGlobe, the partner NGO of the Kenya study, operates one of the largest, low-cost private school networks across the country. In Kenya 33 percent of pre-primary students are enrolled in private schools, and 16 percent of primary students. Kenya was the only context where grades 1 and 2 were included in the program, rather than grades 3 through 5. This was done because the baseline level of foundational numeracy skills was higher than in other contexts, and thus earlier grades were most comparable in terms of baseline learning levels.

The phone call program was implemented by students' normal class teacher, who undertook the phone calls during school closures, as well as after school as part of their teaching duties once schools re-opened part-way through the study. Randomization was at the teacher level, equivalent to the school-grade level. Teachers were advised to deliver tutoring sessions in English, and also conducted some parent interactions in Kiswahili.

Nepal In 2018, only 28 percent of Grade 5 students in Nepal demonstrated grade-level proficiency in mathematics [14]. In our baseline survey, only 5 percent of the Grade 3 to 5 students in the sample could do division.

Nepal's schools were closed when the trial started in January 2021. While partial re-openings occurred mid-way through implementation, school disruptions (e.g. frequent closures and reopenings in response to COVID-19 infection waves) were common throughout the study. During this time, the government rolled out learning programs using radio, television, and online sources, and disseminated printed learning materials to students. However, few students were able to access existing remote educational support materials during school closures. Baseline data showed that in the status quo, only 31 percent of students had teacher interactions during school closure and less than five percent accessed radio or online education [14]. These gaps in remote learning access highlight the need to provide additional support, such as ongoing teacher phone calls to tutor their students.

The study sample included 10 local governments selected by the Ministry of Education, Science and Technology (MoEST) and World Bank, and covered all 7 provinces, with broad geographic spread in the country. The sample included public school students in grades 3 through 5.

To assess scalability, the phone call tutorials were implemented through both a government delivery treatment arm and an NGO delivery arm. Students were randomly assigned to either arm or a control group. The program was implemented by a coalition of partners including MoEST, local governments, the World Bank, Teach for Nepal, and Street Child. In the government arm, public school teachers implemented the program, with teachers teaching students from outside of their own region.[7] In the NGO arm, facilitators trained as teachers delivered the phone call tutoring program. Calls were conducted in over 12 languages, with students matched to teachers who spoke their language.

Philippines The Philippines scored second-lowest of 79 countries in the 2018 PISA mathematics assessment. During COVID-19, Philippines students also faced one of the world's longest school

[7] In an additional randomised trial discussed below, we used the over-subscribed list of eligible public school teachers in Nepal to randomly assign teachers to an implementation 'treatment' arm, or control arm. We then also measured causal effects of being an instructor on teacher beliefs and practices.

closures, with schools closed for over two years [11]. Only 2 percent of students in our baseline sample could do division, placing them far behind grade-level expectations; these baseline learning levels are some of the lowest in our study.

At the start of our Philippines study in August 2021, schools had been closed for almost 1.5 years, and they remained closed throughout the trial. During school closures, the government encouraged households to access educational content provided on radio, television, online, and printed materials. However our data reveals limited use of these educational materials, with fewer than 5 percent accessing radio and TV resources. These gaps, like in other countries, reveal the need for additional high-quality educational support.

The program was implemented in 3 of the country's 17 regions. These regions were selected by the government and represent some of the most marginalized communities in the country. Students from grades 3 and 4 were enrolled in the study.

In the Philippines, like Nepal, the phone call tutoring program was implemented through a government delivery treatment arm and an NGO delivery arm. In the government arm, public school teachers employed by the Philippines Department of Education implemented the program. Working with the government, Innovations for Poverty Action, a research NGO, trained government teachers from participating schools on the intervention. The teachers were assigned to call students at their school in the grade they taught. In the NGO-led teacher-aide arm, Innovations for Poverty Action in coordination with the central and regional government offices, hired and trained tutors from the pool of government teacher applicants to implement the program. Calls were conducted in the 5 most commonly spoken languages in the study regions.

Uganda Ugandan students are well below grade-level expectations in foundational skills. Recent data shows that just 6 percent of grade 4 students are able to read a paragraph and only 2 percent can solve a simple math problem [15]. On our baseline survey, only 14 percent of the Grade 3–5 students could do division.

The learning crisis was compounded when, like the Philippines, Uganda entered one of the world's longest COVID-induced school closures, with schools closed for nearly two years from March 2020 to January 2022 [11]. While the government provided distance learning support to households, engagement with these materials was limited. Only 29 percent of households engaged in radio lessons, 22 percent in printed self-study materials, and under 12 percent in TV and online [16]. Like in other contexts, this highlights the need for higher-engagement approaches, such as live phone calls.

The study took place in 9 out of Uganda's 135 Districts, which covered some of the most rural part of the country. The sample included students in grades 3 to 5.

In this study, the implementing organisation was Building Tomorrow, an education-focused NGO in Uganda. Phone calls were made by Building Tomorrow's Community Education Volunteers, resident members of communities who are recruited and trained to serve as grassroots education 'extension agents'. These volunteers encourage out-of-school children to enroll in school, and lead literacy and numeracy lessons in community settings. In Uganda, calls were conducted in one of three languages: English, Luganda, and Runyankore.

G. Key Components of the Intervention

In this section, we outline a framework with several key components which could drive the potential effectiveness of phone-based tutoring across contexts. We adapt a framework from a broader set of tutoring programs [17]. In this framework, there are several key inputs: curriculum characteristics, tutor typology, delivery mode, and dosage.

Curriculum Characteristics

There is a growing literature on the effectiveness of targeting teaching instruction to a student's level rather than their age or grade [18]. The principle of targeted instruction focuses on identifying each student's individual learning level through a light-touch learning assessment. Instructors then use this information to target their instruction and 'teach at the right level.' Targeting instruction stands in stark contrast with business-as-usual teaching in most education systems. In most systems, teachers typically teach to a one-size-fits-all grade-level curriculum. Yet most children are not at grade level. For example, while grade-level curricula often expect students in grades 3 to 5 to be able to do two-digit division, in our study sample, only 7 percent of students can do so. This phenomenon is pervasive. A survey in Kenya, Tanzania, and Uganda showed that three-quarters of students in grade 3 could not even read a simple sentence, falling well below grade-level expectations [7]. In this context, students being taught grade-level material will be left behind and stay behind. In contrast, targeting instruction to students' learning levels, focusing on foundational skills, via rapid assessment and teaching activities tailored to their level, enables students to learn far more effectively.

Targeted instruction approaches have received growing attention as highly cost-effective relative to status quo teaching and have been tested using various in-person and in-school models [18–21]. The phone call tutoring program tested in this study adapts the targeted instruction pedagogy for a remote setting, with frequent assessments conducted weekly to enable ongoing, flexible targeted instruction even in disrupted settings. In addition, since phone call tutorials are conducted one-on-one with students, they mimic some of the targeting benefits of tutoring programs, which are conducted in smaller groups, thus also enabling more targeted instruction to each child's level. Tutoring is an approach most often tested to date in high-income settings [17, 22]. We adapt tutoring approaches to low- and middle-income settings, testing a low-cost phone call tutorial model targeted to student levels and made affordable via use of mobile phones.

The degree of targeting instruction is likely to play a key role in the effectiveness of the intervention across contexts. We collect detailed data on rates of targeted instruction across contexts. Several other features of the curriculum include a focus on basic foundational numeracy skills. The simplicity of the curriculum content ensured the material could be delivered effectively via phone. The focus on foundational skills also addresses the most pressing learning gaps in our study settings, with over 70 percent of students in LMICs experiencing 'learning poverty', unable to do basic numeracy operations or read a simple text [7].

In terms of grades covered, in most settings students were in grades 3 through 5, where remedial instruction most often takes place. In Kenya, students were slightly younger, in grades 1 and 2.

Tutor Typology

We consider two main types of tutors: government teachers and NGO instructors. On the one hand, teachers have more experience, and might be more effective at adopting new pedagogical practices such as targeted instruction. On the other hand, teachers might have a hard time deviating from business as usual which often defaults to teaching to a one-size-fits-all curriculum, with NGO instructors more effectively able to adopt new pedagogy.

We experimentally vary whether phone call tutorials are provided by government teachers or NGO instructors in two settings – Nepal and the Philippines – in order to understand which tutor type is most effective, and to investigate implications for scalability. Both types of tutors received the same training by the same trainers (described in more depth in the intervention section).

Parents are engaged in sessions with their child at the household and are conduits for instruction.

This is the case for both government and NGO supported tutoring instruction across all settings. Since parents are engaged consistently across studies and treatment arms, the main variation we investigate is the differential effectiveness between government teachers and NGO instructors. Related follow-on work has explored the relative impact of further engaging parents in instruction [23].

Delivery Mode

The phone-based tutoring program studied leverages the scalable and affordable technology of mobile phones. The program was designed to reach people on a platform most households have easy access to. In our five study countries, average mobile phone penetration was 108 mobile phone subscriptions per 100 people (ITU 2021). Coverage is also high, with at least 97% of the world's population covered by at least a 2G mobile network, which is often sufficient to make and receive calls, and a minimum of 93% of households in our study countries.

Given high access to mobile phones, even in low resource settings, this platform presents a scalable way to deliver educational content to households [24, 25]. While other technology platforms also present some opportunities for educational instruction, such as television, radio, and online media, access is often lower than through mobile phones. In low income countries, less than 30 percent of households report owning a television, 50 percent a radio, and 20 percent having internet access, suggesting content delivered over these platforms is less likely to have high uptake, particularly amongst the most disadvantaged households [26]. This is confirmed by households in our sample who report low usage of educational resources delivered via non-phone platforms.

Phones also reduce the frictions that may be associated with some other platforms in emergency and low resource settings. For example, given phones are smaller than televisions, radios, and computers, they are often easily transportable in an emergency. Phones can also charge faster and last longer than computers and tablets. Finally, phone call interventions can also offer needed flexibility. While radio and TV programs often require caregivers to find dedicated time in their schedules, tuning into a pre-scheduled television or radio program, phone call programs can provide more flexible scheduling options, increasing take-up. Households receive calls from a tutor who solicits their interest in participating, then initiates the tutoring calls and schedules according to a household's availability, offering convenient and accessible sessions which lead to high engagement. Low-cost high-access platforms such as mobile phones can enable higher time-on-task and additional instruction relative to business as usual by ensuring high engagement.

Other dimensions of delivery mode include student-to-tutor ratios and whether the instruction provided was during school or after school. In our study, the ratio was 1-1 across all study settings. Sessions were always conducted out of school – either after school or during disruptions.

Dosage

Dosage is typically a large driver of variation in the effectiveness of tutoring. In our study, we aimed to standardize dosage as much as possible across contexts. The standard dosage of the intervention was around 8 weeks, each session consisting of 20-minutes of instruction per household. This dosage represents additional instructional time beyond the status quo, a mechanism by which learning gains are likely to accrue. At the same time, this dosage is highly efficient – a common feature of tutoring programs – with just a few hours of instruction total, representing a highly productive method to produce learning gains. This imposes minimal time costs on teachers and parents to limit crowd-out of other potentially productive activities. We explore potential crowd-out effects.

Table A1. Learning During Other Education in Emergencies — Typhoon in the Philippines

	Typhoon Effects			
	(1) Learning (SD)	(2) Learning (SD)	(3) Learning (SD)	(4) Learning (SD)
Affected by Typhoon	-0.213***	-0.134***		
	(0.053)	(0.051)		
	[0.000]	[0.009]		
Control × Typhoon Effect			-0.231**	-0.111
			(0.116)	(0.112)
			[0.047]	[0.323]
SMS messages × No Typhoon Effect			0.075	0.075
			(0.076)	(0.073)
			[0.319]	[0.303]
SMS messages × Typhoon Effect			-0.086	-0.003
			(0.088)	(0.085)
			[0.330]	[0.976]
Phone call and SMS × No Typhoon Effect			0.438***	0.445***
			(0.082)	(0.079)
			[0.000]	[0.000]
Phone call and SMS × Typhoon Effect			0.174*	0.223**
			(0.104)	(0.100)
			[0.094]	[0.026]
Observations	1759	1758	1759	1758
Countries Included	Philippines	Philippines	Philippines	Philippines
Controls	None	Ed and Bsl Level	None	Ed and Bsl Level

Notes. This table reports the effect of a typhoon on learning outcomes in the Philippines. Learning refers to how a child scores on four basic numeracy operations: no operations correct, addition, subtraction, multiplication, and division (measured on a scale of 0–4 and expressed in terms of standard deviations, standardized using the control group mean and standard deviation at endline). Column (1) shows the effect on learning of students affected by the typhoon. Column (2) accounts for the baseline distribution and the caregiver's education. Columns (3) and (4) show the results of the SMS messages alone as well as phone tutorial treatments with and without controls. Standard errors are in parentheses and p-values are in square brackets. Given learning outcomes are standardized to have a control mean of zero, the control mean row reports the unstandardized average student learning level in the control group at endline (0 = no operations, 1 = addition, 2 = subtraction, 3 = multiplication, 4 = division).

Table A2. Parent and Child Beliefs

	Beliefs about child's level and progress			Caregiver's program interest	
	(1) Child estimates level	(2) Caregiver estimates level	(3) Child progressed	(4) Wants program	(5) WTP
SMS messages	0.040*	0.075***	0.010	-0.019	0.060***
	(0.024)	(0.024)	(0.015)	(0.012)	(0.020)
	[0.097]	[0.002]	[0.522]	[0.127]	[0.003]
Phone call and SMS	0.210***	0.114***	0.104***	0.034***	0.045**
	(0.023)	(0.023)	(0.014)	(0.011)	(0.020)
	[0.000]	[0.000]	[0.000]	[0.001]	[0.025]
Observations	8798	9188	6188	8918	3777
Control mean	2.347	2.285	0.350	0.968	0.434
P-Val: SMS vs Phone Call + SMS	0.000	0.079	0.000	0.000	0.452
Country Fixed Effects	Yes	Yes	Yes	Yes	Yes
Grade Fixed Effects	Yes	Yes	Yes	Yes	Yes
Countries Included	All	All	4	All	Philippines & Uganda

Notes. This table shows treatment effects on secondary outcomes related to parental and child beliefs, and demand for the program. Column (1) measures the child's belief about their level of learning, from beginner (0) to division (4), in standard deviations. Column (2) measures parents' beliefs about their child's level on the same scale. Column (3) shows treatment effects on an indicator variable for caregivers being very confident that their child has progressed in their learning. Column (4) shows effects on a dummy variable that the caregiver would like access to a phone-based maths tutoring program in the future, and Column (5) shows treatment effects on a dummy variable that caregivers would be willing to pay money to access a phone-based maths tutoring program in the future. Standard errors are in parentheses and p-values are in square brackets.

Table A3. Child Non-Cognitive Outcomes

	Non-cognitive skills		Wellbeing	
	(1)	(2)	(3)	(4)
	Perseverance	Ambition	Enjoys school	Often worried
SMS messages	0.007	0.004	0.010	0.002
	(0.010)	(0.011)	(0.011)	(0.012)
	[0.474]	[0.699]	[0.350]	[0.857]
Phone call and SMS	0.028***	0.061***	0.023**	-0.019*
	(0.009)	(0.011)	(0.009)	(0.010)
	[0.003]	[0.000]	[0.015]	[0.061]
Observations	8962	8962	8880	8121
Control mean	0.826	0.226	0.829	0.202
P-Val: SMS vs Phone Call + SMS	0.020	0.000	0.166	0.043
Country Fixed Effects	Yes	Yes	Yes	Yes
Grade Fixed Effects	Yes	Yes	Yes	Yes
Countries Included	All	All	All	All

Notes. This table shows treatment effects on children's non-cognitive outcomes, including perseverance and wellbeing. Column (1) measures impacts on a dummy variable for whether the child wanted to answer a second riddle question after answering an initial one. Column (2) measures impacts on an indicator variable for whether the student wanted to answer a second more difficult riddle question, indicating ambition (coded 0 if they didn't want to answer a second riddle question or they wanted an easier question). Column (3) shows effects on a dummy variable for whether the student says they enjoy school very much. Column (4) shows effects on an indicator for whether the child has many worries or is often worried, from the children's "Strengths and Difficulties Questionnaire". Standard errors are in parentheses and p-values are in square brackets.

Table A4. Potential for Crowd-out

	Caregiver supported child educ		Caregiver unemployment	
	(1)	(2)	(3)	(4)
	Did educ activities	Often did educ activities	Unemployed	Mother unemployed
SMS messages	0.048***	0.043***	-0.022	-0.029
	(0.009)	(0.013)	(0.021)	(0.026)
	[0.000]	[0.001]	[0.295]	[0.262]
Phone call and SMS	0.046***	0.035***	0.009	0.015
	(0.008)	(0.012)	(0.020)	(0.025)
	[0.000]	[0.004]	[0.665]	[0.551]
Observations	8899	8899	3681	2446
Control mean	0.856	0.481	0.409	0.409
P-Val: SMS vs Phone Call + SMS	0.781	0.561	0.106	0.059
Country Fixed Effects	Yes	Yes	Yes	Yes
Grade Fixed Effects	Yes	Yes	Yes	Yes
Countries Included	All	All	Philippines & Uganda	Philippines & Uganda

Notes. This table shows treatment effects on outcomes that could indicate the program crowds-out or crowds-in other education and labour market activities. Column (1) measures impacts on a dummy variable for whether the caregiver says they did any educational activities with their child over the past 3 weeks. Column (2) shows how often the parent did any educational activities with their child 3 or more times in the past week. Column (3) shows effects on caregiver unemployment, and column (4) restricts this unemployment estimate to households reporting that mothers are the primary educational caregiver in the household. Standard errors are in parentheses and p-values are in square brackets.

Table A5. Learning Outcomes at Scale Across Five Countries, incl. MHT-adjusted q-values

	Pooled full sample		Weighted by country	
	(1) Learning (SD)	(2) Learning (SD)	(3) Learning (SD)	(4) Learning (SD)
SMS messages	0.078***	0.069**	0.073**	0.069**
	(0.028)	(0.027)	(0.028)	(0.027)
	[0.006]	[0.010]	[0.010]	[0.011]
	{0.004}	{0.006}	{0.006}	{0.006}
Phone call and SMS	0.321***	0.312***	0.341***	0.336***
	(0.026)	(0.025)	(0.028)	(0.027)
	[0.000]	[0.000]	[0.000]	[0.000]
	{0.001}	{0.001}	{0.001}	{0.001}
Observations	9148	9148	9148	9148
Control Mean	1.726	1.726	1.726	1.726
P-Val: SMS vs Phone Call + SMS	0.000	0.000	0.000	0.000
Country Fixed Effects	Yes	Yes	Yes	Yes
Grade Fixed Effects	No	Yes	No	Yes
Countries Included	All 5	All 5	All 5	All 5

Notes. This table reports treatment effects on learning outcomes for a pooled sample as well as samples weighted by country. Learning is expressed in standardized units (measured on a scale of 0–4 and expressed in terms of standard deviations, standardized using the control group mean and standard deviation at endline). It includes False Discovery Rate (FDR) sharpened q-values to adjust for multiple hypothesis testing. Standard errors are in parentheses, p-values in square brackets, and sharpened q-values in curly brackets. Given learning outcomes are standardized to have a control mean of zero, the control mean row reports the unstandardized average student learning level in the control group at endline (0 = no operations, 1 = addition, 2 = subtraction, 3 = multiplication, 4 = division).

Table A6. Balance in Baseline Teacher Characteristics across Treatment Groups in Nepal

	(1) Female	(2) Years teaching	(3) High school	(4) Bachelors	(5) Masters	(6) Speaks English
Treatment Teacher	0.037	0.567	0.004	0.004	-0.034	0.044
	(0.056)	(1.167)	(0.057)	(0.056)	(0.039)	(0.056)
	[0.504]	[0.627]	[0.947]	[0.941]	[0.380]	[0.434]
Observations	301	301	301	301	301	301
Control mean	0.353	13.387	0.427	0.373	0.147	0.360

Notes. This table reports the differences in means at baseline between the teachers randomized into the program and those who were not assigned to the program in Nepal. Column (1) shows balance on teacher gender. Column (2) shows balance based on teacher's years of experience. Columns (3) to (5) show balance on the share of teachers whose highest level of education completed is high school, bachelors degree, or masters degree, respectively. Column (6) shows balance on the share of teachers who speak English. Standard errors are in parentheses and p-values are in square brackets.

Table A7. Learning Outcomes by All Four Operations Taught

	(1) Addition correct	(2) Subtraction correct	(3) Multiplication correct	(4) Division correct
SMS messages	-0.007	0.032**	0.066***	0.029***
	(0.012)	(0.014)	(0.015)	(0.010)
	[0.584]	[0.022]	[0.000]	[0.006]
Phone call and SMS	0.040***	0.116***	0.188***	0.135***
	(0.010)	(0.012)	(0.014)	(0.011)
	[0.000]	[0.000]	[0.000]	[0.000]
Observations	9148	9148	7163	7163
Control Mean	0.779	0.550	0.342	0.146
P-Val: SMS vs Phone Call + SMS	0.000	0.000	0.000	0.000
Country Fixed Effects	Yes	Yes	Yes	Yes
Grade Fixed Effects	Yes	Yes	Yes	Yes
Countries Included	5	5	4	4

Notes. This table reports learning outcomes across the four different proficiencies taught in the curriculum and comprising the standardized learning outcome. Column (1) highlights the learning gains in terms of share of students who got addition correct. Columns (2), (3), and (4) show the share of students who could correctly answer subtraction, multiplication, and division respectively. The higher-order questions were not asked of the Grade 1 and 2 students in Kenya as these were not covered in their standard school curriculum at these ages. Standard errors are in parentheses and p-values are in square brackets.

Table A8. Representativeness of the Sample at Endline

	Baseline Variables				
	(1)	(2)	(3)	(4)	(5)
	Baseline Learning	Grade 3	Grade 4	Grade 5	Student is Female
Randomized to receive endline survey	-0.009	-0.000	-0.003	0.004	-0.005
	(0.021)	(0.006)	(0.006)	(0.005)	(0.010)
	[0.679]	[0.967]	[0.558]	[0.462]	[0.634]
Observations	12707	16936	16936	16936	16936
Comparison Mean	1.39	0.35	0.37	0.28	0.51
Country Fixed Effects	Yes	Yes	Yes	Yes	Yes
Countries Included	All 5	All 5	All 5	All 5	All 5

Notes. This table reports balance on baseline characteristics of the sample that was randomly selected to be inter- viewed at endline, compared to the complete study sample. Column (1) shows representativeness by baseline learning; columns (2), (3) and (4) by student grade in the eligible sample; and column (5) by student sex. These are all of the variables collected across all five countries at baseline. In Nepal, a randomly selected half of the sample received a full baseline survey that included learning level. Learning is expressed in standardized units (a scale of 0–4 standardized using the control group mean and standard deviation at endline). In Kenya, school assessment grades were used in lieu of a baseline levelling assessment, and not all Kenyan students had pre-existing student grades; sample means for grades are calculated for students in countries with the relevant grades. Standard errors are in parentheses and p-values are in square brackets.

Table A9. Reach and Attrition

	Endline Response		Correlation in Attempts
	(1)	(2)	(3)
	Responded at Endline	Responded at Endline	Baseline Learning
SMS messages	-0.006	-0.005	
	(0.010)	(0.010)	
	[0.599]	[0.605]	
Phone call and SMS	0.002	0.001	
	(0.010)	(0.010)	
	[0.857]	[0.903]	
Number of attempts to reach HH			0.001
			(0.005)
			[0.889]
Observations	12331	12331	7187
Control mean	0.759	0.759	
Country Fixed Effects	Yes	Yes	Yes
Grade Fixed Effects	No	Yes	Yes
Countries Included	All 5	All 5	All 5

Notes. This table reports differences in endline response. Column (1) measures whether the student responded to the endline assessment. Column (2) measures the same but accounts for the students' grade level. Column (3) shows the effect of the number of attempts to reach a household on the baseline learning of the student. The control means show that on average 76 percent of students responded at endline. Standard errors are in parentheses and p-values are in square brackets.

Table A10. Balance on Baseline Characteristics

	Baseline Variables				
	(1) Baseline Learning	(2) Grade 3	(3) Grade 4	(4) Grade 5	(5) Female
SMS messages	0.035	-0.001	0.007	-0.006	0.016
	(0.027)	(0.013)	(0.013)	(0.008)	(0.012)
	[0.191]	[0.955]	[0.571]	[0.393]	[0.181]
Phone call and SMS	0.007	-0.001	0.008	-0.006	0.004
	(0.029)	(0.012)	(0.012)	(0.007)	(0.011)
	[0.801]	[0.906]	[0.520]	[0.352]	[0.725]
Observations	9376	12331	12331	12331	12331
Control Mean	1.47	0.34	0.34	0.24	0.53
P-Val: SMS vs Phone Call + SMS	0.36	0.95	0.96	0.98	0.29
Country Fixed Effects	Yes	Yes	Yes	Yes	Yes
Countries Included	All 5	All 5	All 5	All 5	All 5

Notes. This table reports the balance across treatment groups at baseline for the sample randomly selected to be interviewed at endline. Column (1) shows balance on baseline learning; columns (2), (3) and (4) by student grade in the eligible sample; and column (5) by student sex. These are all of the variables collected across all five countries at baseline. In Nepal, a randomly selected half of the sample received a full baseline survey that included learning level. Learning is expressed in standardized units (a scale of 0–4 standardized using the control group mean and standard deviation at endline). In Kenya, school assessment grades were used in lieu of a baseline levelling assessment, and not all Kenyan students had pre-existing student grades. Standard errors are in parentheses and p-values are in square brackets.

Figure A1. Learning Outcomes (SD) at Scale across Five Countries and Scaled by Government

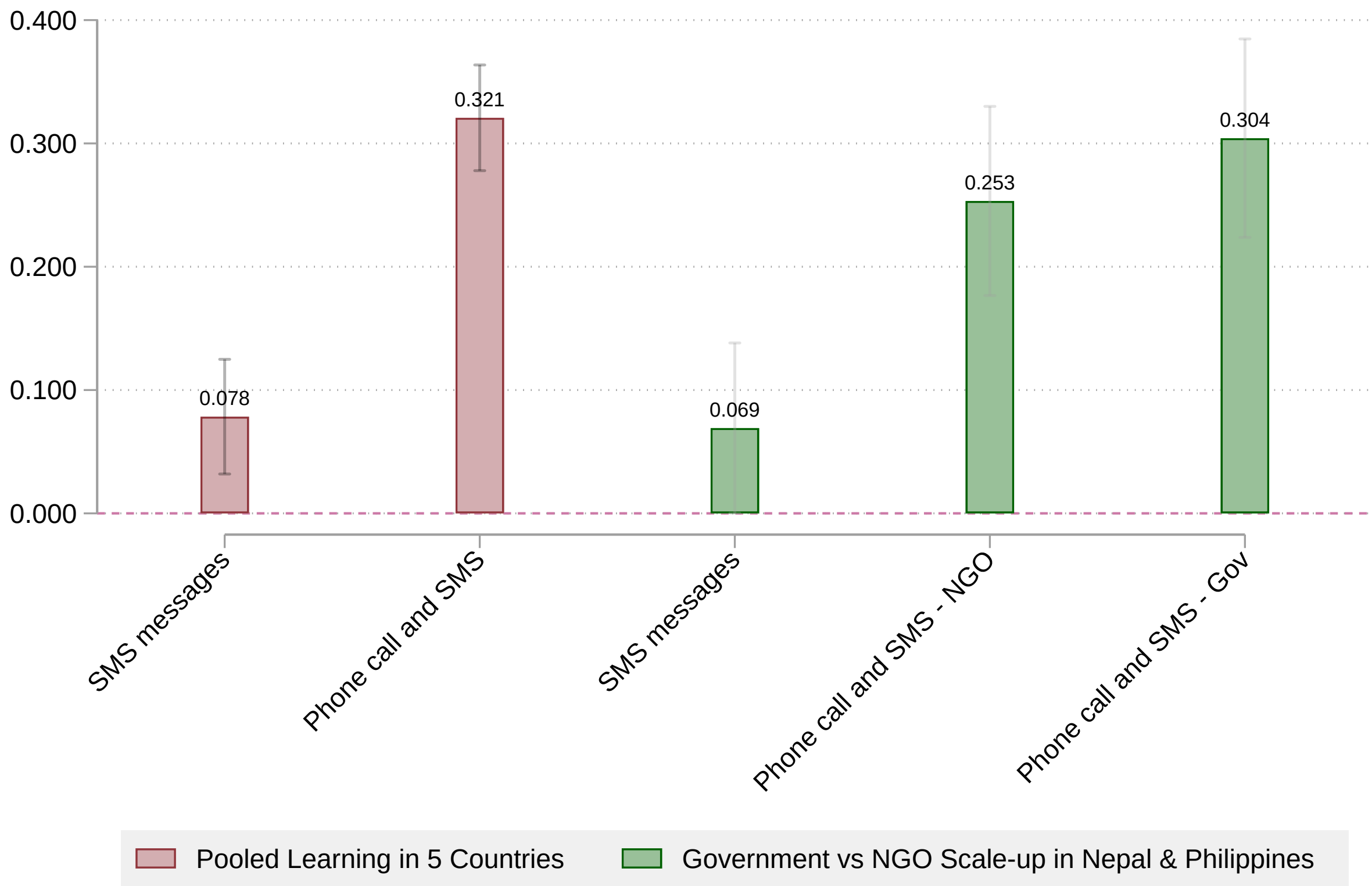

Notes. This figure shows treatment effects on learning outcomes. Data are presented as treatment effects relative to the control group ±90 percent confidence intervals, expressed in standard deviations. Learning refers to how a child scores on four basic numeracy operations (no operations correct, addition, subtraction, multiplication, and division), for which we report the average level on a scale of 0–4. The colours distinguish between the pooled learning in five countries and the government vs. NGO scale-up in Nepal and the Philippines.

Figure A2. Timeline of Trials

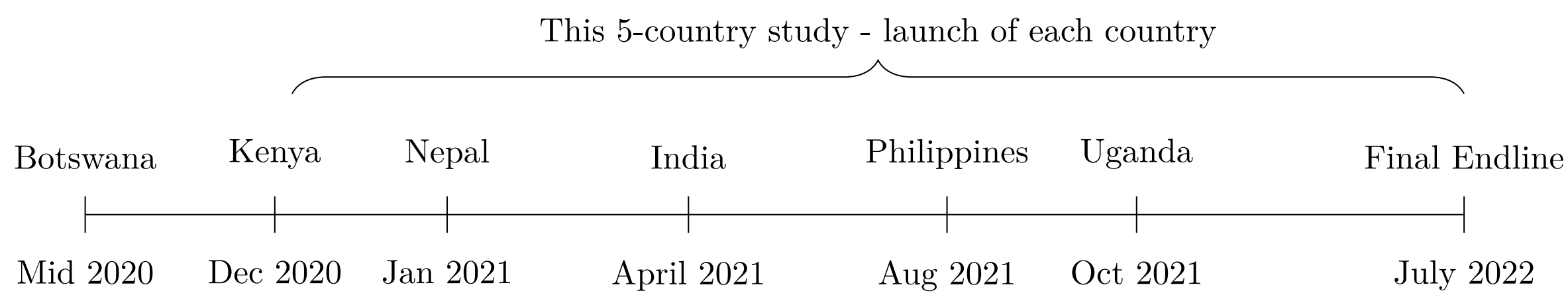

Notes. This figure shows the timing of the implementation across studies. The proof-of-concept study was conducted in Botswana in 2020. It was followed by five studies across five countries. An endline assessment was conducted a few months after each implementation period ended; the endline for the latest replication was in July 2022.

Figure A3. Distribution of Learning at Baseline and Endline across Groups

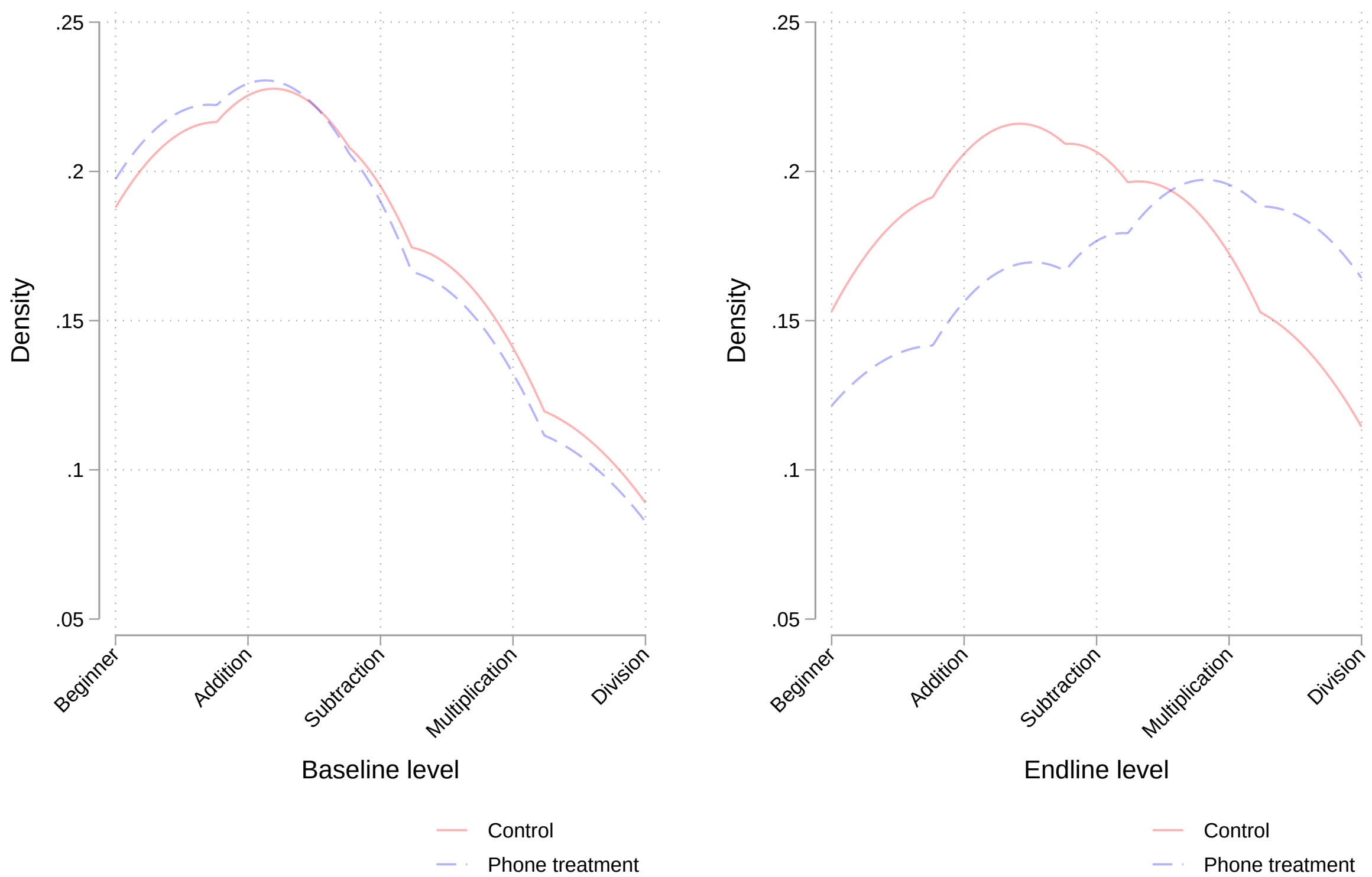

Notes. These figures show the distribution of student math levels by control group and phone call treatment group at baseline (left) and endline (right). 'Level' is the highest level students achieved in the ASER-based assessment, which starts at Beginner (can't perform any numerical operations), and then proceeds to Addition, Subtraction, Multiplication, and ends at Division. Additional proficiencies were also tested, such as word problems and fractions, but the core competencies listed above comprise the common ASER learning module across countries. These density plots include all countries except Kenya, since in Kenya baseline school exam scores were used instead of a new baseline assessment.

Figure A4. COVID-19 Infections and School Disruption

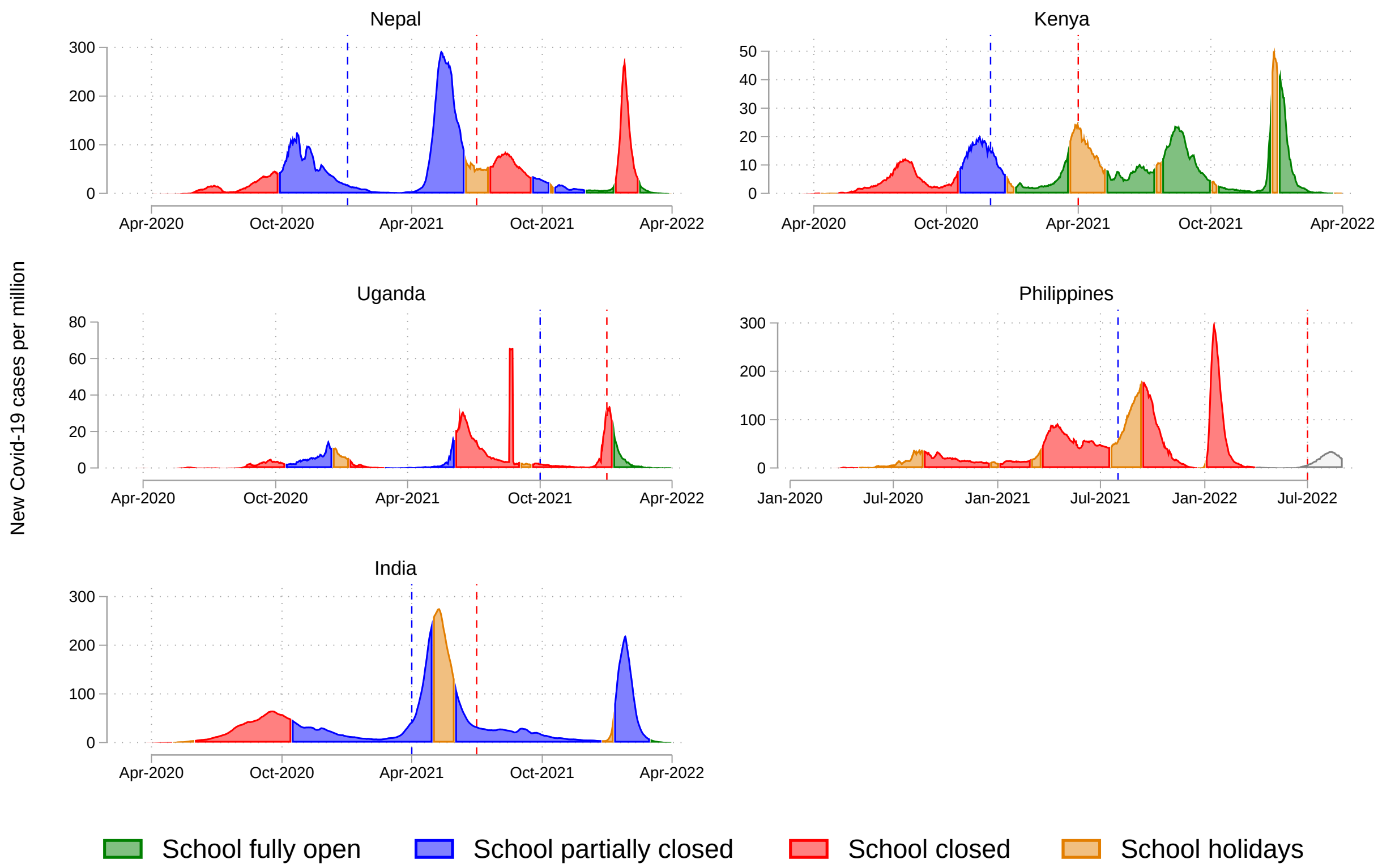

Notes. This figure shows the evolution of COVID-19 cases (new cases per million people) and resulting school closures or disruptions over two years. Each panel represents a country in our five-country study, with the blue line indicating the approximate baseline collection date and the red line indicating the approximate endline collection date. The Philippines and Nepal had two cycles in succession, an NGO and a government delivery cycle, each 8 weeks long. Data sources include the Our World in Data COVID-19 dataset and the UNESCO global monitoring dataset of school closures.

Figure A5. CONSORT Diagram of Participant Flow

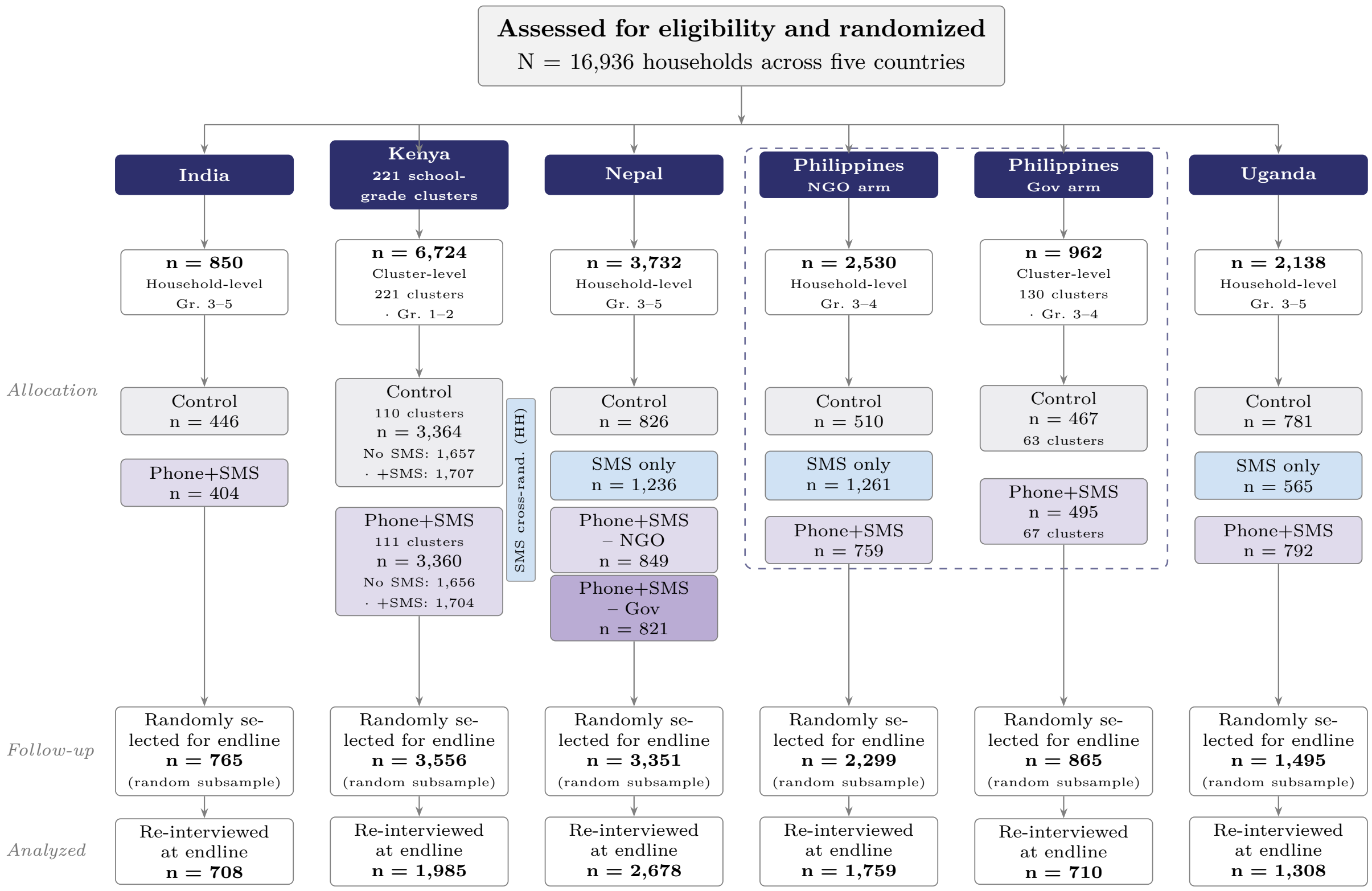

Notes. The Philippines government trial used school-grade cluster randomization. The Kenya trial also used school- grade cluster randomization for the control and phone call + SMS treatment groups, with SMS-only cross-randomized at the household level. All other trials used household-level randomization. Treatment arms and allocation ratios varied by country depending on budget, sample size, and implementation context. The "analyzed" row reports the endline sample with a valid comparable learning score, matching the sample sizes in Table E1.

References

1. Hanna, R., Mullainathan, S. & Schwartzstein, J. Learning through noticing: Theory and evidence from a field experiment. *The Quarterly Journal of Economics* **129,** 1311–1353 (2014).
2. Bergman, P. Parent-child information frictions and human capital investment: Evidence from a field experiment. *Journal of Political Economy* **129,** 286–322 (2021).
3. Jackson, C. K. What do test scores miss? The importance of teacher effects on non–test score outcomes. *Journal of Political Economy* **126,** 2072–2107 (2018).
4. Carlana, M. & La Ferrara, E. Apart but connected: Online tutoring, cognitive outcomes, and soft skills. *American Economic Review* **115,** 3487–3513 (2025).
5. Evans, D. K., Jakiela, P. & Knauer, H. A. The impact of early childhood interventions on mothers. *Science* **372,** 794–796 (2021).
6. Anderson, M. L. Multiple inference and gender differences in the effects of early intervention: A reevaluation of the Abecedarian, Perry Preschool, and Early Training Projects. *Journal of the American Statistical Association* **103,** 1481–1495 (2008).
7. World Bank. *World Development Report 2018: Learning to realize education's promise* (2018).
8. Azevedo, J. P., Hasan, A., Goldemberg, D., Geven, K. & Iqbal, S. A. Simulating the potential impacts of COVID-19 school closures on schooling and learning outcomes: A set of global estimates. *The World Bank Research Observer* **36,** 1–40 (2021).
9. Burde, D., Guven, O., Kelcey, J., Lahmann, H. & Al-Abbadi, K. *What works to promote children's educational access, quality of learning, and wellbeing in crisis-affected contexts* Education Rigorous Literature Review (Department for International Development, London, 2015).
10. Angrist, N., Bergman, P. & Matsheng, M. Experimental evidence on learning using low-tech when school is out. *Nature Human Behaviour,* 1–10 (2022).
11. UNESCO. *COVID-19 Education Response* https://covid19.uis.unesco.org/global-monitoring-school-closures-covid19/country-dashboard/. 2023.
12. Uwezo. *Are Our Children Learning? Kenya Sixth Learning Assessment Report* tech. rep. (Nairobi, 2016).
13. UNESCO. *Sustainable Development Goal (SDG) 4 Kenya Country Profile* tech. rep. (2016).
14. Radhakrishnan, K. *et al. Learning in the Time of COVID-19: Insights from Nepal* tech. rep. (World Bank, Washington, DC, 2021).
15. World Bank. *Uganda Economic Update, 13th Edition, May 2019: Economic Development and Human Capital in Uganda - A Case for Investing More in Education* tech. rep. (Washington, DC, 2019).
16. Uwezo. *Are our Children Learning? Illuminating the Covid-19 Learning Losses and Gains in Uganda. Uwezo National Learning Assessment Report, 2021* tech. rep. (Kampala, 2021).
17. Nickow, A., Oreopoulos, P. & Quan, V. *The impressive effects of tutoring on prek-12 learning: A systematic review and meta-analysis of the experimental evidence* tech. rep. (2020).
18. Banerjee, A. *et al.* From proof of concept to scalable policies: Challenges and solutions, with an application. *Journal of Economic Perspectives* **31,** 73–102 (2017).
19. Muralidharan, K., Singh, A. & Ganimian, A. J. Disrupting education? Experimental evidence on technology-aided instruction in India. *American Economic Review* **109,** 1426–60 (2019).
20. Duflo, A., Kiessel, J. & Lucas, A. *External Validity: Four Models of Improving Student Achievement* Working Paper w27298 (National Bureau of Economic Research, Cambridge, 2020).

21. Angrist, N. & Meager, R. Implementation Matters: Generalizing Treatment Effects in Education. *Available at SSRN 4487496* (2023).
22. Robinson, C. D. & Loeb, S. *High-impact tutoring: State of the research and priorities for future learning* Report 21 (National Student Support Accelerator, 2021), 1–53.
23. Angrist, N., Cullen, C. & Magat, J. Cheaper (and more effective) by the dozen: Evidence from 12 randomised A/B tests optimising tutoring for scale (2025).
24. Aker, J. C. & Mbiti, I. M. Mobile phones and economic development in Africa. *Journal of Economic Perspectives* **24,** 207–32 (2010).
25. Aker, J. C., Ksoll, C. & Lybbert, T. J. Can mobile phones improve learning? Evidence from a field experiment in Niger. *American Economic Journal: Applied Economics* **4,** 94–120 (2012).
26. Carvalho, S. & Crawfurd, L. *School's Out: Now What?* Center for Global Development. https://www.cgdev.org/blog/schools-out-now-what. 2020.

C Survey Tools

Building Resilient Education Systems: Baseline Questionnaire

1. **Did you speak to the household?**
   ○ Yes
   ○ No
2. **Did the child's caregiver provide consent to participate?**
   ○ Yes
   ○ No
3. **If no consent was given, why?**
4. **What is the student's name and surname?** _______________
5. **In what school is the student enrolled?** _______________
6. **In what district/region is the school located?** _______________
7. **What class/grade is the student currently enrolled in?** _______________
8. **What is the student's age?** *[Note:This question was asked in India, Uganda and the Philippines.]*
9. **What is the student's gender?** _______________

MATH LEARNING MODULE: PROTOCOL

- Inform the caregiver that you would like the children to work on math problems.
- Ask the caregiver to place the call on speakerphone. They may repeat questions for the children to answer.
- Request that children answer math problems on their own on a scrap paper, including their answer. Children should work alone and not copy off anyone or work together.
- Explain that this is not an exam/test, so it's okay if the child(ren) do not get the answers correct.
- Children should take no longer than (30 seconds for Place Value) or (2 minutes for regular operation) to answer the question. If it seems someone is helping, gently ask them to refrain. If someone continues to help and/or the child takes longer than (30 seconds for place value) or (2 minutes for operations), mark that the child got this wrong.
- When finished, request that the child read out each of their answers and explain their answer to you. This is not an exam/test, so it's okay if your child(ren) does not get the answers correct. Are the child(ren) ready?
- Record all correct responses from each child and make a note of the highest operation that each child can perform.
- All students begin by answering the PLACE VALUE question and proceed with the LEARNING MODULE regardless of whether they answer with a correct response.

10. **Prativa has 32 apples and organizes them by PLACE VALUE. How many TENS does she have?**
    - ○ The child got the question correct
    - ○ The child got the question incorrect
    - ○ The child gives the correct answer but is not able to convincingly explain how they got their answer/I don't believe they answered it themselves.
    - ○ The parent was answering for the child/not letting the child answer, or the child used a calculator
11. **The student solves: 34 + 47**
    - ○ The child got the question correct
    - ○ The child got the question incorrect
    - ○ The child gives the correct answer but is not able to convincingly explain how they got their answer/I don't believe they answered it themselves.
    - ○ The parent was answering for the child/not letting the child answer, or the child used a calculator
12. **The student solves: 83 - 45**
    - ○ The child got the question correct
    - ○ The child got the question incorrect
    - ○ The child gives the correct answer but is not able to convincingly explain how they got their answer/I don't believe they answered it themselves.
    - ○ The parent was answering for the child/not letting the child answer, or the child used a calculator
13. **The student solves: 23*4 (23 multiplied by 4)**
    - ○ The child got the question correct
    - ○ The child got the question incorrect
    - ○ The child gives the correct answer but is not able to convincingly explain how they got their answer/I don't believe they answered it themselves.
    - ○ The parent was answering for the child/not letting the child answer, or the child used a calculator
14. **The student solves: 80/9 (80 divided by 9)**
    - ○ The child got the question correct
    - ○ The child got the question incorrect
    - ○ The child gives the correct answer but is not able to convincingly explain how they got their answer/I don't believe they answered it themselves.
    - ○ The parent was answering for the child/not letting the child answer, or the child used a calculator
15. **What languages is the learner able to speak?** _______________

16. **What is the main caregiver's highest level of education?** ***[Note: This question was asked at baseline in Nepal and Philippines and endline in other countries. Answer choices were adjusted to the contexts where necessary.]***
    - ○ None/ Informal Education
    - ○ Primary Education
    - ○ High School
    - ○ Certificate/ Vocational
    - ○ College/ Bachelor's Degree
    - ○ Master's Degree or higher
    - ○ Respondent does not know
17. **What is the best phone number to reach the student?** ______________
18. **Who is the owner of this phone (first name and surname)?** ______________
19. **What is the relationship of the phone owner to the student?** ______________
20. **Please list one alternate phone number where the child can be reached.** ______________

Building Resilient Education Systems: Endline Questionnaire

1. **Did you speak to the person that typically helps the child with math problems and schoolwork?**
   - ○ Yes
   - ○ No
2. **Did they provide consent to participate in the endline survey?**
   - ○ Yes
   - ○ No
3. **Who typically provides educational instruction or support to the child outside of school?**
   - ○ Mother
   - ○ Father
   - ○ Grandparent
   - ○ Sibling
   - ○ Others not mentioned above in the household
   - ○ Teacher
   - ○ Adults from the community who do not live in the household
4. **What is the main caregiver's highest level of education?**
   - ○ None/ Informal Education
   - ○ Primary Education
   - ○ High School
   - ○ Certificate/ Vocational
   - ○ College/ Bachelor's Degree
   - ○ Master's Degree or higher
   - ○ Respondent does not know
5. **How confident do you feel that [student name] made progress in learning over the past three months?**
   - ○ The child would have significant difficulty performing any operation
   - ○ Not confident at all
   - ○ Slightly confident
   - ○ Moderately confident
   - ○ Very confident

6. **What is the highest operation that you think the child can easily perform?**
   - ○ The child would have significant difficulty performing any operation
   - ○ Addition
   - ○ Subtraction
   - ○ Multiplication
   - ○ Division
   - ○ Respondent does not know

7. **Would you be interested in receiving phone-based education support in the future?**
   - ○ Yes, through both phone calls and SMS
   - ○ Yes, only through SMS
   - ○ Yes, only through phone calls
   - ○ Yes, through either phone calls or SMS
   - ○ No, they are not interested

8. **Over the past four weeks, how often did you spend time doing educational activities (in general) with [student name]? For example, reading, practicing math problems, composition, etc.**
   - ○ Never
   - ○ Less than once per week
   - ○ 1-2 times per week
   - ○ 3-4 times per week
   - ○ 5 or more times per week

9. **Over the past four weeks, how often did you spend time doing educational activities (in general) with children in your household OTHER than [student name]? For example, reading, practicing math problems, composition, etc.**
   - ○ Never
   - ○ Less than once per week
   - ○ 1-2 times per week
   - ○ 3-4 times per week
   - ○ 5 or more times per week

10. **Is the child currently in school?**
    - ○ Yes - in person has been in the same school for the past 3 months
    - ○ Yes - in person switched to a new school in the past 3 months
    - ○ No - not in school currently because school is closed but will return when school is open
    - ○ No - not in school currently because school is closed and unlikely to return when schools reopen
    - ○ No - not in school currently. School is open but the student has not returned.
    - ○ F. Respondent refused to answer *[Note: These answer options were slightly adjusted to suit the context if necessary.]*

11. **Were SMS messages that had math problems sent to you and [student name] over the past [context-specific time frame]?**
   - ○ Yes
   - ○ No
   - ○ Respondent does not know
12. **Over the past [context-specific time frame], how often did the child practice math problems that were sent to you in an SMS message?**
   - ○ Always
   - ○ Frequently
   - ○ Sometimes
   - ○ Rarely
   - ○ Never
   - ○ The respondent does not know
13. **How many phone calls for math instruction did the child receive over [context-specific time frame]?**
   Integer
14. **Is the person that typically helps the child with their math problems / schoolwork currently working?**
   - ○ Yes, full-time
   - ○ Yes, part-time
   - ○ No, they have retired
   - ○ No, they are unemployed
15. **How true is the following statement about the child? - The child has many worries, or often seems worried.**
   - ○ Not true
   - ○ Somewhat true
   - ○ Definitely true
16. **Hypothetically, how much would you be willing to pay for your child to receive weekly 1-on-1 math phone calls to support their learning?**
   - ○ Nothing
   - ○ A little
   - ○ A lot
17. **How much does [student name] enjoy school?**
   - ○ Very much
   - ○ Somewhat
   - ○ Not much
   - ○ They do not like school at all

18. Which maths problems does the child say they can easily perform?

- ○ The child would have significant difficulty performing any operation
- ○ Addition
- ○ Subtraction
- ○ Multiplication
- ○ Division
- ○ Respondent does not know

CORE LEARNING MODULE PROTOCOL

- Inform the caregiver that you would like the children to work on math problems.
- Ask the caregiver to place the call on speakerphone. They may repeat questions for the children to answer.
- Request that children answer math problems on their own on a scrap paper, including their answer. Children should work alone and not copy off anyone or work together.
- Explain that this is not an exam/test, so it's okay if the child(ren) do not get the answers correct.
- Children should take no longer than (30 seconds for Place Value) or (2 minutes for regular operation) to answer the question. If it seems someone is helping, gently ask them to refrain. If someone continues to help and/or the child takes longer than (30 seconds for place value) or (2 minutes for operations), mark that the child got this wrong.
- When finished, request that the child read out each of their answers and explain their answer to you. This is not an exam/test, so it's okay if your child(ren) does not get the answers correct. Are the child(ren) ready?
- Record all correct responses from each child and make a note of the highest operation that each child can perform.
- All students begin by answering the PLACE VALUE question and proceed with the LEARNING MODULE regardless of whether they answer with a correct response.

19. Prativa has 47 apples and organizes them by PLACE VALUE. How many TENS does she have?

- ○ The child got the question correct
- ○ The child got the question incorrect
- ○ The child gives the correct answer but is not able to convincingly explain how they got their answer/ I don't believe they answered it themselves.
- ○ The parent was answering for the child/not letting the child answer, or child used a calculator

20. The student solves: 52 + 39

- ○ The child got the question correct
- ○ The child got the question incorrect
- ○ The child gives the correct answer but is not able to convincingly explain how they got their answer/ I don't believe they answered it themselves.
- ○ The parent was answering for the child/not letting the child answer, or child used a calculator

21. The student solves: 42 - 29

- ○ The child got the question correct
- ○ The child got the question incorrect
- ○ The child gives the correct answer but is not able to convincingly explain how they got their answer/ I don't believe they answered it themselves.
- ○ The parent was answering for the child/not letting the child answer, or child used a calculator

22. The student solves: 28*3 (28 multiplied by 3)

- ○ The child got the question correct
- ○ The child got the question incorrect
- ○ The child gives the correct answer but is not able to convincingly explain how they got their answer/ I don't believe they answered it themselves.
- ○ The parent was answering for the child/not letting the child answer, or child used a calculator

23. The student solves: 65/8 (65 divided by 8)

- ○ The child got the question correct
- ○ The child got the question incorrect
- ○ The child gives the correct answer but is not able to convincingly explain how they got their answer/ I don't believe they answered it themselves.
- ○ The parent was answering for the child/not letting the child answer, or child used a calculator

24. A man drives 24km. Then he drives 17km. How many km did he drive in total?

- ○ The child got the question correct
- ○ The child got the question incorrect
- ○ The child gives the correct answer but is not able to convincingly explain how they got their answer/ I don't believe they answered it themselves.
- ○ The parent was answering for the child/not letting the child answer, or child used a calculator

25. I will now ask you a word riddle. If the day before yesterday was Tuesday, what day is it?

- ○ The student got the answer correct
- ○ The student did not get the answer correct within 45 seconds.

26. Would you like to try to answer another logic question?

- ○ Yes, I'd like to try with a question as difficult as this one
- ○ Yes, but I'd like to try an easier question
- ○ No

27. The student solves: 3/8 + 4/8 (three eighths plus four eighths)

- ○ The child got the question correct
- ○ The child got the question incorrect
- ○ The child gives the correct answer but is not able to convincingly explain how they got their answer/ I don't believe they answered it themselves.
- ○ The parent was answering for the child/not letting the child answer, or child used a calculator

NOTE ON ROBUSTNESS CHECKS

Multiple learning robustness tests were conducted, both within and separate to the surveys. These included: survey backchecks where a random subset of households were re-surveyed on learning outcomes (India); students being randomly assigned to answer different math problems (with different numerals) of the same difficulty level (Nepal); an in-person assessment of the same math problems (Kenya); and the above listed effort riddle question to measure real student effort, distinct from learning. The results of these robustness tests are shown in Table E5.

D Education in Emergencies Database References

Various sources were used to build the database for school disruptions from 2005 to 2023. This data is not meant to cover the universe of disruptions but rather to provide an illustrative snapshot of the degree to which school disruptions happen to start to capture their frequency. Our search included news articles, as well as reports from organizations such as Save the Children, UNICEF's Children's Climate Risk Index, ReliefWeb by the United Nations Office for the Coordination of Humanitarian Affairs (OCHA), and the Global Assessment Report on Disaster Risk Reduction. Major climate disruptions were identified through the international disaster database (EM-DAT). Below we list specific news sources documenting school disruptions.

"21 dead, 37 missing in SW.China floods." China.org.cn, June 7, 2011. Accessed July 3, 2024. http://www.china.org.cn/china/2011-06/07/content_22724037.htm.

"2010 floods in Pakistan: Latest update." UK Government, accessed July 3, 2024. https://www.gov.uk/government/news/2010-floods-in-pakistan-latest-update.

"2019 Philippines Mindanao Island Earthquakes: Facts, FAQs, and How to Help." Save the Children, accessed November 7, 2022. www.savethechildren.org/us/what-we-do/emergency-response/philippines-earthquake-mindanao-island-2019-facts.

"27 Schools Closed Due to Increased Flooding on Malaysia's Eastern Coast." TODAY, accessed November 7, 2022. www.todayonline.com/world/asia/27-schools-closed-due-increased-flooding-malaysias-eastern-coast.

"ACT Alert India No. 33/2009 Chennai, Tamil Nadu: Heavy Rains." ReliefWeb, accessed July 3, 2024. https://reliefweb.int/report/india/act-alert-india-no-332009-chennai-tamil-nadu-heavy-rains.

"ACT Alliance Alert: Flooding in Orissa, India." Trust.org, accessed July 3, 2024. https://news.trust.org/item/20110916085200-2cy59/.

"Amid Acute Water Crisis, Schools in Shimla Shut for 5 Days." NDTV.com, June 2, 2018. www.ndtv.com/india-news/schools-in-shimla-closed-for-5-days-starting-monday-due-to-water-crisis-says-himachal-government-rep-1861557.

"Bangladesh: Over 4,000 Primary Schools Closed by Floods - Bangladesh." ReliefWeb, August 21, 2007. reliefweb.int/report/bangladesh/bangladesh-over-4000-primary-schools-closed-floods.

"Bengal Schools to Close for 11 Days Over Sudden Heat Wave - Times of India." The Times of India, accessed November 7, 2022. timesofindia.indiatimes.com/education/news/bengal-schools-to-close-for-11-days-over-sudden-heat-wave/articleshow/64639741.cms.

"Bihar flood takes a toll on education." India Today, October 8, 2008. https://www.indiatoday.in/web-exclusive/story/bihar-flood-takes-a-toll-on-education-31173-2008-10-08.

"Bolivia Schools Close Early as Drought Empties Reservoirs." BBC News, accessed November 7, 2022. www.bbc.com/news/world-latin-america-38073575.

"Chennai Water Crisis: School Closes Down for Junior Classes, Others Declare Half-day." The News Minute, June 19, 2019. www.thenewsminute.com/article/chennai-water-crisis-school-closes-down-junior-classes-others-declare-half-day-103919.

"Children During Long Winter Vacation in Central Highlands." UNICEF Afghanistan, February 17, 2019. www.unicef.org/afghanistan/stories/children-during-long-winter-vacation-central-highlands.

"China - Hong Kong: Flu Triggers Two-week School Closure - China - Hong Kong (Special Administrative Region)." ReliefWeb, June 11, 2009. reliefweb.int/report/china-hong-kong-s

pecial-administrative-region/china-hong-kong-flu-triggers-two-week-school.

"China Floods Information Bulletin N° 2." ReliefWeb, accessed July 3, 2024. https://reliefweb.int/map/china/china-floods-information-bulletin-n-2.

"China's Guangdong suspends school, traffic as typhoon nears." People's Daily Online, August 25, 2022. Accessed July 3, 2024. http://english.people.com.cn/n3/2022/0825/c90000-10139320.html.

"China's Hainan Island Hit by Typhoon." VOA News, September 29, 2011. Accessed July 3, 2024. https://www.voanews.com/a/typhoon-nesat-strikes-southern-chinese-island-130770678/145874.html.

"Chinese cities postpone school opening due to Typhoon Saola." People's Daily Online, August 31, 2023. Accessed July 3, 2024. http://en.people.cn/n3/2023/0831/c90000-20066025.html.

"Cyclone weakens into storm, keeps Mumbai on its toes." Indian Express, accessed July 3, 2024. https://indianexpress.com/article/news-archive/web/cyclone-weakens-into-storm-keeps-mumbai-on-its-toes/.

Daniele, Ushar. "Air Pollution in Malaysia Forces 400 School Closures, Sickens More Than 100 Children — CNN." CNN, June 27, 2019. www.cnn.com/2019/06/26/health/malaysia-pollution-schools-intl-hnk/index.html.

Davison, Tamara. "Schools to Close Due to Water Shortages in Mexico City - Aztec Reports." Aztec Reports, October 25, 2018. aztecreports.com/school-close-water-shortage/1801.

Deonath, Gabrielle. "Over 2M Afghan Children Have Given Up School For Work." Global Citizen, July 24, 2019. Accessed July 3, 2024. https://www.globalcitizen.org/en/content/afghanistan-child-labor/.

"Delhi Smog: Schools Closed for Three Days as Pollution Worsens." BBC News, accessed November 7, 2022. www.bbc.com/news/world-asia-india-37887937.

"Dhanusa Community Schools to Shut Down From Today." Kathmandu Post, November 7, 2022. kathmandupost.com/national/2018/01/03/dhanusa-community-schools-to-shut-down-from-today.

"Dry Pipes - Water Crisis Forces Schools to Close." Jamaica Gleaner, April 11, 2014. jamaica-gleaner.com/gleaner/20140411/lead/lead1.html.

"Ebola Outbreak: Nigeria Closes All Schools Until October." BBC News, accessed November 7, 2022. www.bbc.com/news/world-africa-28950347.

"EC Asks Govt to Shut Schools on May 8-14." Kathmandu Post, November 7, 2022. kathmandupost.com/national/2017/05/04/ec-asks-govt-to-shut-schools-on-may-8-14.

"Ethiopia Drought: Schools Closing, Livestock Dying, and Wells Drying Up." ReliefWeb, accessed July 3, 2024. https://reliefweb.int/report/ethiopia/ethiopia-drought-schools-closing-livestock-dying-and-wells-drying.

"Ethiopia: Activities and Resource Gap - Closed Schools in Drought-Affected Areas, Oromia." UN Office for the Coordination of Humanitarian Affairs, accessed July 3, 2024. https://www.unocha.org/publications/report/ethiopia/ethiopia-activities-and-resource-gap-closed-schools-drought-affected-areas-oromia.

"Flood water yet to recede, schools remain closed." The New Indian Express, July 25, 2014. https://www.newindianexpress.com/states/odisha/2014/Jul/25/flood-water-yet-to-recede-schools-remain-closed-639713.html.

"Flooding Forces School Closures in India's Hyderabad." Al Jazeera, September 24, 2016. https://www.aljazeera.com/gallery/2016/9/24/flooding-forces-school-closures-in-indias-hyderabad.

"Government Decides to Shut Schools for Four Days as Air Pollution Reaches Hazardous Levels." Kathmandu Post, November 7, 2022. kathmandupost.com/national/2021/03/29/government-decides-to-shut-schools-for-four-days-as-air-pollution-reaches-hazardous-levels.

"Hand, Foot and Mouth Disease Causes 18 Bangkok School Closures – Tasty Thailand." Tasty Thailand, July 17, 2012. tastythailand.com/hand-foot-and-mouth-disease-causes-18-bangkok-school-closures.

"Hand, Foot and Mouth Disease Outbreak in Malaysia: 6 Things You Need to Know About the Disease." The Straits Times, July 31, 2018. www.straitstimes.com/singapore/health/hand-foot-and-mouth-disease-outbreak-in-malaysia-6-things-you-need-to-know-about.

"Heatwave Shuts More Than 250 Malaysian Schools: Reports." phys.org, accessed November 7, 2022. phys.org/news/2016-04-heatwave-malaysian-schools.html.

"Heavy Rain puts life out of gear in Chennai and other parts of TN." ReliefWeb, accessed July 3, 2024. https://reliefweb.int/report/india/heavy-rain-puts-life-out-gear-chennai-and-other-parts-tn.

"Hong Kong flooded as torrential storms strike." China.org.cn, June 8, 2008. Accessed July 3, 2024. http://www.china.org.cn/china/flood2008/2008-06/08/content_15899315.html.

"How devastating floods in Thailand in 2011 harmed students' academic growth." The 74 Million, accessed July 3, 2024. https://www.the74million.org/article/analysis-how-devastating-floods-in-thailand-in-2011-harmed-students-academic-growth-and-what-lessons-we-can-use-in-confronting-learning-loss-during-the-pandemic/.

"India: Maharashtra and Goa Floods OCHA Situation Report No. 3." ReliefWeb, accessed July 3, 2024. https://reliefweb.int/report/india/india-maharashtra-and-goa-floods-ocha-situation-report-no-3?OpenDocument=&rc=3&emid=FL-2005-000104-IND.

"Indonesian children locked out of school as El Niño haze chokes parts of Sumatra, Kalimantan." Mongabay, October 2023. Accessed July 3, 2024. https://news.mongabay.com/2023/10/indonesian-children-locked-out-of-school-as-el-nino-haze-chokes-parts-of-sumatra-kalimantan/.

"Keeping children in school during natural crises." Stanford Social Innovation Review, accessed July 3, 2024. https://ssir.org/articles/entry/keeping_children_in_school_during_natural_crises.

"Malawi Drought 2015-2016, Post-Disaster Needs Assessment." PreventionWeb, accessed July 3, 2024. https://www.preventionweb.net/publication/malawi-drought-2015-2016-post-disaster-needs-assessment.

"Mers: South Korea Closes 700 Schools After Third Death." BBC News, accessed November 7, 2022. www.bbc.com/news/world-asia-33002795.

"Monsoon floods cripple NCR." The Manila Times, August 20, 2013. Accessed July 3, 2024. https://www.manilatimes.net/2013/08/20/todays-headline-photos/headlines/monsoon-floods-cripple-ncr-2/31316.

"Monsoon Rains Bring Severe Flooding and Landslides Across South Asia, Affecting More Than Five Million Children." UNICEF, November 3, 2022. www.unicef.org/rosa/press-releases/monsoon-rains-bring-severe-flooding-and-landslides-across-south-asia-affecting-more.

"More than 5,700 people relocated in flood-hit east China's county." People's Daily Online, July 7, 2016. Accessed July 3, 2024. http://english.people.com.cn/n3/2016/0707/c90000-9082842.html.

"More Than 10,000 Schools in Sichuan Badly Damaged." UNICEF China, May 18, 2008. www.un

icef.cn/en/press-releases/more-10000-schools-sichuan-badly-damaged.
"New Delhi schools close after monsoon floods kill at least 15, Pakistan on alert for more flooding." Kaler Kantho, July 10, 2023. Accessed July 3, 2024. https://www.kalerkantho.com/english/online/international/2023/07/10/54615.
"Pakistan: Flood Damaged Schools Lead to Education Worries - Pakistan." ReliefWeb, August 26, 2010. reliefweb.int/report/pakistan/pakistan-flood-damaged-schools-lead-education-worries.
"Philippines: Mayon Volcano - Jan 2018." ReliefWeb, July 3, 2020. reliefweb.int/disaster/vo-2018-000005-phl.
"Philippines: More than a million children missing out on education." Save the Children, accessed July 3, 2024. https://www.savethechildren.net/news/philippines-more-million-children-missing-out-education-thousands-schools-remain-shut-following.
"Pontianak Urges School Closure for Smog." The Jakarta Post, August 20, 2018. www.thejakartapost.com/news/2018/08/20/pontianak-urges-school-closure-for-smog.html.
"Prioritizing education in the face of natural disasters." Brookings Institution, accessed July 3, 2024. https://www.brookings.edu/articles/prioritizing-education-in-the-face-of-natural-disasters/.
"Prolonged Drought in East Africa Forces Millions of Children Out of School." Global Partnership for Education, April 3, 2018. www.globalpartnership.org/blog/prolonged-drought-east-africa-forces-millions-children-out-school.
Public Company Limited, Bangkok Post. "Cambodia Shuts Schools Amid Disease Fears." Bangkok Post, accessed November 7, 2022. www.bangkokpost.com/world/303151/cambodia-shuts-schools-amid-disease-fears.
Ramesh, Randeep. "Indian floods displace hundreds of thousands." The Guardian, August 10, 2006. Accessed July 3, 2024. https://www.theguardian.com/world/2006/aug/10/india.randeepramesh.
ReliefWeb. "Bangladesh Humanitarian Response and Recovery Plan: Monsoon Floods, August 2019." ReliefWeb, accessed July 3, 2024. https://reliefweb.int/report/bangladesh/bangladesh-humanitarian-response-and-recovery-plan-monsoon-floods-august-2019.
ReliefWeb. "Bangladesh Monsoon Flood 2019 Situation Report #1, 13 July 2019." ReliefWeb, accessed July 3, 2024. https://reliefweb.int/report/bangladesh/bangladesh-monsoon-flood-2019-situation-report-1-13-july-2019.
"Schools and colleges affected by Uttarakhand floods." ReliefWeb, accessed July 3, 2024. https://reliefweb.int/report/india/schools-and-colleges-affected-uttarakhand-floods.
"Schools Closed by Ebola Reopen in Guinea and Liberia." VOA, February 12, 2015. learningenglish.voa-news.com/a/schools-reopen-guinea-liberia-closed-ebola/2635017.html.
"Schools in Fiji Will Stay Closed Until Cleared." FijiTimes, January 17, 2022. www.fijitimes.com/schools-in-fiji-will-stay-closed-until-cleared.
"Schools out: Pakistan floods raise education fears for millions." Context News, accessed July 3, 2024. https://www.context.news/climate-risks/schools-out-pakistan-floods-raise-education-fears-for-millions.
"Schools to be closed in Chittagong on Tuesday due to heavy rain." Dhaka Tribune, accessed July 3, 2024. https://www.dhakatribune.com/bangladesh/nation/321854/schools-to-be-closed-in-chittagong-on-tuesday-due.
"Situation Report: Uttarkashi, August 4, 2012." ReliefWeb, accessed July 3, 2024. https://reliefweb.int/report/india/situation-report-uttarkashi-4-aug-2012.

"Solar-powered floating schools in Bangladesh." Designboom, accessed July 3, 2024. https://www.designboom.com/technology/solar-powered-floating-schools-bangladesh/.

"South Asia floods kill 1,200 and shut 1.8 million children out of school." The Guardian, August 31, 2017. Accessed July 3, 2024. https://www.theguardian.com/world/2017/aug/30/mumbai-paralysed-by-floods-as-india-and-region-hit-by-worst-monsoon-rains-in-years.

"Storms threaten China's coastal provinces." China.org.cn, September 1, 2010. Accessed July 3, 2024. http://www.china.org.cn/environment/2010-09/01/content_20837052.htm.

"Sudan schools crisis threatens grim future for children." France 24, accessed July 3, 2024. https://www.france24.com/en/live-news/20221013-sudan-schools-crisis-threatens-grim-future-for-children.

"Taiwan cleans up as Typhoon Fanapi reaches China." BBC News, September 19, 2010. Accessed July 3, 2024. https://www.bbc.co.uk/news/world-asia-pacific-11360782.

"Thai Capital to Close 435 Schools to Halt H1N1 Spread." Reuters, accessed November 7, 2022. www.reuters.com/article/idUSBKK452436.

"Thousands of Schools Destroyed, Damaged or Disrupted by South Asia's Deadly Floods." Theirworld, August 15, 2022. theirworld.org/news/south-asia-floods-destroy-damage-thousands-schools-india-bangladesh-nepal.

"Three Million Children Affected by Mindanao Earthquakes: Save the Children Deploys Rapid Response Team to Meet the Needs of Communities - Philippines." ReliefWeb, November 1, 2019. reliefweb.int/report/philippines/three-million-children-affected-mindanao-earthquakes-save-children-deploys-rapid.

"Typhoon Glenda shuts down Metro Manila." The Manila Times, July 16, 2014. Accessed July 3, 2024. https://www.manilatimes.net/2014/07/16/todays-headline-photos/top-stories/typhoon-glenda-shuts-down-metro-manila/111972.

"Typhoon Hato kills 12 in Macau and southern China." The Guardian, August 23, 2017. Accessed July 3, 2024. https://www.theguardian.com/world/2017/aug/23/typhoon-hato-hong-kong-battens-down-as-storm-closes-schools-and-stock-market.

"Typhoon Kalmaegi hits southern China." BBC News, September 16, 2014. Accessed July 3, 2024. https://www.bbc.co.uk/news/business-29217218.

"Typhoon Khanun hits China." Al Jazeera, September 12, 2005. Accessed July 3, 2024. https://www.aljazeera.com/news/2005/9/12/typhoon-khanun-hits-china.

"Typhoon Utor Hits Hong Kong, Southern China." VOA News, August 14, 2013. Accessed July 3, 2024. https://www.voanews.com/a/typhoon-utor-hits-hong-kong-southern-china/1729488.html.

"Ulysses' floods shut down Metro Manila." The Manila Times, November 13, 2020. Accessed July 3, 2024. https://www.manilatimes.net/2020/11/13/news/headlines/ulysses-floods-shut-down-metro-manila/795623.

"UN aid for typhoon victims." DW, December 9, 2012. Accessed July 3, 2024. https://www.dw.com/en/un-appeals-to-send-aid-to-filipino-typhoon-victims/a-16440806.

UNICEF. "UNICEF Afghanistan Humanitarian Situation Report, January-December 2021." ReliefWeb, accessed July 3, 2024. https://reliefweb.int/report/afghanistan/unicef-afghanistan-humanitarian-situation-report-january-december-2021.

UNICEF. "UNICEF Bangladesh Humanitarian Situation Report No. 8: North-eastern Flood, 4 August 2022." ReliefWeb, accessed July 3, 2024. https://reliefweb.int/report/bangladesh/unicef-bangladesh-humanitarian-situation-report-no-8-north-eastern-flood-4-august-2022.

"UNICEF Ethiopia Country Office Humanitarian Situation Report No. 1, January 2022." Re-

liefWeb, accessed July 3, 2024. https://reliefweb.int/report/ethiopia/unicef-ethiopia-country-office-humanitarian-situation-report-no-1-january-2022.

"UNICEF Executive Director visits drought-affected Ethiopia." UNICEF, accessed July 3, 2024. https://www.unicef.org/press-releases/unicef-executive-director-catherine-russell-visits-drought-impacted-somali-region.

"UNICEF Humanitarian Action Update South Asia floods 25 Sep 2007." ReliefWeb, September 25, 2007. Accessed July 3, 2024. https://reliefweb.int/report/bangladesh/unicef-humanitarian-action-update-south-asia-floods-25-sep-2007.

"Unraveling the Water Crisis in Venezuela." Center for Strategic and International Studies, May 27, 2021. www.csis.org/analysis/unraveling-water-crisis-venezuela.

"Uttarakhand floods: eight killed in heavy rains, schools closed till Thursday." NDTV, accessed July 3, 2024. https://www.ndtv.com/india-news/uttarakhand-floods-eight-killed-in-heavy-rains-schools-closed-till-thursday-464671.

"Vanuatu: Tackling the Impact of Natural Disasters by Building a Resilient Education System — Global Partnership for Education." Global Partnership for Education, accessed November 7, 2022. www.globalpartnership.org/results/stories-of-change/vanuatu-tackling-impact-natural-disasters-building-resilient-education.

Watts, Jonathan. "China hit by strongest typhoon for half a century." The Guardian, August 11, 2006. Accessed July 3, 2024. https://www.theguardian.com/environment/2006/aug/11/china.naturaldisasters.

"Water Crisis Forces Lyantonde Schools to Close Prematurely:: Uganda Radionetwork." Uganda Radionetwork, accessed November 7, 2022. ugandaradionetwork.net/story/water-crisis-forces-lyantonde-schools-to-close-prematurely.

"Worst flooding in decades." The New Humanitarian, October 10, 2012. Accessed July 3, 2024. https://www.thenewhumanitarian.org/news/2012/10/10/worst-flooding-decades.

"Zimbabwe: Stronger education system after Cyclone Idai." Global Partnership for Education, accessed July 3, 2024. https://www.globalpartnership.org/results/stories-of-change/zimbabwe-stronger-education-system-after-cyclone-idai.

"Zimbabwe: Water Shortages Force Schools to Close - Zimbabwe." ReliefWeb, September 10, 2004. reliefweb.int/report/zimbabwe/zimbabwe-water-shortages-force-schools-close.
