# Supplementary material for: Mobile education builds resilience during shocks in five countries
Source: Nature. 2026 Sep 9;657(8132):689–96. doi: 10.1038/s41586-026-10990-x (PMC13581608; doi:10.1038/s41586-026-10990-x)
Supplement: Supplementary file 3 [file 41586_2026_10990_MOESM3_ESM.pdf]

nature portfolio

# Peer Review File

# Mobile education builds resilience during shocks in five countries

Corresponding Author: Dr Noam Angrist

Any redactions in this file are there to maintain patient confidentiality, the confidentiality of unpublished data, or to remove third-party material.

**This file contains all reviewer reports in order by version, followed by all author rebuttals in order by version.**

**Attachments originally included by the reviewers as part of their assessment can be found at the end of this file.**

Version 0:

Reviewer comments:

Referee #1

(Remarks to the Author)
Comments are provided by Salman Asim:
Senior Economist, Education Global Practice, World Bank

Summary

This is a technically strong and ambitious paper, coordinating five RCTs across countries during COVID with clean randomization, low attrition, and credible ITT estimates. The main results show that phone tutoring can offset steep learning losses, but the large effect sizes are better understood as averting learning losses rather than transformative gains. Claims of broad generalizability are overstated: the typhoon evidence and wide variation in impacts across countries (India (0.22 SD), Nepal (0.32 SD), and Uganda (0.89 SD) underscore that effects are highly contingent on crisis conditions and local context. The intervention is promising as an emergency response option, but it should not be framed as a universal blueprint for resilient education systems.

Overall, this is a highly valuable contribution, and I believe with more modest and precise framing, it will make an even stronger impact in the policy and research community.

Detailed Comments

Angrist et al. study claims phone-based tutoring is a low-cost, scalable, and resilient model that sustained learning gains across diverse emergencies, with effect sizes (0.3–0.35 standard deviations) far larger than typical education interventions. The key highlights of the paper are: (1) the authors claim that findings are generalizable across contexts and modality of delivery (NGO vs Government teachers); (2) it is a cost-effective intervention at an average cost of about $11/child; (3) the gains held even during a major post COVID emergency (i.e. typhoon in Philippines).

Internal and External Validity of the Claims/Treatment Fidelity

The paper is exceptionally strong in setting up the RCTs. Treatment assignment was clearly randomized across multiple countries, with transparent documentation of the unit of randomization (household, grade, or school) and appropriate clustering of standard errors. The ITT estimates are well justified and consistently applied, and attrition was low (about 20%) with no evidence of differential attrition across arms. Causal claims within the sampled, phone-reachable populations are therefore highly credible. Internal validity is further reinforced by high engagement rates (70–80%) that limit concerns about selective compliance, by the replication of impacts across five countries, and by the embedded randomization of NGO

versus government implementers in Nepal and the Philippines, which allows for clean causal inference on delivery models.

My reading of the paper is less sympathetic when it comes to the external validity claims. What the study does convincingly show is that interactive phone tutoring worked for the sampled populations across multiple countries and delivery channels in the time of COVID, or more generally when schools were closed. Where I think the paper overreaches is in presenting this as a universally generalizable resilient systems solution.

The main issue is that any intervention capable of sustaining steady-state school learning during closures could have generated large relative effects. There is little in the results that suggested phone tutoring per se, rather than the mere continuation of structured learning that drove the outcomes. Indeed, the reported 0.3–0.35 SD effect sizes line up with what we already know about pandemic learning losses. In my own longitudinal work in Malawi (Asim et al. 2024), students had lost about 0.5 SD by the time schools reopened after seven months, and these losses compounded to nearly 0.8 SD within the following half year as the gradient of learning slowed. By comparison, the Angrist et al. results suggest that tutoring helped children recover roughly half of what they would have learned in steady-state schooling — a valuable result, but not evidence of a transformative intervention.

Framed this way, the program is promising as an emergency response tool among others, but not yet proven as a generalizable model for all emergency situations. Unless the authors can show that the effects exceed the magnitude of lost learning in these countries, or that phone tutoring clearly outperforms credible alternatives such as TaRL-style community camps, the claim that this is the most effective or scalable solution feels overstated. A more balanced framing would recognize it as one plausible approach to mitigating learning collapse in crises rather than a universal blueprint for a resilient education system. It would also help if the authors benchmarked their results explicitly against pre-COVID annual learning gains in these contexts and situated their findings alongside other crisis-response interventions. Finally, I would encourage them to discuss more openly how the choice to standardize scores at the control endline shapes interpretation. In a setting where control groups are collapsing, these scaling choices are not trivial, and robust checks against baseline or pooled SDs would give readers more confidence in how to interpret the magnitude of the results.

Originality/Significance of Contributions

The real strength of the paper lies in its scale and execution — coordinating five RCTs across countries and delivery channels under COVID is impressive and goes further than most education trials I have reviewed. The authors should feel accomplished in having delivered a well-executed set of trials, but they need not exaggerate the claims. In my view, the underlying idea is not novel: the model builds on the well-established principle of targeted instruction popularized by TaRL, though the authors adapted it to a phone-based, one-on-one format during school closures. The real significance is in showing that such a model can be implemented at scale (though it is important to note that the India Karnataka rollout is only quasi-experimental), at relatively low cost, and in COVID closure conditions with reasonably consistent results. Framed this way, the contribution is valuable, but more modest than the current narrative suggests.

On significance, the interpretation is more fragile than the narrative suggests. The apparent consistency of results is in large part an artefact of the crisis context — the study was conducted in regions like South Asia and Sub-Saharan Africa where school closures were unusually long and learning losses particularly steep (Patrinos et al.). In such settings, any intervention that sustained even modest levels of learning would show large relative effects. Moreover, the cross-country estimates in the paper are far from uniform: Uganda reports an effect close to 0.9 SD, the Philippines about 0.45 SD, while India, Kenya, and Nepal are closer to 0.3 SD. This spread in magnitudes raises important questions about why an intervention framed as highly effective yielded such different impacts across countries. It suggests the results are less universally stable than implied and remain heavily contingent on local conditions and the depth of learning collapse.

Interpretation of Results/Robustness

In Section VI.E the authors present an external validity analysis by testing whether conditional treatment effect estimates from one country can predict impacts on another. Their own results show the opposite of what their narrative suggests: extrapolations fail badly. For example, extrapolating from Uganda's 0.89 SD effect produces implausibly high predicted impacts in other countries, while India's more modest 0.22 SD effect is mis-predicted by large margins — in some cases even yielding negative expected effects, such as India to Nepal (-0.07 SD). The clear finding is that baseline covariates cannot explain treatment heterogeneity, and that treatment effects do not transport cleanly across contexts.

This validity exercise needs to be interpreted for what it shows: the intervention's impacts are highly contingent on local contexts and cannot be assumed to generalize. If anything, the stress test undercuts the broad claims of consistency and resilience — it highlights the fragility of the results across settings. The true policy-relevant takeaway is sobering: a policymaker cannot be sure what impact to expect in their own context. And if that is the case, how can the intervention be generalized as a blueprint for education emergencies? The only claim that seems defensible is narrower: in contexts where learning collapses as it did under COVID, phone tutoring can help recover a portion of the lost ground.

Referee #2

(Remarks to the Author)
This paper evaluates a remote tutoring program to maintain student learning during breaks in schooling. Breaks may be due to emergencies like the COVID-19 pandemic, natural hazards like a typhoon in the Philippines, or even school vacations.

The authors test two delivery mechanisms in a multisite RCT in 5 countries: SMS messages and phone calls. They find that the program, especially the phone calls, leads to large increases in learning measured by correct answers to a series of progressive questions. Several features of the implemetation and evaluation design allow this paper to make important contributions to the education literature and also to a broader literature on the promises and limitations of RCTs as tools for learning about policies.

First, and most obviously, the impressive scale of data collection across five countries allows us to learn what works in different contexts.

At the same time, the degree of control and attention to detail across the five sites seems noteworthy for a multisite RCT. This control reduces undesirable variation in program implementation and gives us some insight into where differences are coming from. My favourite example of this is captured in Figure A4, which shows the percentage of instruction that was targetted in the different sites. Having a standard shared protocol that defines and measures targetting in the same way across sites, and collecting this type of data on intermediate stages of implementation, allows the authors to establish that there is a correlation between the extent of targetting and the size of the treatment effect. This increases the plausibility that targetting is an important mechanism as they theorise.

Several other design details deserve merit: randomising whether the implenters are from NGOs or government in Nepal and the Philippines addresses questions about whether RCT effect sizes are only due to motivated implementers; randomising off a list of eligible teachers in Nepal gives us some insights into the question of whether program impacts go through teacher selection or the program itself; multiple checks and repeated measurements increase confidence in the assessment tools.

Major comment 1:
The biggest weakness of the paper is the external validity analysis presented in section VI.E. The authors present it as an analysis that highlights the benefits of a multisite RCT over the state of the art. However, it's so perfunctorily done that I don't find it very useful. I don't have the impression that many people following the state of the art would expect covariates that do not affect treatment effects in one setting to be useful predictors of treatment effects in another setting. So the finding that baseline learning, gender and student grade—which are not associated with any treatment heterogeneity (Table A6)—are not useful covariates for predicting effects in other sites is not very insightful.

In fact, going back to the Hotz, Imbens and Mortimer (2005) paper cited, the original authors raise at least two reasons why sites might have different treatment effects. "First, the distribution of characteristics in the two populations may differ. If [a characteristic] is associated with the outcomes of interest and in particular (emphasis mine) with program efficacy, average program effects may be found to differ for the two programs....The second reason that treatment effects may differ across programs is that the programs, even if nominally the same, are heterogeneous in their components...[or]...may differ qualitatively."

The second reason, in light of the evidence the ConnectEd authors present on targetting, seems to be the most relevant. It seems that engaging with that aspect of the external validity literature is likely to yield more productive insights.

Major comment 2:
The statistical analysis is competently done. I could find most measures of uncertainty I searched for and p-values, confidence intervals and standard errors are appropriately presented. I would only request additional information on the multiple hypothesis corrections presented in Table A9. How many hypotheses are being corrected for? Is it 5 for the different countries; 2 for the different treatment arms or 5 x 2 for the interaction between them?

It would also be helpful to see the Karnataka evaluation at scale results presented in similar standard deviation units as the main results. That would make comparisons easier.

Major comment 3:
The paper is very well written, but I still had some questions about implementation details that I couldn't quickly answer. First, for each site, do we know whether children were in school or not? Figure A7 shows that some schools were partially closed at the national level, but what does that mean for the actual children and families who were enrolled in the program? It's likely that some of the enthusiasm for the program was driven by children, families, and teachers who had no alternatives. Essentially, I would like to see the data from question 10 of the baseline and endline questionnaires presented. It would be helpful to interpret results in light of how the tutoring was interacting with any, likely limited, schooling opportunities.

Second, what explains the differences in implementation lengths (Table A8)? Were there differences in the 'doses' of the program?

Some of this might be addressed by slighly re-writing the country context subsections in III.D so that each country sub-section explicitly follows the exact same structure. This would allow a reader to more easily grasp the peculiarities of each implementation.

Referee #3

(Remarks to the Author)
Review of Nature MS 2025-07-17892

OVERVIEW

This is a well-written paper on an ambitious set of experiments that aimed to address the educational emergency caused by school closures during the COVID-19 pandemic. The authors document the effect of phone-based tutoring and, with less emphasis, SMS-based educational content delivery on learning outcomes of students in primary grades in five distinct settings (Kenya, India, Nepal, Philippines and Uganda), based on a model that had previously been developed and evaluated in Botswana (Angrist et al, Nature Human Behaviour 2022). Using short phone-based assessments, they document impressive effect sizes on student achievement of ~0.3-0.4 standard deviations (s.d.) after about 8 weekly lessons of 20-minutes each. These aggregate estimates hide substantial heterogeneity ranging from between 0.085 s.d. in Kenya to 0.89 s.d. in Uganda for the same intervention. The authors make the case that the program, and the evidence, are both relevant beyond the COVID-19 pandemic, with applicability also to other emergencies that occur periodically in many parts of the world — they provide suggestive evidence from Philippines and India to this effect. In specific settings, the authors also test some sub-randomizations (such as NGO versus government delivery) but these are secondary to the main focus of the trials.

SUMMARY OF COMMENTS

There is a lot to like in this paper. I especially appreciated both the immense efforts of the authors in setting up the core intervention in multiple countries, with multiple partners in the challenging setting of the pandemic, as well as the commitment and flexibility to subject each iteration of the program to a randomized control trial. The core result — that the composite phone-based tutoring intervention was effective in raising achievement — is clear and unlikely to be sensitive. Changes I recommend below are mostly expositional and not primarily centered on the scientific validity of the authors' claims (although they matter for clarity and transparency).

To my mind, it is clear that this paper deserves to be published in a reputed general interest journal. The specific need that the intervention addresses (remote instruction for foundational learning in low/middle-income settings) is widespread and, as the authors demonstrate, the specific intervention can also be adapted across settings. These authors were at the forefront of the literature on remote instruction during COVID-19 and, although there were several contemporaneous experiments (including e.g. a 7-country study in Latin America, plus others in Spain, Italy, India etc.) with mostly positive effects, I think they can legitimately claim to be the leaders in that space. There is some policy momentum now behind exploring remote tutoring outside of COVID-19 settings, including the scale-up discussed in this paper, and I think at least some of this reflects the dissemination of the research discussed in this paper. So, it is clear that this stream of work has been influential.

Whether the specific scientific innovation is adequate for Nature, and whether the paper represents a sufficient advance over the knowledge in the first experiment in Nature Human Behaviour (Angrist et al, 2022) and the many other contemporaneous studies, is a decision for the Editor and I will not have comments in that direction. My comments below focus on expositional changes that, in my view, are essential for the paper to send out a clear actionable message for a broad readership. That being said, the Editor would also be in a much better position to judge the requirements for the Nature readership than a typical Economics paper.

MAIN COMMENTS

#1 Reduce the number of reported analyses
The paper would be much better served if it narrowed down the scope. While I appreciate the effort the authors took across multiple distinct empirical analyses, they are not equally important and much of the analysis distracts from their main points.

To my mind, the paper has three main results: (i) phone-based tutoring support was effective in raising student achievement in very diverse settings, (ii) SMS-only interventions were typically not effective and (iii) there is huge heterogeneity in treatment effect sizes across contexts and experiments, which is not explained by measured sources of variation. Everything else is a distraction. The paper would be well-served in only highlighting these.

In particular, I recommend that various sub-treatments (govt vs. NGO), the Philippines typhoon subsection, the analysis of the India scale-up (which is non-experimental, and is half-hearted by the standards of high-quality non-experimental causal evaluations) etc. should all be removed from this draft. So should the validation of the phone survey in Kenya. These are not the features that lead this paper to be of general interest, nor are they necessarily the most robust parts of the analysis.

#2 Explain the core experiment design and results more clearly
Getting rid of secondary analyses, and associated exhibits, also frees up space for the authors to expand their exhibits to provide useful insight about the actual core experiments.

There are two issues here that I think are first-order importance:

a) Details of experiment design:

Move Table A.8 to the main body of the paper. This is not appendix material.
Please also indicate the number of treated and control individuals in each experiment separately, as well as details of attrition (for each experiment). I recommend a full CONSORT style diagram for each of the 5 experiments as supplementary material.

Please also discuss the representativeness (or not) of the population compared to the broader school-going population in each of these countries. It is not clear from the draft how the sampling frame was identified, how households were approached and whether there were extra selection criteria for the study before the randomization. I suspect there were some such criteria since I note that the response rates for the surveys are considerably in excess of those found by other research teams from lists of phone numbers in developing countries during COVID-19.
I note that in the Declaration accompanying the submission, the Corresponding Author confirms that the paper reports "the exact sample size (n) for each experimental group/condition, given as a discrete number and unit of measurement " (emphasis mine). I apologize if I have missed it but I did not find this information. Specifically, I cannot on the basis of Section III. C, Section III.D or Appendix Table A.8 answer just how many students were randomized to be treated in each of the individual experiments? In the countries which featured further sub-randomization (e.g. NGO vs. government implementation), I could not tell whether these were randomized with equal probability or not. As a summary measure, I cannot tell here what the total number of treated individuals across the 5 studies was. These are crucial details for interpreting this experiment, and also for comparing with the many other studies featuring remote instruction. The authors should include this in the main body of the paper.
Please note that, while I classify these comments as expository, issues of sampling, representativeness and attrition for each experiment individually go to the heart of the scientific validity of the claims (and the implications for policy). It is essential that this is fully specified in at least the supplementary material for readers.

b) Transparency in presentation of results across experiments

The greatest strength of this study is the running of 5 distinct experiments across settings. Given this, I do not understand the choice to present results in the aggregated (pooled) sample as the main specification.
As I see it, your main result is Table 4 and that should be your key exhibit.
The second table that should be emphasized as the "main" result is Table 5 but with a separate panel for each experiment. Please do not pool across experiments in this manner, especially given different settings, sampling protocols, implementation instructions and randomization procedures — doing this properly would require very different Bayesian approaches (as Meager (2019, AEJ:Applied) demonstrated for experiments on microfinance). In that table, please also have the baseline value and the control mean for each individual country sample in each panel.
This is very important since we do not have any indication in the manuscript about differences in baseline achievement across your samples (i.e. differences between e.g. Philippines and Kenya, not the treated and control groups within each country). Given the coarse categorization that is possible with these short ASER type assessments, it is possible that much of the difference across experiments comes from a "standard deviation" being very different across each of the individual samples. It would be far more transparent to present actual proficiency gains in the 5 samples separately. Formatted suitably, this could be the key exhibit of the paper, easily contained in a single (full-page) exhibit.
This also would be a more principled way of dealing with the coarseness of the ordinal ASER-type measures. For an ordinal variable that only takes 4-5 categorical values, the standard deviation metric seems particularly difficult to interpret. This is even more so when comparing across vastly different contexts. I understand that you have to report the z-score results somewhere but it would be far more informative to see the competence-level results than the aggregated scores.

I would recommend dropping the section of scale up in India entirely. It detracts from the core of the paper and does not speak directly to the rest of the paper — I suspect the authors agree, which is why the entire section only leads to Appendix exhibits and not a single exhibit in the main body of the paper.
Incidentally, the issues of ordinality are especially a concern with the difference-in-difference approach in the analysis of the scale-up. Analysis of changes only makes sense with an implicit assumption of cardinality: a change from 1 to 2 has to equal the change from 3 to 4. This would be okay if this scale was approximately equal-interval but it is not. Ordinality is, of course, a broader problem when it comes to test scores (see, e.g., Bond and Lang 2013) but it is especially severe for short assessments that only permit a relatively coarse categorization.
It is possible, given the data, to write a much more compelling non-experimental evaluation of the scale-up. However, the space it would take to do this well is not feasible in the current paper. Nor do I think it adds to the clarity of the paper to have that also tacked on without doing full justice.
For transparency, I would also recommend being upfront about whether any of the analyses were pre-registered or not. Please note that I do not think there is anything fishy here – the analyses and outcomes are pretty standard but I suspect it is worth clarifying this in the draft as such pre-registration has moved to being increasingly common in the field. I don't think anybody would object to the lack of a full PAP while trying to coordinate a multi-country effort in the midst of COVID (when I'm sure you had very little ex ante knowledge of how well implementation would occur). I suggest this only for transparency.

#3 [OPTIONAL] Consider a crisp, short paper

This is purely a suggestion, offered with free disposal. In my view, the signal achievement of this project is to have conducted 5 experiments with multiple partners across countries in the midst of a severe education crisis. Communicating the very positive results from this endeavour should, in my view, be the single purpose of this paper.

For this single purpose, it is adequate to just report on the design of the core experiments and the main results on learning outcomes. Effectively, that means three tables: (i) to show sample characteristics and balance; (ii) to show effects on the aggregated standard deviation metric (currently Table 4); and (iii) to show effects on individual competences (Table 5, but by country). Everything else should go to supplementary materials.

If Nature allows, I would recommend a well-fleshed out Discussion section where you can discuss relevance to (i) other emergencies, (ii) post-COVID FLN, (iii) scale-ups, (iv) mechanisms etc. But these are NOT the main value-added of this

paper or this effort.

Given that the paper is in consideration for a broad readership, much beyond just economists, I think a crisp 5000 word paper with 3-4 exhibits would be far more influential in the world than a bloated paper with too many secondary analyses written for an Economics audience.

Referee #4

(Remarks to the Author)
Comments "Building Resilient Education Systems: Experimental Evidence Across Five Countries"
The paper presents causal evidence from an educational intervention that utilized SMS messages and calls with a teacher or tutor. The intervention took place in five different countries during disruptions to the school year, primarily due to school closures during COVID-19. It focused on mathematics, typically targeting grades 3-5, and employed regular teachers and tutors. The intervention was implemented in some locations through an NGO and in others via public schools. It employed random assignment, allowing for the identification of causal effects. The paper shows evidence of positive effects on a five-question math test (simple operations). Additionally, the paper presents tests for external validity using country-based covariates, explores the modality of delivery (public versus NGO), assesses individual countries, among other analyses. There is a lot to like about the paper. It is very impressive how the authors have polled together all these individual experiments.
Comments
• Please clearly explain the theory of change. My recommendation is to use the framework of Nickow, Oreopoulos, and Quan (on tutoring) and adapt it to the different design characteristics of this program.
• An important discussion point is the description of the counterfactual: what did the control group receive? Did they have instruction? Who provided that instruction—was it the same teacher as in the intervention? Did they meet with a professor regularly? Etc. The description of what happens with the control group is critical for understanding the effect size.
• I think a better description of the treatment and its interaction with "regular" schooling is important. Similar to the control, it is important to understand what the treatment group received from the school. Did they meet regularly with the "regular" teacher? Was it the same person delivering the tutoring? Did they only meet once per week for 20 minutes?
• The effect sizes are quite striking, considering the low dosages, the fact that it was during COVID, and the fact that it was implemented without major training for teachers/tutors. For example, an effect size of 0.891 SD (!) in Uganda is extremely high. One explanation for the effect sizes lies in the metric of the outcome. The outcome is a five-question test in a very narrow domain.
o Please present the distribution of the test. You standardized the test—please show the graph of the standardized tests.
o Please present the correlation of these five questions with a more meaningful test, either a national assessment or an international test.
• Relatedly, how do we know that the teachers/tutors did not teach to the test? By this, I mean that rote memorization can produce this type of effect. Can you provide evidence against this?
• On the intervention:
o Was the intervention conducted via the parents' phones? If so, how many phones per family? Is it credible that the parents would lend the phone to students for the duration of the intervention?
o At what time of the day were the phone calls made? The timing of the phone calls has been shown to have a large impact on these types of interventions.
o Exactly how was the "teaching at the right level" implemented? The domain of the intervention—math, specifically the four operations—does not align well with TaRL. If a student did not grasp summation, would the tutor continue focusing solely on summation? Did the tutor vary the level of summation (e.g., one-digit versus two-digit summation)? How did tutors record the progress of students?
o The students were in grades 3 to 5: were the content and level of tutoring the same for all?
o The paper claims that one of the "mechanisms" is "Platform: reach at the right level." For me, it is unclear what this means. The implementation uses phones, and the actual phone calls are flexible. However, this seems more about implementation than a mechanism. My suggestion is to reframe that discussion.
o Was there training provided for teachers/tutors?
• I would like to see a discussion about the initial level of students and whether the intervention was able to restore students to pre-COVID levels. In other words, was the intervention able to "increase" learning or just to recover lost learning?
• I believe that the discussion of "Results from Another Education Emergency" is not particularly interesting. My suggestion is to move that to an appendix and mention it briefly in a few lines.
• On the teachers:
o What is the complementarity between tutors and teachers? I asked before, but let me reiterate: were the same teachers delivering the program as those who provide regular teaching?
o The table of teacher practices shows self-assessed metrics. For instance, 76.9% of teachers in the control claim that they "target to student level." We know that this is not the case. Precisely all the agenda of TaRL is based on this false assumption. Similarly, involving parents is costly, and we know that teachers would prefer not to engage parents. I don't have a clear direction with this comment, except to temper expectations regarding the results.
Small comments:
• The 222 million children with disruptions: are they counted per year?
• Page 18: "The follow-up rate was very high at around 80 percent of respondents at baseline." That is quite a standard response rate; it is not actually "very high." Moreover, an attrition rate of 20% is quite serious.
• In the section "A Follow-On Evaluation at Scale," you don't need the first two equations ($\Delta Y0$ and $\Delta Y1$).

Version 1:

Reviewer comments:

Referee #1

(Remarks to the Author)
I appreciate the substantial revisions made in response to the review. The updated manuscript now presents a considerably more nuanced discussion of external validity, heterogeneity across contexts, and the role of alternative resilience approaches.

I also welcome the decision to move standardized outcomes out of the main tables and to report non-standardized learning measures more prominently. Finally, I appreciate the removal of Section VI.E, which I found difficult to reconcile with the broader interpretation of the results.

While I continue to believe the magnitude of the effects should be interpreted in light of unusually severe learning disruptions during COVID, the revisions have substantially improved the balance between the evidence presented and the claims made. Overall, I believe the manuscript has been materially strengthened!

Salman Asim
Senior Economist, Educaiton Global Practice, World Bank

Referee #2

(Remarks to the Author)
Thank you for the thorough review. The paper is streamlined and a pleasure to read. The additions and re-organisation improve clarity of what I see to be the key ideas.

My remaining comments are minor, and with some of them, I realise I am veering into the role of an unasked for copy-editor, so please consider them as suggestions rather than points that must be addressed.

1. I like the changes to section III and the discussion added to VI.B. They both help to give a clearer idea on the mechanisms that lead to the effects, and suggest some explanations for the heterogeneity we observe. I wonder if the two sections could be better integrated with each other. For example, by merging them into a single section after the results. On the other hand, I did not find Figure A6 very helpful. The paper mostly discusses "Tutoring inputs" (in the two sections I referenced) and does not say much about "Mechanisms of impact". I found it distracting to see a list of "Mechanisms of impact" listed with little idea of how they translate to the context.

2. I think Table A7 is reporting differences in means between the teachers randomised into the program and those who were not assigned to the program. If that is the case, I would prefer that is stated explicitly rather than saying "this table reports the balance..." which could mean different things in different settings.

3. Footnote 4 seems unnecessary as a paragraph-length footnote in the introduction. The material discussed in it is repeated in Section IV at the bottom of page 17. It is better placed there.

Referee #3

(Remarks to the Author)
I have now re-read the revised manuscript, as well as the authors' response to reviewers and the supplementary data appendixes.

I congratulate the authors on a thorough revision, and even more on having pulled off this very ambitious project in the midst of a global pandemic.

I have no further comments to offer and, if the Editor and other referees permit, hope very much to see this appear prominently in Nature after any changes needed to conform to Nature formats/guidelines.

Referee #4

(Remarks to the Author)
I read the second revision of the article. I believe that the authors addressed all my comments in a satisfactory manner, especially the comments about the theory of change and the counterfactual. I believe that the paper is now greatly improved. The fact that the authors streamlined the results and focused on fewer treatments and experiments (leaving aside, for instance, the quasi-experiment in India) makes the paper more readable. M=

**Open Access** This Peer Review File is licensed under a Creative Commons Attribution 4.0 International License, which permits use, sharing, adaptation, distribution and reproduction in any medium or format, as long as you give appropriate credit to the original author(s) and the source, provide a link to the Creative Commons license, and indicate if changes were made.
In cases where reviewers are anonymous, credit should be given to 'Anonymous Referee' and the source.
The images or other third party material in this Peer Review File are included in the article's Creative Commons license, unless indicated otherwise in a credit line to the material. If material is not included in the article's Creative Commons license and your intended use is not permitted by statutory regulation or exceeds the permitted use, you will need to obtain permission directly from the copyright holder.
To view a copy of this license, visit https://creativecommons.org/licenses/by/4.0/

Building Resilient Education Systems: Experimental Evidence Across Five Countries

***Response to Nature editor and reviewers***

Your manuscript, "Building Resilient Education Systems: Experimental Evidence Across Five Countries", has now been seen by four referees, whose comments are attached below. While they find your work of potential interest, as do we, they have raised important concerns that in our view need to be addressed before we can consider publication in Nature.

Thank you for your consideration for publication in Nature. We have now addressed all editorial and reviewer comments and are happy to share the updated manuscript. We agree that the updated paper is sharper, clearer, and more compelling – thank you to the editor and reviewers for a constructive set of comments.

For ease of review, we have left some of the original style, but we have also started to incorporate Nature formatting as requested, for example, focusing the paper on a main set of figures and analysis and moving several elements to the Supplement, as well as separating out main figures vs extended data vs supplemental analysis. We plan to further update in Nature style in future revisions. We have also highlighted several track changes in blue spotlighting important additions/changes to aid in review. The blue text doesn't capture all changes, since when removing or moving things to different sections this is not captured in blue.

In particular, you will see that reviewers raise comments in two main areas: framing and external validity. Some referees also suggest a new reorganization of the paper, which we agree would help clarify and streamline the narrative. We believe that these are important concerns, and while we are willing to overrule reviewers' concerns about novelty in relation to the NHB paper about Botswana given how this is an evidence-based contribution with a large sample, we can only consider a revision that clearly:

-Addresses the external validity issues pointed out by R1, R2 and to a lesser extent R4.
-Clarifies the methodological and design questions made by all reviewers, especially R3 and R4.
-Considers the streamlining and reorganization along the lines that R3 is suggesting to keep the Main text as focused as possible with the extra details in SI files.
-Engages with a theory of change and potential generalizability issues (R4).
-Addresses the remaining reviewers' comments.

We have made a substantial revision and thank the editor and reviewers for their constructive comments which have helped us refine the paper. We include a few

summary updates below and also outline a point-by-point response to each reviewer in depth below.

Some summary updates along the areas the editor highlighted:

(1) We have removed the external validity extrapolation section and made clear our advance with the multi-country RCTs is to rigorously estimate the distribution of effects across settings not to extrapolate or align effects, and include several clarifying caveats;

(2) We elaborate on several methodology and design questions throughout, such as (a) updating primary results from SD units to raw operations/proficiency gains in the main tables (b) reporting our main results by country, emphasizing and investigating heterogeneity across countries in more depth (c) and adding several data points and insights on counterfactual conditions to better contextualize and interpret results, among other updates outlined below;

(3) We have restructured and streamlined the paper substantially, removing additional analysis, such as the Karnataka quasi-experimental section and moving several analyses to the supplement to focus the paper on the main results, such as the typhoon analysis and results on several additional outcomes, such as non-cognitive skills;

(4) We have integrated into the paper an updated conceptual framework in line with the referee suggestion, including in a new section – "key components" of the intervention – and referenced throughout;

(5) We have further revised the manuscript according to all reviewer comments and elaborated on these responses in detail below.

We hope these updates effectively reflect the editor and reviewers' comments and appreciate the constructive comments which have helped us refine the paper.

Should further work allow you to address these criticisms, we would be happy to consider a revised manuscript (unless something similar has been accepted at Nature or appeared elsewhere in the meantime). We hope to receive your revised paper within four to six months. If you cannot complete the required revisions within this time frame, please let us know when you would anticipate being able to submit a revised manuscript.

Any revised manuscript should conform to our format instructions and publication policies (see here). We also strongly suggest that your revised manuscript has tracked changes, which is increasingly requested by referees to aid in their re-review.

We have made all the suggested updates and thank the editor and reviewers for their comments.

**Referee #1 (Remarks to the Author):**

It has been a true pleasure to review the Manuscript: Building Resilient Education Systems: Experimental Evidence Across Five Countries. I thoroughly enjoyed reading the article and would like to congratulate the authors on such an ambitious undertaking across five diverse contexts. I hope you and the authors find my enclosed comments useful. Please also accept my sincere apologies for the delayed submission, which was due to unforeseen personal commitments this month.

Comments are provided by Salman Asim:
Senior Economist, Education Global Practice, World Bank

Thank you for the constructive review and highlighting several features of the study, including the importance of multi-country nature of the evidence.

Summary

This is a technically strong and ambitious paper, coordinating five RCTs across countries during COVID with clean randomization, low attrition, and credible ITT estimates. The main results show that phone tutoring can offset steep learning losses, but the large effect sizes are better understood as averting learning losses rather than transformative gains. Claims of broad generalizability are overstated: the typhoon evidence and wide variation in impacts across countries (India (0.22 SD), Nepal (0.32 SD), and Uganda (0.89 SD) underscore that effects are highly contingent on crisis conditions and local context. The intervention is promising as an emergency response option, but it should not be framed as a universal blueprint for resilient education systems.

Overall, this is a highly valuable contribution, and I believe with more modest and precise framing, it will make an even stronger impact in the policy and research community.

Thank you for these comments. We have updated the framing in light of your suggestions, making clearer the application for education in emergencies. We also make clearer that while effect signs are consistently positive across contexts for phone call tutoring, effect magnitudes are indeed not uniform and vary across countries. We now elaborate on heterogeneity in magnitudes across settings with more emphasis. We also emphasize other resilience responses.

Detailed Comments

Angrist et al. study claims phone-based tutoring is a low-cost, scalable, and resilient model that sustained learning gains across diverse emergencies, with effect sizes (0.3–0.35 standard deviations) far larger than typical education interventions. The key highlights of the paper are: (1) the authors claim that findings are generalizable across contexts and modality of delivery (NGO vs Government teachers); (2) it is a cost-effective intervention at an average cost of about $11/child; (3) the gains held even during a major post COVID emergency (i.e. typhoon in Philippines).

Internal and External Validity of the Claims/Treatment Fidelity

The paper is exceptionally strong in setting up the RCTs. Treatment assignment was clearly randomized across multiple countries, with transparent documentation of the unit of randomization (household, grade, or school) and appropriate clustering of standard errors. The ITT estimates are well justified and consistently applied, and attrition was low (about 20%) with no evidence of differential attrition across arms. Causal claims within the sampled, phone-reachable populations are therefore highly credible. Internal validity is further reinforced by high engagement rates (70–80%) that limit concerns about selective compliance, by the replication of impacts across five countries, and by the embedded randomization of NGO versus government implementers in Nepal and the Philippines, which allows for clean causal inference on delivery models.

Thank you for highlighting these features of the paper which support strong internal validity.

My reading of the paper is less sympathetic when it comes to the external validity claims. What the study does convincingly show is that interactive phone tutoring worked for the sampled populations across multiple countries and delivery channels in the time of COVID, or more generally when schools were closed. Where I think the paper overreaches is in presenting this as a universally generalizable resilient systems solution.

Thank you for this point. We now caveat external validity claims in the updated paper. We make clearer that we do not claim universal generalizability. Rather, our study makes a contribution to *understanding several elements* of external validity, not achieving perfect generalizability. By conducting RCTs across contexts of a shared intervention, we provide new evidence on the degree of consistency across contexts.

The main issue is that any intervention capable of sustaining steady-state school learning during closures could have generated large relative effects. There is little in the results that suggested phone tutoring per se, rather than the mere continuation of

structured learning that drove the outcomes. Indeed, the reported 0.3–0.35 SD effect sizes line up with what we already know about pandemic learning losses. In my own longitudinal work in Malawi (Asim et al. 2024), students had lost about 0.5 SD by the time schools reopened after seven months, and these losses compounded to nearly 0.8 SD within the following half year as the gradient of learning slowed. By comparison, the Angrist et al. results suggest that tutoring helped children recover roughly half of what they would have learned in steady-state schooling — a valuable result, but not evidence of a transformative intervention.

Framed this way, the program is promising as an emergency response tool among others, but not yet proven as a generalizable model for all emergency situations. Unless the authors can show that the effects exceed the magnitude of lost learning in these countries, or that phone tutoring clearly outperforms credible alternatives such as TaRL-style community camps, the claim that this is the most effective or scalable solution feels overstated. A more balanced framing would recognize it as one plausible approach to mitigating learning collapse in crises rather than a universal blueprint for a resilient education system. It would also help if the authors benchmarked their results explicitly against pre-COVID annual learning gains in these contexts and situated their findings alongside other crisis-response interventions. Finally, I would encourage them to discuss more openly how the choice to standardize scores at the control endline shapes interpretation. In a setting where control groups are collapsing, these scaling choices are not trivial, and robust checks against baseline or pooled SDs would give readers more confidence in how to interpret the magnitude of the results.

We clarify the contribution and that the results indicate the approach tested is a a plausible one. We now further emphasize several other approaches as well. We note that many interventions do not work well at improving learning in emergency situations. So it is crucial to identify ones that do, especially across diverse settings and when delivered by government teachers.

We also note that in settings such as Uganda, effects both recovered learning losses and exceeded status quo learning trajectories, as shown in Figure E2. We also include an additional figure (Figure E3) now which more clearly shows learning trajectories across contexts. In Uganda learning stagnated and declined somewhat, while in other contexts learning in the status quo was positive but slow. We find the intervention stems some learning loss in Uganda, as well as achieves substantial gains beyond recovering learning losses. In other contexts, the intervention accelerates learning beyond status quo learning trajectories. This country-by-country analysis enables us to assess the effectiveness of the intervention in both recovering learning loss as well as accelerating learning, depending on the different status quo learning trajectories.

Thank you also for pointing out the choice of standardization. For this reason, we report non-standardized outcomes in the major tables now (operations gained and the proportion of students reaching the highest proficiency level), which show robust learning gains. We only include standardized outcomes in the Extended Data and Supplement now.

Originality/Significance of Contributions

The real strength of the paper lies in its scale and execution — coordinating five RCTs across countries and delivery channels under COVID is impressive and goes further than most education trials I have reviewed. The authors should feel accomplished in having delivered a well-executed set of trials, but they need not exaggerate the claims. In my view, the underlying idea is not novel: the model builds on the well-established principle of targeted instruction popularized by TaRL, though the authors adapted it to a phone-based, one-on-one format during school closures. The real significance is in showing that such a model can be implemented at scale (though it is important to note that the India Karnataka rollout is only quasi-experimental), at relatively low cost, and in COVID closure conditions with reasonably consistent results. Framed this way, the contribution is valuable, but more modest than the current narrative suggests.

On significance, the interpretation is more fragile than the narrative suggests. The apparent consistency of results is in large part an artefact of the crisis context — the study was conducted in regions like South Asia and Sub-Saharan Africa where school closures were unusually long and learning losses particularly steep (Patrinos et al.). In such settings, any intervention that sustained even modest levels of learning would show large relative effects. Moreover, the cross-country estimates in the paper are far from uniform: Uganda reports an effect close to 0.9 SD, the Philippines about 0.45 SD, while India, Kenya, and Nepal are closer to 0.3 SD. This spread in magnitudes raises important questions about why an intervention framed as highly effective yielded such different impacts across countries. It suggests the results are less universally stable than implied and remain heavily contingent on local conditions and the depth of learning collapse.

Thank you for these comments. We have updated the framing in light of your suggestions, making clearer the application for education in emergencies. We also further explore and emphasize heterogeneity as suggested. We also make clearer that we do not claim universal generalizability. Our purpose with running multi-site RCTs is not to extrapolate effects or to aim for effects to be the same, but rather to estimate the distribution of effects across contexts, furthering *understanding* of effectiveness across contexts. By conducting RCTs across multiple contexts of a shared intervention, we provide new and unique evidence across diverse settings and delivery models.

Interpretation of Results/Robustness

In Section VI.E the authors present an external validity analysis by testing whether conditional treatment effect estimates from one country can predict impacts on another. Their own results show the opposite of what their narrative suggests: extrapolations fail badly. For example, extrapolating from Uganda's 0.89 SD effect produces implausibly high predicted impacts in other countries, while India's more modest 0.22 SD effect is mis-predicted by large margins — in some cases even yielding negative expected effects, such as India to Nepal (-0.07 SD). The clear finding is that baseline covariates cannot explain treatment heterogeneity, and that treatment effects do not transport cleanly across contexts.

This validity exercise needs to be interpreted for what it shows: the intervention's impacts are highly contingent on local contexts and cannot be assumed to generalize. If anything, the stress test undercuts the broad claims of consistency and resilience — it highlights the fragility of the results across settings. The true policy-relevant takeaway is sobering: a policymaker cannot be sure what impact to expect in their own context. And if that is the case, how can the intervention be generalized as a blueprint for education emergencies? The only claim that seems defensible is narrower: in contexts where learning collapses as it did under COVID, phone tutoring can help recover a portion of the lost ground.

We agree that this section did not yield enough added insight and have now removed this section on the editor and reviewers' suggestions. Our goal was to showcase the value of conducting an RCT in multiple contexts, rather than only extrapolating based on effects from other settings. But we agree that the value of a multi-country study is evident already, so we have removed this section to focus on the main results as suggested by the editor and reviewers.

**Referee #2 (Remarks to the Author):**

This paper evaluates a remote tutoring program to maintain student learning during breaks in schooling. Breaks may be due to emergencies like the COVID-19 pandemic, natural hazards like a typhoon in the Philippines, or even school vacations. The authors test two delivery mechanisms in a multisite RCT in 5 countries: SMS messages and phone calls. They find that the program, especially the phone calls, leads to large increases in learning measured by correct answers to a series of progressive questions. Several features of the implemetation and evaluation design allow this paper to make important contributions to the education literature and also to a broader literature on the

promises and limitations of RCTs as tools for learning about policies.

First, and most obviously, the impressive scale of data collection across five countries allows us to learn what works in different contexts.

At the same time, the degree of control and attention to detail across the five sites seems noteworthy for a multisite RCT. This control reduces undesirable variation in program implementation and gives us some insight into where differences are coming from. My favourite example of this is captured in Figure A4, which shows the percentage of instruction that was targetted in the different sites. Having a standard shared protocol that defines and measures targetting in the same way across sites, and collecting this type of data on intermediate stages of implementation, allows the authors to establish that there is a correlation between the extent of targetting and the size of the treatment effect. This increases the plausibility that targetting is an important mechanism as they theorise.

Several other design details deserve merit: randomising whether the implenters are from NGOs or government in Nepal and the Philippines addresses questions about whether RCT effect sizes are only due to motivated implementers; randomising off a list of eligible teachers in Nepal gives us some insights into the question of whether program impacts go through teacher selection or the program itself; multiple checks and repeated measurements increase confidence in the assessment tools.

Thank you for these constructive comments and for highlighting several of the key features of the study. We went to great lengths to run coordinated, high-quality RCTs across settings, with multiple different types of randomizations to elicit insights on mechanisms and scaling dynamics, and to collect high-quality robust data. We are happy to hear our effort and study resonated with you.

Major comment 1:
The biggest weakness of the paper is the external validity analysis presented in section VI.E. The authors present it as an analysis that highlights the benefits of a multisite RCT over the state of the art. However, it's so perfunctorily done that I don't find it very useful. I don't have the impression that many people following the state of the art would expect covariates that do not affect treatment effects in one setting to be useful predictors of treatment effects in another setting. So the finding that baseline learning, gender and student grade—which are not associated with any treatment heterogeneity (Table A6)—are not useful covariates for predicting effects in other sites is not very insightful.

In fact, going back to the Hotz, Imbens and Mortimer (2005) paper cited, the original authors raise at least two reasons why sites might have different treatment effects. "First, the distribution of characteristics in the two populations may differ. If [a characteristic] is associated with the outcomes of interest and in particular (emphasis mine) with program efficacy, average program effects may be found to differ for the two programs....The second reason that treatment effects may differ across programs is that the programs, even if nominally the same, are heterogeneous in their components...[or]...may differ qualitatively."

The second reason, in light of the evidence the ConnectEd authors present on targetting, seems to be the most relevant. It seems that engaging with that aspect of the external validity literature is likely to yield more productive insights.

We agree and have now removed this section on the editor and reviewers' suggestions. We now also make clearer that we do not claim universal generalizability. Our purpose with running multi-site RCTs is to estimate the distribution of effects across contexts, furthering *understanding* of effectiveness across contexts, not to extrapolate effects or to aim for them to be the same. We elaborate on this in related responses to other reviewer comments. We now also further investigate heterogeneity as a dimension of interest, such as the degree of targeted instruction, as highlighted by the reviewer.

Major comment 2:
The statistical analysis is competently done. I could find most measures of uncertainty I searched for and p-values, confidence intervals and standard errors are appropriately presented. I would only request additional information on the multiple hypothesis corrections presented in Table A9. How many hypotheses are being corrected for? Is it 5 for the different countries; 2 for the different treatment arms or 5 x 2 for the interaction between them?

We have now clarified this in the paper. Since we have one outcome and two treatment arms, we consider two joint null hypotheses simultaneously.

It would also be helpful to see the Karnataka evaluation at scale results presented in similar standard deviation units as the main results. That would make comparisons easier.

Thank you for this comment. We have now removed the Karnataka section based on comments from the editors and reviewers.

Major comment 3:
The paper is very well written, but I still had some questions about implementation

details that I couldn't quickly answer. First, for each site, do we know whether children were in school or not? Figure A7 shows that some schools were partially closed at the national level, but what does that mean for the actual children and families who were enrolled in the program? It's likely that some of the enthusiasm for the program was driven by children, families, and teachers who had no alternatives. Essentially, I would like to see the data from question 10 of the baseline and endline questionnaires presented. It would be helpful to interpret results in light of how the tutoring was interacting with any, likely limited, schooling opportunities.

We have now included a new Table E8 explaining counterfactual conditions more clearly. This includes the extent of school disruption. We also refer to these counterfactual conditions in our mechanisms section. Indeed, it appears that the strongest learning gains occurred in countries with the most severe counterfactual conditions as your comment suggests. Thank you for pushing this analysis further.

We code a school disruption variable as an index (0 = schools open during the study; 1 = schools partially closed during the study; 2 = schools largely closed during the study). To code this variable we combine information from question 10 with the timing of the endline and the timing of official school closure policy. We also include additional data on counterfactual conditions where available, such as the extent of other educational instruction on offer and accessed by households.

Second, what explains the differences in implementation lengths (Table A8)? Were there differences in the 'doses' of the program?

We have clarified the detail on implementation lengths. Nepal had two different cycles – an NGO and a government cycle -- and we report results per cycle so implementation lengths were similar at around 8 weeks. Thank you for catching this. The intervention length was standardized to 8 weeks across all intervention settings and delivery models. The only variance was Kenya which has a slightly longer implementation since it started shortly after a break, so the normal 8-week intervention cycle took place after an initial set of 4 pilot weeks and then the full implementation took place. We have included the pilot weeks in the total count for clarity and to be conservative since implementation took place, but the standard main dose of the intervention was 8 weeks.

Some of this might be addressed by slighly re-writing the country context subsections in III.D so that each country sub-section explicitly follows the exact same structure. This would allow a reader to more easily grasp the peculiarities of each implementation.

We have moved the trial description table into the main tables to help provide more clarity among other clarifications made throughout.

**Referee #3 (Remarks to the Author):**

Review of Nature MS 2025-07-17892

OVERVIEW

This is a well-written paper on an ambitious set of experiments that aimed to address the educational emergency caused by school closures during the COVID-19 pandemic. The authors document the effect of phone-based tutoring and, with less emphasis, SMS-based educational content delivery on learning outcomes of students in primary grades in five distinct settings (Kenya, India, Nepal, Philippines and Uganda), based on a model that had previously been developed and evaluated in Botswana (Angrist et al, Nature Human Behaviour 2022). Using short phone-based assessments, they document impressive effect sizes on student achievement of ~0.3-0.4 standard deviations (s.d.) after about 8 weekly lessons of 20-minutes each. These aggregate estimates hide substantial heterogeneity ranging from between 0.085 s.d. in Kenya to 0.89 s.d. in Uganda for the same intervention. The authors make the case that the program, and the evidence, are both relevant beyond the COVID-19 pandemic, with applicability also to other emergencies that occur periodically in many parts of the world — they provide suggestive evidence from Philippines and India to this effect. In specific settings, the authors also test some sub-randomizations (such as NGO versus government delivery) but these are secondary to the main focus of the trials.

SUMMARY OF COMMENTS

There is a lot to like in this paper. I especially appreciated both the immense efforts of the authors in setting up the core intervention in multiple countries, with multiple partners in the challenging setting of the pandemic, as well as the commitment and flexibility to subject each iteration of the program to a randomized control trial. The core result — that the composite phone-based tutoring intervention was effective in raising achievement — is clear and unlikely to be sensitive. Changes I recommend below are mostly expositional and not primarily centered on the scientific validity of the authors' claims (although they matter for clarity and transparency).

To my mind, it is clear that this paper deserves to be published in a reputed general interest journal. The specific need that the intervention addresses (remote instruction for foundational learning in low/middle-income settings) is widespread and, as the authors demonstrate, the specific intervention can also be adapted across settings. These authors were at the forefront of the literature on remote instruction during COVID-19

and, although there were several contemporaneous experiments (including e.g. a 7-country study in Latin America, plus others in Spain, Italy, India etc.) with mostly positive effects, I think they can legitimately claim to be the leaders in that space. There is some policy momentum now behind exploring remote tutoring outside of COVID-19 settings, including the scale-up discussed in this paper, and I think at least some of this reflects the dissemination of the research discussed in this paper. So, it is clear that this stream of work has been influential.

Thank you very much for these comments. We appreciate the acknowledgement of our scientific efforts and also you highlighting the policy influence of the work. We went to great lengths to run coordinated, high-quality RCTs across settings, with multiple different types of randomizations to elicit insights on mechanisms and scaling dynamics, and to collect high-quality robust data. We are happy to hear our effort and study resonated with you and that the wider impacts on the literature have been felt.

Whether the specific scientific innovation is adequate for Nature, and whether the paper represents a sufficient advance over the knowledge in the first experiment in Nature Human Behaviour (Angrist et al, 2022) and the many other contemporaneous studies, is a decision for the Editor and I will not have comments in that direction. My comments below focus on expositional changes that, in my view, are essential for the paper to send out a clear actionable message for a broad readership. That being said, the Editor would also be in a much better position to judge the requirements for the Nature readership than a typical Economics paper.

MAIN COMMENTS

#1 Reduce the number of reported analyses
The paper would be much better served if it narrowed down the scope. While I appreciate the effort the authors took across multiple distinct empirical analyses, they are not equally important and much of the analysis distracts from their main points.

To my mind, the paper has three main results: (i) phone-based tutoring support was effective in raising student achievement in very diverse settings, (ii) SMS-only interventions were typically not effective and (iii) there is huge heterogeneity in treatment effect sizes across contexts and experiments, which is not explained by measured sources of variation. Everything else is a distraction. The paper would be well-served in only highlighting these.

In particular, I recommend that various sub-treatments (govt vs. NGO), the Philippines typhoon subsection, the analysis of the India scale-up (which is non-experimental, and

is half-hearted by the standards of high-quality non-experimental causal evaluations) etc. should all be removed from this draft. So should the validation of the phone survey in Kenya. These are not the features that lead this paper to be of general interest, nor are they necessarily the most robust parts of the analysis.

Thank you for these constructive suggestions. We have substantially focused the paper as suggested. We have emphasized the three points listed as primary and also elevated the discussion of heterogeneity more. We have also removed some sections entirely (including the India quasi-experiment) as well as moved much of the secondary analysis mentioned to the Supplement. We have kept the analysis of the effectiveness of NGO vs government delivery, since this is a major benefit of having this as part of the experimental design and since this is a big question in the science of scaling literature.

#2 Explain the core experiment design and results more clearly
Getting rid of secondary analyses, and associated exhibits, also frees up space for the authors to expand their exhibits to provide useful insight about the actual core experiments.

There are two issues here that I think are first-order importance:

a) Details of experiment design:

Move Table A.8 to the main body of the paper. This is not appendix material.
Please also indicate the number of treated and control individuals in each experiment separately, as well as details of attrition (for each nd). I recommend a full CONSORT style diagram for each of the 5 experiments as supplementary material.
Please also discuss the representativeness (or not) of the population compared to the broader school-going population in each of these countries. It is not clear from the draft how the sampling frame was identified, how households were approached and whether there were extra selection criteria for the study before the randomization. I suspect there were some such criteria since I note that the response rates for the surveys are considerably in excess of those found by other research teams from lists of phone numbers in developing countries during COVID-19.
I note that in the Declaration accompanying the submission, the Corresponding Author confirms that the paper reports “the exact sample size (n) for each experimental group/condition, given as a discrete number and unit of measurement ” (emphasis mine). I apologize if I have missed it but I did not find this information. Specifically, I cannot on the basis of Section III. C, Section III.D or Appendix Table A.8 answer just how many students were randomized to be treated in each of the individual experiments? In the countries which featured further sub-randomization (e.g. NGO vs. government implementation), I could not tell whether these were randomized with equal

probability or not. As a summary measure, I cannot tell here what the total number of treated individuals across the 5 studies was. These are crucial details for interpreting this experiment, and also for comparing with the many other studies featuring remote instruction. The authors should include this in the main body of the paper.
Please note that, while I classify these comments as expository, issues of sampling, representativeness and attrition for each experiment individually go to the heart of the scientific validity of the claims (and the implications for policy). It is essential that this is fully specified in at least the supplementary material for readers.

Thank you for these comments. We have now added more detail and description as suggested, including moving the table describing each trial to the main paper. We also include a CONSORT diagram adding more information by treatment arm in the Supplement. We also include data from similar national assessments measuring similar proficiencies to the ones conducted in our study in a new table (Table E8). In general, our study samples were largely representative of national learning patterns. For example, in Uganda, 9 percent could reach the top division operation level relative to 15 percent in a national survey in Uganda; in Nepal 22 percent of our sample could reach the top level relative to 23 percent in a national survey. Across all countries where we have data, our sample was within just 1-6 percentage points of the national average.

b) Transparency in presentation of results across experiments

The greatest strength of this study is the running of 5 distinct experiments across settings. Given this, I do not understand the choice to present results in the aggregated (pooled) sample as the main specification.
As I see it, your main result is Table 4 and that should be your key exhibit.
The second table that should be emphasized as the “main” result is Table 5 but with a separate panel for each experiment.
Please do not pool across experiments in this manner, especially given different settings, sampling protocols, implementation instructions and randomization procedures — doing this properly would require very different Bayesian approaches (as Meager (2019, AEJ:Applied) demonstrated for experiments on microfinance). In that table, please also have the baseline value and the control mean for each individual country sample in each panel.
This is very important since we do not have any indication in the manuscript about differences in baseline achievement across your samples (i.e. differences between e.g. Philippines and Kenya, not the treated and control groups within each country). Given the coarse categorization that is possible with these short ASER type assessments, it is possible that much of the difference across experiments comes from a “standard deviation” being very different across each of the individual samples. It would be far

more transparent to present actual proficiency gains in the 5 samples separately. Formatted suitably, this could be the key exhibit of the paper, easily contained in a single (full-page) exhibit.
This also would be a more principled way of dealing with the coarseness of the ordinal ASER-type measures. For an ordinal variable that only takes 4-5 categorical values, the standard deviation metric seems particularly difficult to interpret. This is even more so when comparing across vastly different contexts. I understand that you have to report the z-score results somewhere but it would be far more informative to see the competence-level results than the aggregated scores.

Thank you for this constructive and clear comment. We have now updated the main exhibit accordingly and agree the main results are much clearer. Table 1 now presents results by country in raw units – both the number of operations gained (non-standardized) and the percent of students reaching the top respective level. This is broken down for each country. This should address several of the concerns stated, such as pooling concerns discussed by Meager (2019) and Angrist and Meager (2023). Table 2 also presents operations gained (non-standardized) by country for the trials that include NGO and government delivery.

I would recommend dropping the section of scale up in India entirely. It detracts from the core of the paper and does not speak directly to the rest of the paper — I suspect the authors agree, which is why the entire section only leads to Appendix exhibits and not a single exhibit in the main body of the paper.
Incidentally, the issues of ordinality are especially a concern with the difference-in-difference approach in the analysis of the scale-up. Analysis of changes only makes sense with an implicit assumption of cardinality: a change from 1 to 2 has to equal the change from 3 to 4. This would be okay if this scale was approximately equal-interval but it is not. Ordinality is, of course, a broader problem when it comes to test scores (see, e.g., Bond and Lang 2013) but it is especially severe for short assessments that only permit a relatively coarse categorization.
It is possible, given the data, to write a much more compelling non-experimental evaluation of the scale-up. However, the space it would take to do this well is not feasible in the current paper. Nor do I think it adds to the clarity of the paper to have that also tacked on without doing full justice.
For transparency, I would also recommend being upfront about whether any of the analyses were pre-registered or not. Please note that I do not think there is anything fishy here – the analyses and outcomes are pretty standard but I suspect it is worth clarifying this in the draft as such pre-registration has moved to being increasingly common in the field. I don't think anybody would object to the lack of a full PAP while trying to coordinate a multi-country effort in the midst of COVID (when I'm sure you had

very little ex ante knowledge of how well implementation would occur). I suggest this only for transparency.

We have removed the India quasi-experimental results as suggested. In terms of the pre-registration, we include a link to the registration. On the PAP, as the reviewer notes, this was difficult during covid, but as noted our study has a straightforward set of outcomes and treatments and results are highly robust. We now include mention of this in the paper.

#3 [OPTIONAL] Consider a crisp, short paper

This is purely a suggestion, offered with free disposal. In my view, the signal achievement of this project is to have conducted 5 experiments with multiple partners across countries in the midst of a severe education crisis. Communicating the very positive results from this endeavour should, in my view, be the single purpose of this paper.

For this single purpose, it is adequate to just report on the design of the core experiments and the main results on learning outcomes. Effectively, that means three tables: (i) to show sample characteristics and balance; (ii) to show effects on the aggregated standard deviation metric (currently Table 4); and (iii) to show effects on individual competences (Table 5, but by country). Everything else should go to supplementary materials.

If Nature allows, I would recommend a well-fleshed out Discussion section where you can discuss relevance to (i) other emergencies, (ii) post-COVID FLN, (iii) scale-ups, (iv) mechanisms etc. But these are NOT the main value-added of this paper or this effort.

Given that the paper is in consideration for a broad readership, much beyond just economists, I think a crisp 5000 word paper with 3-4 exhibits would be far more influential in the world than a bloated paper with too many secondary analyses written for an Economics audience.

Thank you for these constructive and clear comments. We have now updated the main exhibits and moved more material to the supplement as suggested. We agree the streamlined paper is much clearer and more focused. We have kept the overall format similar for now, while consolidating and focusing the paper, for ease of reviewing, and plan to update to full Nature style in subsequent revisions.

Referee #4 (Remarks to the Author):

Comments "Building Resilient Education Systems: Experimental Evidence Across Five Countries"
The paper presents causal evidence from an educational intervention that utilized SMS messages and calls with a teacher or tutor. The intervention took place in five different countries during disruptions to the school year, primarily due to school closures during COVID-19. It focused on mathematics, typically targeting grades 3-5, and employed regular teachers and tutors. The intervention was implemented in some locations through an NGO and in others via public schools. It employed random assignment, allowing for the identification of causal effects. The paper shows evidence of positive effects on a five-question math test (simple operations). Additionally, the paper presents tests for external validity using country-based covariates, explores the modality of delivery (public versus NGO), assesses individual countries, among other analyses. There is a lot to like about the paper. It is very impressive how the authors have polled together all these individual experiments.

Thank you for your comments and for highlighting several features of the study.

Comments
• Please clearly explain the theory of change. My recommendation is to use the framework of Nickow, Oreopoulos, and Quan (on tutoring) and adapt it to the different design characteristics of this program.

Thank you for this suggestion. We have now made reference and used several elements of the framework in Nickow, Oreopoulos, and Quan. We include a section outlining several key components of tutoring interventions adapted from the suggested framework, covering curriculum characteristics, tutor typology, delivery mode, and dosage, and also include an adapted theory of change in the Supplement.

• An important discussion point is the description of the counterfactual: what did the control group receive? Did they have instruction? Who provided that instruction—was it the same teacher as in the intervention? Did they meet with a professor regularly? Etc. The description of what happens with the control group is critical for understanding the effect size.

We now include more information on the counterfactual along a few margins. First, we include a new table (E8) which describes the counterfactual condition in each country in more depth. In addition, in the context section we describe what else was on offer by the government. We further show in Figure A5 the timing of the intervention as it relates

to school closure vs opening. Finally, we include control means in each table to indicate status quo rates. In general, we find that some instruction was being provided in the counterfactual condition but this was usually minimal. For example, we find that in all cases less than a third of students are practicing math problems at home in the counterfactual conditions. We also find that while many governments offered instruction through radio or television this type of status quo instruction rarely seemed to translate into sizable educational engagement (e.g. only 2-5% of students tuned into radio instruction in Nepal and the Philippines where we have good data on this measure). We also now include a row in Table 5 to indicate where instructors for the intervention were the same or not as students' regular school teacher.

• I think a better description of the treatment and its interaction with "regular" schooling is important. Similar to the control, it is important to understand what the treatment group received from the school. Did they meet regularly with the "regular" teacher? Was it the same person delivering the tutoring? Did they only meet once per week for 20 minutes?

We now include more information on status quo education offers as described above. In general, it appears the counterfactual condition was a more passive education offer rather than active educational engagement and had low engagement. This means the treatment often ensures some continuity in the degree of educational instruction provided even amidst disruptions, which was the intention of this type of education in emergency tutoring approach.

• The effect sizes are quite striking, considering the low dosages, the fact that it was during COVID, and the fact that it was implemented without major training for teachers/tutors. For example, an effect size of 0.891 SD (!) in Uganda is extremely high. One explanation for the effect sizes lies in the metric of the outcome. The outcome is a five-question test in a very narrow domain.

We now report our main effects by raw units and by proficiency and highlight this as the main set of results, and to avoid potential concerns in standardization. The standardized effects are now only included in the Extended Data and Supplement – we include them since they remain common in the literature, but we agree that proficiency-based reporting is much clearer and more reliable and focus on this in our main analysis.

o Please present the distribution of the test. You standardized the test—please show the graph of the standardized tests.

We now report main effects in raw units and by proficiency, with standard deviations only included in the Supplement. Figure A3 also shows the distribution of the test at baseline and endline and by treatment group.

o Please present the correlation of these five questions with a more meaningful test, either a national assessment or an international test.

In low-resource countries, international and national tests are often not the relevant benchmark since they are many levels above actual learning levels of children and often experience flooring effects. For example, children who can't add are asked to solve systems of equations, resulting in flooring effects on international tests which struggle to differentiate more basic skills. This is a well-known issue, as shown in recent PISA for development results, and is a point emphasized in high-profile studies by Banerjee et al. (2007), Muralidharan et al. (2019), and highlighted in the World Bank World Development Report 2018. In order to avoid flooring effects, tests which capture a set of simple basic proficiencies are essential and *are often the most meaningful test in these contexts*. The proficiencies in the tests we use are aligned with UNESCO's Global Proficiency Framework used to monitor the Sustainable Development Goals. Other studies have used similar tests and correlated them with other measures, such as comparing the ASER and EGRA test and found high correlations. Moreover, by reporting main effects on clear proficiencies now rather than standardized scores – such as the ability to add or divide – we emphasize a clear set of meaningful measures.

• Relatedly, how do we know that the teachers/tutors did not teach to the test? By this, I mean that rote memorization can produce this type of effect. Can you provide evidence against this?

We included specific validity tests to check for this. For example, as shown in Table A2, we randomized different numerals of similar proficiencies to ensure we capture actual proficiencies rather than recall on familiar questions. We further include in Table E6 several proficiencies that were never directly taught but would be expected to be learnt later on (e.g., fractions if students were only taught up to division) or translating skills into real-world scenarios (e.g., word problems). We see gains in these proficiencies as well showcasing a broader set of learning gains. To specifically address the point on memorization, which we take seriously: (i) if effects were due to rote memorization, randomizing numerals would reduce the effect — but we find it doesn't; (ii) moreover, if effects were driven by rote memorization, untaught spillover competencies (fractions) wouldn't show effects — we find they do, in addition to other spillover competencies.

• On the intervention:

o Was the intervention conducted via the parents' phones? If so, how many phones per family? Is it credible that the parents would lend the phone to students for the duration of the intervention?

Yes, the intervention was often mediated through a parent’s phone. Nearly all families have a phone and we find high response rates week-on-week as shown in Figure E1. The intervention was deliberately designed to be short in order to facilitate engagement, which is one reason we see such high responsiveness. Moreover, parents in the settings we work with appear to value the intervention and prioritize it as shown in Table A3.

o At what time of the day were the phone calls made? The timing of the phone calls has been shown to have a large impact on these types of interventions.

The timing of calls varied for each household by design. Each tutor was responsible for a set of households and would prioritize their availability, identifying and coordinating with families to call when worked best for them. This model was adopted deliberately to maximize reach and dosage. The value of this approached is evidenced by the high engagement with the intervention (as shown in Figure E1). This contrasts with the low engagement with counterfactual options (as shown in Table E8).

o Exactly how was the “teaching at the right level” implemented? The domain of the intervention—math, specifically the four operations—does not align well with TaRL. If a student did not grasp summation, would the tutor continue focusing solely on summation? Did the tutor vary the level of summation (e.g., one-digit versus two-digit summation)? How did tutors record the progress of students?

The teaching at the right level approach in this study was modelled after prior studies (e.g., Banerjee et al. 2007; Banerjee at al. 2017) which focused on these same core competencies in mathematics and a similar approach for assessment and content progression.

Similar to other studies, at baseline, tutors conducted a baseline test that assessed all operational competencies: addition, subtraction, multiplication, and division (all 2-digits). Tutoring started at the next level after the highest level the child attained. For example, if a child could add but not subtract, the tutor would teach subtraction; if the child could not add, the tutor would start teaching addition. Each week, the tutor would end the session with a “checkpoint” question that was used to conduct high-frequency checks on the child’s understanding. If the child who couldn’t do subtraction now could, the tutor would move on to the next competency the following week: multiplication. Progress was recorded on paper sheets each week and entered into a digital data system. This is how

we are able to calculate rates of "targeted instruction" in Figure E4. Well-targeted instruction is coded based on two conditions: (a) if tutors progress when a child masters the prior concept and (b) if tutors do not progress when a child does not master the prior concept.

o The students were in grades 3 to 5: were the content and level of tutoring the same for all?

Since in the settings the study took place, nearly all children are behind grade-level expectations and have not mastered the basic operations, the content was similar across grades. This is a similar approach to many interventions focused on foundational skills in LMICs, including several of the leading teaching at the right level studies. This is consistent with a wider set of findings on the global "learning crisis" where students are well behind grade level expectations and cannot master basic foundational skills expected to be learned in grade 2 and 3, even well into later primary schools grades. This has led to a call to focus on foundational skills across all primary school grades.

o The paper claims that one of the "mechanisms" is "Platform: reach at the right level." For me, it is unclear what this means. The implementation uses phones, and the actual phone calls are flexible. However, this seems more about implementation than a mechanism. My suggestion is to reframe that discussion.

We have now reframed this discussion as suggested. The platform refers to the fact that many households have access to a mobile phone, but do not have access to the internet, for example. Therefore the phone is a platform that households can be reached on at scale and cheaply. We have now updated the conceptual framework as suggested and also capture more detail on the delivery mode.

o Was there training provided for teachers/tutors?

Yes, there was practical training provided for all interventions. We now describe this in more depth.

• I would like to see a discussion about the initial level of students and whether the intervention was able to restore students to pre-COVID levels. In other words, was the intervention able to "increase" learning or just to recover lost learning?

Control means are included in the key tables now in clearer units. We also include detail on learning loss vs learning acceleration. In some cases, such as Uganda, as shown in Figure E2, we see a combination of learning recovery and acceleration. We now also

include a new Figure E3 which shows results from countries where we had a baseline and endline with identical tests. This figure shows that in Uganda learning lost was recovered and learning was accelerated. In other countries, learning was accelerated relative to slow rates of learning progression in the status quo.

• I believe that the discussion of "Results from Another Education Emergency" is not particularly interesting. My suggestion is to move that to an appendix and mention it briefly in a few lines.

We now move this to the Supplement.

• On the teachers:
o What is the complementarity between tutors and teachers? I asked before, but let me reiterate: were the same teachers delivering the program as those who provide regular teaching?

In some countries, the teachers were the same as the regular teachers (e.g. Kenya) and in some they were different (e.g. Nepal). In some cases, the instruction was provided by a tutor (e.g. Philippines NGO treatment) not a teacher and in some cases a teacher (e.g. Philippines government arm). We now include more information on this in Table 5 as requested. It is difficult to examine the degree of variation these dimensions affect since they often do not vary enough or systematically enough across studies. This type of cross-country correlate comparison is inherently challenging. However, we do have experimental variation comparing tutors versus teachers embedded within two trials. We show that government teachers and NGO tutors seem to be similarly effective. While this dimension is often very important, in our study it does not seem to drive observed differences in effects. We explore other descriptive patterns which vary systematically across countries; for example, we see that rates of targeted instruction and the severity of the counterfactual condition appear to be plausible dimensions driving higher treatment effects.

o The table of teacher practices shows self-assessed metrics. For instance, 76.9% of teachers in the control claim that they "target to student level." We know that this is not the case. Precisely all the agenda of TaRL is based on this false assumption. Similarly, involving parents is costly, and we know that teachers would prefer not to engage parents. I don't have a clear direction with this comment, except to temper expectations regarding the results.

Thank you for this comment. We have now caveated results and agree absolute rates could be inflated due to social desirability bias and should not be interpreted as the

ground truth rate of targeted instruction. Of note, while absolute rates might well be inflated, treatment effects should remain unbiased since social desirability affects all groups.

Small comments:

• The 222 million children with disruptions: are they counted per year?

Yes, this is per year according to Education Cannot Wait.

• Page 18: “The follow-up rate was very high at around 80 percent of respondents at baseline.” That is quite a standard response rate; it is not actually “very high.” Moreover, an attrition rate of 20% is quite serious.

In the context of disruptions, this response rate has been considered high, but we have reframed this to be simply descriptive and to make clear response rates are unbiased across treatment arms.

• In the section “A Follow-On Evaluation at Scale,” you don’t need the first two equations ($\Delta Y0$ and $\Delta Y1$).

We have removed this and moved this discussion to the supplement and reduced emphasis on it.

Nature response to editor and reviews

____

Editor

Dear Dr Angrist,

Your manuscript, "Building Resilient Education Systems: Experimental Evidence Across Five Countries", has now been seen by our referees, and in the light of their advice I am delighted to say that we can in principle offer to publish it. First, however, we would like you to revise your paper to address the points made by the referees, and to make some editorial changes to your paper so that it is as brief as possible and complies with our Guide to Authors.

…

We will not send your revised paper for further review. If the revised paper is in our format (as detailed above), in accessible style and of appropriate length, we shall begin the acceptance process…

Thank you for the constructive editorial process. We are very pleased the updated manuscript has addressed all major editorial and reviewer comments. We are thrilled to see the paper published in Nature soon. We have applied some finishing touches as per some of the editorial and last light reviewer comments, described below, and have also updated the manuscript to fit Nature style as per the guidelines provided by the editor. We have updated the title, revised the reference style, moved sections II/III to the Supplement and section IV/V to the methods section and included a summary of these sections in the main text. We included disclosure statements, integrated and adjusted all footnotes in the main text and methods, and softened several claims based on appropriate statistical inference (for example, referring to SMS results as “mixed” and stating that both NGO and government delivery improved learning, rather than directly comparing them). We also moved the suggested prior Figure A4 to the Extended Data (now Figure E5). We produced Figure 2 to help visualize the key results. This figure presents no new information, but rather present a subset of results from former Table 1 (now Extended Data Table 1).

---

Referee #1 (Remarks to the Author):

I appreciate the substantial revisions made in response to the review. The updated manuscript now presents a considerably more nuanced discussion of external validity, heterogeneity across contexts, and the role of alternative resilience approaches.

I also welcome the decision to move standardized outcomes out of the main tables and to report non-standardized learning measures more prominently. Finally, I appreciate the removal of Section VI.E, which I found difficult to reconcile with the broader interpretation of the results.

While I continue to believe the magnitude of the effects should be interpreted in light of

unusually severe learning disruptions during COVID, the revisions have substantially improved the balance between the evidence presented and the claims made. Overall, I believe the manuscript has been materially strengthened!

Salman Asim
Senior Economist, Education Global Practice, World Bank

Thank you for the constructive comments. We are very pleased the updated manuscript has addressed your comments. Thank you for helping us strengthen to paper.

---

Referee #2 (Remarks to the Author):

Thank you for the thorough review. The paper is streamlined and a pleasure to read. The additions and re-organisation improve clarity of what I see to be the key ideas.

Thank you for your comments and support for the paper. We agree the updated manuscript is much improved and thank you for your helpful comments to improve the paper.

My remaining comments are minor, and with some of them, I realise I am veering into the role of an unasked for copy-editor, so please consider them as suggestions rather than points that must be addressed.

1. I like the changes to section III and the discussion added to VI.B. They both help to give a clearer idea on the mechanisms that lead to the effects, and suggest some explanations for the heterogeneity we observe. I wonder if the two sections could be better integrated with each other. For example, by merging them into a single section after the results. On the other hand, I did not find Figure A6 very helpful. The paper mostly discusses "Tutoring inputs" (in the two sections I referenced) and does not say much about "Mechanisms of impact". I found it distracting to see a list of "Mechanisms of impact" listed with little idea of how they translate to the context.

We agree with this – to simplify we have focused on the core mechanisms as highlighted.

2. I think Table A7 is reporting differences in means between the teachers randomised into the program and those who were not assigned to the program. If that is the case, I would prefer that is stated explicitly rather than saying "this table reports the balance..." which could mean different things in different settings.

We have now clarified this.

3. Footnote 4 seems unnecessary as a paragraph-length footnote in the introduction. The material discussed in it is repeated in Section IV at the bottom of page 17. It is better placed there.

We have now removed this since as you note since this information is repetitive and mentioned later and the Nature format doesn't allow footnotes.

---

Referee #3 (Remarks to the Author):

I have now re-read the revised manuscript, as well as the authors' response to reviewers and the supplementary data appendixes.

I congratulate the authors on a thorough revision, and even more on having pulled off this very ambitious project in the midst of a global pandemic.

I have no further comments to offer and, if the Editor and other referees permit, hope very much to see this appear prominently in Nature after any changes needed to conform to Nature formats/guidelines.

Thank you very much for the praise for the paper and the project. Your review is much appreciated and has strengthened the manuscript.

---

Referee #4 (Remarks to the Author):

I read the second revision of the article. I believe that the authors addressed all my comments in a satisfactory manner, especially the comments about the theory of change and the counterfactual. I believe that the paper is now greatly improved. The fact that the authors streamlined the results and focused on fewer treatments and experiments (leaving aside, for instance, the quasi-experiment in India) makes the paper more readable.

Thank you for the constructive comments which we agree have helped to streamline and focus the paper. We are happy the updated revision has addressed your comments and look forward to seeing the paper released.

---

Summary

This is a technically strong and ambitious paper, coordinating five RCTs across countries during COVID with clean randomization, low attrition, and credible ITT estimates. The main results show that phone tutoring can offset steep learning losses, but the large effect sizes are better understood as averting learning losses rather than transformative gains. Claims of broad generalizability are overstated: the typhoon evidence and wide variation in impacts across countries (India (0.22 SD), Nepal (0.32 SD), and Uganda (0.89 SD) underscore that effects are highly contingent on crisis conditions and local context. The intervention is promising as an emergency response option, but it should not be framed as a universal blueprint for resilient education systems.

Overall, this is a highly valuable contribution, and I believe with more modest and precise framing, it will make an even stronger impact in the policy and research community

Detailed Comments

Angrist et al. study claims phone-based tutoring is a low-cost, scalable, and resilient model that sustained learning gains across diverse emergencies, with effect sizes (0.3–0.35 standard deviations) far larger than typical education interventions. The key highlights of the paper are: (1) the authors claim that findings are generalizable across contexts and modality of delivery (NGO vs Government teachers); (2) it is a cost-effective intervention at an average cost of about $11/child; (3) the gains held even during a major post COVID emergency (i.e. typhoon in Philippines).

Internal and External Validity of the Claims/Treatment Fidelity

The paper is exceptionally strong in setting up the RCTs. Treatment assignment was clearly randomized across multiple countries, with transparent documentation of the unit of randomization (household, grade, or school) and appropriate clustering of standard errors. The ITT estimates are well justified and consistently applied, and attrition was low (about 20%) with no evidence of differential attrition across arms. Causal claims within the sampled, phone-reachable populations are therefore highly credible. Internal validity is further reinforced by high engagement rates (70–80%) that limit concerns about selective compliance, by the replication of impacts across five countries, and by the embedded randomization of NGO versus government implementers in Nepal and the Philippines, which allows for clean causal inference on delivery models.

My reading of the paper is less sympathetic when it comes to the external validity claims. What the study does convincingly show is that interactive phone tutoring worked for the sampled populations across multiple countries and delivery channels in the time of COVID, or more generally when schools were closed. Where I think the paper overreaches is in presenting this as a universally generalizable resilient systems solution.

The main issue is that any intervention capable of sustaining steady-state school learning during closures could have generated large relative effects. There is little in the results that suggested phone tutoring per se, rather than the mere continuation of structured learning that drove the outcomes. Indeed, the reported 0.3–0.35 SD effect sizes line up with what we already know about pandemic learning losses. In my own longitudinal work in Malawi (Asim et al. 2024), students had lost about 0.5 SD by the time schools reopened after seven months, and these losses compounded to nearly 0.8 SD within the following half year as the gradient of learning slowed. By comparison, the Angrist et al. results suggest that tutoring helped children recover roughly half of what they would have learned in steady-state schooling — a valuable result, but not evidence of a transformative intervention.

Framed this way, the program is promising as an emergency response tool among others, but not yet proven as a generalizable model for all emergency situations. Unless the authors can show that the effects exceed the magnitude of lost learning in these countries, or that phone tutoring clearly outperforms credible alternatives such as TaRL-style community camps, the claim that this is the most effective or scalable solution feels overstated. A more balanced framing would recognize it as one plausible approach to mitigating learning collapse in crises rather than a universal blueprint for a resilient education system. It would also help if the authors benchmarked their results explicitly against pre-COVID annual learning gains in these contexts and situated their findings alongside other crisis-response interventions. Finally, I would encourage them to discuss more openly how the choice to standardize scores at the control endline shapes interpretation. In a setting where control groups are collapsing, these scaling choices are not trivial, and robust checks against baseline or pooled SDs would give readers more confidence in how to interpret the magnitude of the results.

Originality/Significance of Contributions

The real strength of the paper lies in its scale and execution — coordinating five RCTs across countries and delivery channels under COVID is impressive and goes further than most education trials I have reviewed. The authors should feel accomplished in having delivered a well-executed set of trials, but they need not exaggerate the claims. In my view, the underlying idea is not novel: the model builds on the well-established principle of targeted instruction popularized by TaRL, though the authors adapted it to a phone-based, one-on-one format during school closures. The real significance is in showing that such a model can be implemented at scale (though it is important to note that the India Karnataka rollout is only quasi-experimental), at relatively low cost, and in COVID closure conditions with reasonably consistent results. Framed this way, the contribution is valuable, but more modest than the current narrative suggests.

On significance, the interpretation is more fragile than the narrative suggests. The apparent consistency of results is in large part an artefact of the crisis context — the study was conducted in regions like South Asia and Sub-Saharan Africa where school closures were unusually long and learning losses particularly steep (Patrinos et al.). In such settings, any intervention that sustained even modest levels of learning would show large relative effects. Moreover, the cross-country estimates in the paper are far from uniform: Uganda

reports an effect close to 0.9 SD, the Philippines about 0.45 SD, while India, Kenya, and Nepal are closer to 0.3 SD. This spread in magnitudes raises important questions about why an intervention framed as highly effective yielded such different impacts across countries. It suggests the results are less universally stable than implied and remain heavily contingent on local conditions and the depth of learning collapse.

Interpretation of Results

In Section VI.E the authors present an external validity analysis by testing whether conditional treatment effect estimates from one country can predict impacts on another. Their own results show the opposite of what their narrative suggests: extrapolations fail badly. For example, extrapolating from Uganda's 0.89 SD effect produces implausibly high predicted impacts in other countries, while India's more modest 0.22 SD effect is mis-predicted by large margins — in some cases even yielding negative expected effects, such as India to Nepal (-0.07 SD). The clear finding is that baseline covariates cannot explain treatment heterogeneity, and that treatment effects do not transport cleanly across contexts.

This validity exercise needs to be interpreted for what it shows: the intervention's impacts are highly contingent on local contexts and cannot be assumed to generalize. If anything, the stress test undercuts the broad claims of consistency and resilience — it highlights the fragility of the results across settings. The true policy-relevant takeaway is sobering: a policymaker cannot be sure what impact to expect in their own context. And if that is the case, how can the intervention be generalized as a blueprint for education emergencies? The only claim that seems defensible is narrower: in contexts where learning collapses as it did under COVID, phone tutoring can help recover a portion of the lost ground.

## Review of Nature MS 2025-07-17892

**OVERVIEW**

This is a well-written paper on an ambitious set of experiments that aimed to address the educational emergency caused by school closures during the COVID-19 pandemic. The authors document the effect of phone-based tutoring and, with less emphasis, SMS-based educational content delivery on learning outcomes of students in primary grades in five distinct settings (Kenya, India, Nepal, Philippines and Uganda), based on a model that had previously been developed and evaluated in Botswana (Angrist et al, Nature Human Behaviour 2022). Using short phone-based assessments, they document impressive effect sizes on student achievement of ~0.3-0.4 standard deviations (s.d.) after about 8 weekly lessons of 20-minutes each. These aggregate estimates hide substantial heterogeneity ranging from between 0.085 s.d. in Kenya to 0.89 s.d. in Uganda for the same intervention. The authors make the case that the program, and the evidence, are both relevant beyond the COVID-19 pandemic, with applicability also to other emergencies that occur periodically in many parts of the world — they provide suggestive evidence from Philippines and India to this effect. In specific settings, the authors also test some sub-randomizations (such as NGO versus government delivery) but these are secondary to the main focus of the trials.

**SUMMARY OF COMMENTS**

There is a lot to like in this paper. I especially appreciated both the immense efforts of the authors in setting up the core intervention in multiple countries, with multiple partners in the challenging setting of the pandemic, as well as the commitment and flexibility to subject each iteration of the program to a randomized control trial. The core result — that the composite phone-based tutoring intervention was effective in raising achievement — is clear and unlikely to be sensitive. Changes I recommend below are mostly expositional and not primarily centered on the scientific validity of the authors' claims (although they matter for clarity and transparency).

To my mind, it is clear that this paper deserves to be published in a reputed general interest journal. The specific need that the intervention addresses (remote instruction for foundational learning in low/middle-income settings) is widespread and, as the authors demonstrate, the specific intervention can also be adapted across settings. These authors were at the forefront of the literature on remote instruction during COVID-19 and, although there were several contemporaneous experiments (including e.g. a 7-country study in Latin America, plus others in Spain, Italy, India etc.) with mostly positive effects, I think they can legitimately claim to be the leaders in that space. There is some policy momentum now behind exploring remote tutoring outside of COVID-19 settings, including the scale-up discussed in this paper, and I think at least some of this reflects the dissemination of the research discussed in this paper. So, it is clear that this stream of work has been influential.

Whether the specific scientific innovation is adequate for *Nature,* and whether the paper represents a sufficient advance over the knowledge in the first experiment in *Nature Human Behaviour* (Angrist et al, 2022) and the many other contemporaneous studies, is a decision for the Editor and I will not have comments in that direction. My comments below focus on expositional changes that, in my view, are essential for the paper to send out a clear actionable

message for a broad readership. That being said, the Editor would also be in a much better position to judge the requirements for the *Nature* readership than a typical Economics paper.

**MAIN COMMENTS**

**#1 Reduce the number of reported analyses**
The paper would be much better served if it narrowed down the scope. While I appreciate the effort the authors took across multiple distinct empirical analyses, they are not equally important and much of the analysis distracts from their main points.

To my mind, the paper has three main results: (i) phone-based tutoring support was effective in raising student achievement in very diverse settings, (ii) SMS-only interventions were typically not effective and (iii) there is huge heterogeneity in treatment effect sizes across contexts and experiments, which is not explained by measured sources of variation. Everything else is a distraction. The paper would be well-served in only highlighting these.

In particular, I recommend that various sub-treatments (govt vs. NGO), the Philippines typhoon subsection, the analysis of the India scale-up (which is non-experimental, and is half-hearted by the standards of high-quality non-experimental causal evaluations) etc. should all be removed from this draft. So should the validation of the phone survey in Kenya. These are not the features that lead this paper to be of general interest, nor are they necessarily the most robust parts of the analysis.

**#2 Explain the core experiment design and results more clearly**
Getting rid of secondary analyses, and associated exhibits, also frees up space for the authors to expand their exhibits to provide useful insight about the actual core experiments.

There are two issues here that I think are first-order importance:

a) **Details of experiment design:**

1. Move Table A.8 to the main body of the paper. This is not appendix material.
2. Please also indicate the number of treated and control individuals in each experiment separately, as well as details of attrition (for each experiment). I recommend a full CONSORT style diagram for each of the 5 experiments as supplementary material.
3. Please also discuss the representativeness (or not) of the population compared to the broader school-going population in each of these countries. It is not clear from the draft how the sampling frame was identified, how households were approached and whether there were extra selection criteria for the study before the randomization. I suspect there were some such criteria since I note that the response rates for the surveys are considerably in excess of those found by other research teams from lists of phone numbers in developing countries during COVID-19.
4. I note that in the Declaration accompanying the submission, the Corresponding Author confirms that the paper reports "the exact sample size (n) for each experimental group/condition, given as a discrete number and unit of measurement " (emphasis mine). I apologize if I have missed it but I did not find this information. Specifically, I cannot on the basis of Section III. C, Section III.D or Appendix Table A.8 answer just how many students were randomized to be treated in each of the individual experiments? In the

countries which featured further sub-randomization (e.g. NGO vs. government implementation), I could not tell whether these were randomized with equal probability or not. As a summary measure, I cannot tell here what the total number of treated individuals across the 5 studies was. These are crucial details for interpreting this experiment, and also for comparing with the many other studies featuring remote instruction. The authors should include this in the main body of the paper.

5. Please note that, while I classify these comments as expository, issues of sampling, representativeness and attrition for each experiment individually go to the heart of the scientific validity of the claims (and the implications for policy). It is essential that this is fully specified in at least the supplementary material for readers.

**b) Transparency in presentation of results across experiments**

1. The greatest strength of this study is the running of 5 distinct experiments across settings. Given this, I do not understand the choice to present results in the aggregated (pooled) sample as the main specification.
2. As I see it, your main result is Table 4 and that should be your key exhibit.
3. The second table that should be emphasized as the "main" result is Table 5 but with a separate panel for each experiment.
    a. Please do not pool across experiments in this manner, especially given different settings, sampling protocols, implementation instructions and randomization procedures — doing this properly would require very different Bayesian approaches (as Meager (2019, AEJ:Applied) demonstrated for experiments on microfinance). In that table, please also have the baseline value and the control mean for each individual country sample in each panel.
    b. This is very important since we do not have any indication in the manuscript about differences in baseline achievement across your samples (i.e. differences between e.g. Philippines and Kenya, not the treated and control groups within each country). Given the coarse categorization that is possible with these short ASER type assessments, it is possible that much of the difference across experiments comes from a "standard deviation" being very different across each of the individual samples. It would be far more transparent to present actual proficiency gains in the 5 samples separately. Formatted suitably, this could be the key exhibit of the paper, easily contained in a single (full-page) exhibit.
    c. This also would be a more principled way of dealing with the coarseness of the ordinal ASER-type measures. For an ordinal variable that only takes 4-5 categorical values, the standard deviation metric seems particularly difficult to interpret. This is even more so when comparing across vastly different contexts. I understand that you have to report the z-score results somewhere but it would be far more informative to see the competence-level results than the aggregated scores.

4. I would recommend dropping the section of scale up in India entirely. It detracts from the core of the paper and does not speak directly to the rest of the paper — I suspect the authors agree, which is why the entire section only leads to Appendix exhibits and not a single exhibit in the main body of the paper.
    a. Incidentally, the issues of ordinality are especially a concern with the difference-in-difference approach in the analysis of the scale-up. Analysis of

changes only makes sense with an implicit assumption of cardinality: a change from 1 to 2 has to equal the change from 3 to 4. This would be okay if this scale was approximately equal-interval but it is not. Ordinality is, of course, a broader problem when it comes to test scores (see, e.g., Bond and Lang 2013) but it is especially severe for short assessments that only permit a relatively coarse categorization.

   b. It is possible, given the data, to write a much more compelling non-experimental evaluation of the scale-up. However, the space it would take to do this well is not feasible in the current paper. Nor do I think it adds to the clarity of the paper to have that also tacked on without doing full justice.

5. For transparency, I would also recommend being upfront about whether any of the analyses were pre-registered or not. Please note that I do not think there is anything fishy here – the analyses and outcomes are pretty standard but I suspect it is worth clarifying this in the draft as such pre-registration has moved to being increasingly common in the field. I don't think anybody would object to the lack of a full PAP while trying to coordinate a multi-country effort in the midst of COVID (when I'm sure you had very little ex ante knowledge of how well implementation would occur). I suggest this only for transparency.

**#3 [OPTIONAL] Consider a crisp, short paper**

This is purely a suggestion, offered with free disposal. In my view, the signal achievement of this project is to have conducted 5 experiments with multiple partners across countries in the midst of a severe education crisis. Communicating the very positive results from this endeavour should, in my view, be the single purpose of this paper.

For this single purpose, it is adequate to just report on the design of the core experiments and the main results on learning outcomes. Effectively, that means three tables: (i) to show sample characteristics and balance; (ii) to show effects on the aggregated standard deviation metric (currently Table 4); and (iii) to show effects on individual competences (Table 5, but by country). Everything else should go to supplementary materials.

If *Nature* allows, I would recommend a well-fleshed out Discussion section where you can discuss relevance to (i) other emergencies, (ii) post-COVID FLN, (iii) scale-ups, (iv) mechanisms etc. But these are NOT the main value-added of this paper or this effort.

Given that the paper is in consideration for a broad readership, much beyond just economists, I think a crisp 5000 word paper with 3-4 exhibits would be far more influential in the world than a bloated paper with too many secondary analyses written for an Economics audience.
